# Towards single-crystalline two-dimensional poly(arylene vinylene) covalent organic frameworks

**DOI:** 10.1038/s41557-025-02048-8

**Published:** 2026-01-20

**Authors:** Shaik Ghouse, Ziang Guo, Sergio Gámez-Valenzuela, David Mücke, Bowen Zhang, Lei Gao, Silvia Paasch, Yubin Fu, Chuanhui Huang, Chandrashekar Naisa, Eike Brunner, Mischa Bonn, M. Carmen Ruiz Delgado, Junliang Sun, Ruqiang Zou, Ute Kaiser, Mingchao Wang, Xinliang Feng

**Affiliations:** 1 Faculty of Chemistry and Food Chemistry and Center for Advancing Electronics Dresden, Technische Universität Dresden, Dresden, Germany; 2 School of Materials Science and Engineering, Peking University, Beijing, China; 3 College of Chemistry and Molecular Engineering, Beijing National Laboratory for Molecular Sciences, Peking University, Beijing, China; 4 Department of Physical Chemistry, University of Málaga, Málaga, Spain; 5 Instituto Universitario de Materiales y Nanotecnología, IMANA, University of Málaga, Málaga, Spain; 6 Central Facility for Materials Science Electron Microscopy, Ulm University, Ulm, Germany; 7 Fraunhofer Institute for Ceramic Technologies and Systems, Dresden, Germany; 8 Max Planck Institute for Polymer Research, Mainz, Germany; 9 Max Planck Institute for Microstructure Physics, Halle (Saale), Germany; 10 State Key Laboratory of Advanced Waterproof Materials, Guangdong Provincial Key Laboratory of Nano-Micro Materials Research, School of Advanced Materials, Peking University Shenzhen Graduate School, Shenzhen, China

**Keywords:** Electronic materials, Conjugated polymers, Polymer synthesis

## Abstract

Vinylene-linked two-dimensional (2D) conjugated covalent organic frameworks, or 2D poly(arylene vinylene)s (2D PAVs), are promising polymer semiconductors for (opto-)electronics, photocatalysis and electrochemistry. However, conventional solvothermal synthesis often produces 2D PAVs that are poorly crystalline or difficult to access. Here we introduce a Mannich-elimination strategy that converts 8 2D imine-covalent organic frameworks into 11 highly crystalline 2D PAVs though a reversible C=C bond formation mechanism enabling precise crystallization control. This versatile approach affords robust 2D PAVs with honeycomb, square or kagome lattices, specific surface area up to ∼2,000 m^2^ g^−1^ and lattice-mismatch tolerance up to 3.5%. High-resolution transmission electron microscopy and continuous rotation electron diffraction reveal molecular-level ordering in a 2-µm-sized triphenylbenzene-based single-crystalline 2D PAV. We demonstrate that crystallinity profoundly influences charge transport, with benzotrithiophene-based 2D PAVs exhibiting charge mobilities tenfold higher than their amorphous analogues or 2D polyimine precursors. This work establishes a general route to highly crystalline 2D conjugated polymer materials for robust applications.

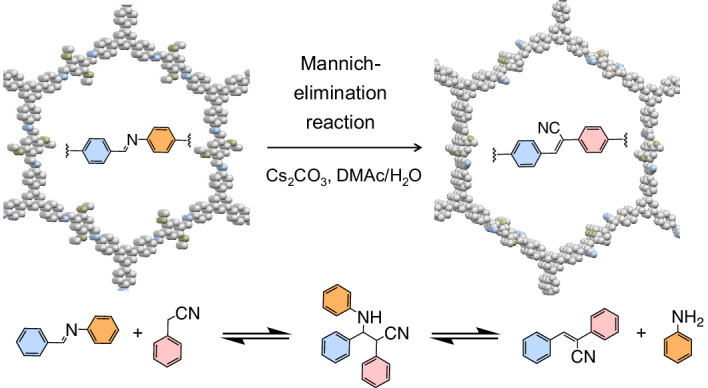

## Abstract (web summary)

Two-dimensional poly(arylene vinylene) frameworks are promising polymer semiconductors, yet obtaining highly crystalline materials is a major challenge. Now a series of 11 highly crystalline or single-crystalline 2D poly(arylene vinylene)s have been prepared—from 2D imine-linked covalent organic frameworks through a Mannich-elimination strategy—with diverse lattices, enhanced conjugation and specific surface areas up to 2,000 m^2^ g^−1^.

## Main

Two-dimensional conjugated covalent organic frameworks (2D c-COFs)^1–4^, or layer-stacked^5–7^, crystalline 2D conjugated polymers^8,9^, represent a unique class of organic 2D crystals with extended in-plane π-conjugation^10^ and out-of-plane electronic couplings^11^. Typical 2D c-COFs are in-plane interconnected^12,13^ by conjugated linkages such as imine^14–17^ and pyrazine^11^. However, the polarized C=N bonds^18,19^ hinder efficient π-electron delocalization, often leading to large optical band gaps and inefficient charge carrier transport. Recent advances in vinylene- or *sp*^2^-carbon-linked 2D c-COFs^20,21^, that is, layered 2D poly(arylene vinylene)s (2D PAVs)^22^, have demonstrated significantly enhanced π-conjugation compared with imine-linked 2D c-COFs (also known as 2D polyimines, 2D PIs)^23^. Benefitting from their tunable topologies^24,25^, tailored electronic structures^26^, intrinsic charge carrier mobilities^8,22^ and abundant active sites^27^, these materials have attracted considerable attention in applications in (opto-)electronics^10,28^, photocatalysis^23,26,29–31^ and electrochemistry^32^.

Since the first report of crystalline 2D PAV via Knoevenagel 2D polycondensation^20,21^, various synthetic methodologies, including Aldol-type^33–35^, Horner–Wadsworth–Emmons^36^, Wittig^37^ or Claisen–Schmidt^38^ 2D polycondensation reactions, have been developed to construct this class of materials. However, the achieved domain sizes via these methods are generally below 20 nm, limiting their potential in wide-scope applications. Moreover, unlike the excellent generality of the well-established Schiff-base 2D polycondensation^39–42^, only selected 2D PAVs are crystalline, owing to the considerably lower reversibility of C=C bond formation compared with C=N bond formation. In this context, it remains highly challenging to synthesize 2D PAVs with robust topologies and high crystallinity (for example, domain size >100 nm), which requires a deep understanding of reaction kinetics and precise control over reaction reversibility.

Here, we introduce a Mannich-elimination reaction strategy to synthesize 11 highly crystalline or single-crystalline 2D PAVs in honeycomb, square or kagome lattice structures from the 2D PI precursors. By contrast, the conventional Knoevenagel reaction approach only produces amorphous polymers for some of these systems. The cascade Mannich-elimination reaction mechanism is elucidated through model reactions at the (supra)molecular level and density functional theory (DFT) calculations. The successful synthesis of 2D PAVs is demonstrated by various ex situ spectroscopic techniques, powder X-ray diffraction (PXRD) and high-resolution transmission electron microscopy (HR-TEM). The molecular-level structure of a 2-µm-sized single-crystalline honeycomb 2D PAV (**2DPAV-DMP-TPB**, where DMP is dimethoxybenzene and TPB is triphenylbenzene) is resolved by HR-TEM and continuous rotation electron diffraction (cRED). It is noteworthy that the Mannich-elimination shows a certain lattice-mismatch tolerance (up to 3.5%) between 2D PAV and its 2D PI precursor. The specific surface areas of these 2D PAVs can be more than 100 times greater than those produced by the traditional Knoevenagel reaction. We further demonstrate that the crystallinity has a large impact on the charge transport properties, as exemplified by benzotrithiophene (BTT)-based honeycomb 2D PAVs, which exhibit charge mobilities ten times greater than the amorphous 2D PAV and their corresponding 2D PIs. This work opens an avenue for the efficient synthesis of highly crystalline 2D PAVs, suitable for robust applications.

## Results and discussion

### Model compounds and 2D PAVs by Mannich-elimination reaction

**Fig. 1:**
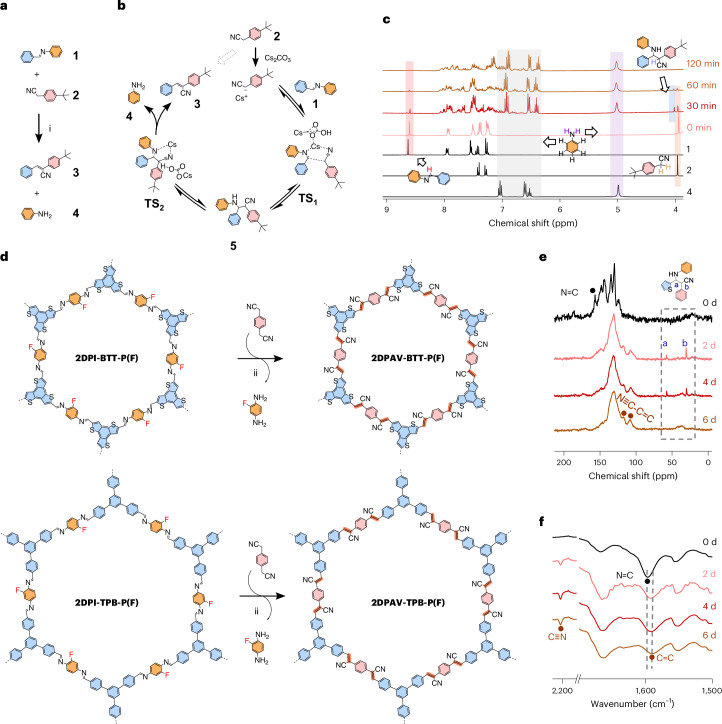
Model compounds and 2D PAVs by Mannich-elimination reaction. **a**, Schematic synthesis of model compound **3**. (i) Cs_2_CO_3_, DMAc/H_2_O (10/1), 120 °C, 8 h. **b**, Proposed reaction mechanism for the Mannich-elimination pathway. **c**, In situ NMR analysis of model reaction performed in DMAc. The spectra of compounds **1**, **2** and **4** measured in DMSO-d_6_ are shown for comparison. The in situ spectra are recorded in DMAc with trace DMSO-d_6_ for calibration. **d**, Schematic synthesis of **2DPAV-BTT-P(F)** and **2DPAV-TPB-P(F)** from the F-labelled 2D PIs. (ii) Cs_2_CO_3_, DMAc/H_2_O (7/3), 120 °C, 5–6 days. **e**,**f**, Time-dependent solid-state ^13^C CP NMR (**e**) and FT-IR (**f**) spectra during the synthesis of **2DPAV-BTT-P(F)** (lines for 6 days) from **2DPI-BTT-P(F)** (lines for 0 days). Source data

The Mannich reaction has been widely utilized in the synthesis of bioactive compounds^43^. When combined with the elimination process, the Mannich-elimination reaction (also known as retro-Michael addition-elimination reaction) enables facile C=C bond formation using either a base (for example, KOH) or a Lewis acid (for example, In(OTf)_3_) as a catalyst^44^. To assess the feasibility of this reaction for synthesizing 2D PAVs, we investigated the reaction mechanism through a series of model reactions and DFT calculations. In a representative model reaction, the Mannich-elimination reaction between (*E*)-*N*,1-diphenylmethanimine (**1**) and 2-(4-(*tert*-butyl)phenyl)acetonitrile (**2**) involves a two-step process (Fig. 1a,b): C–C bond formation between the imine and the active methylene to generate the intermediate addition product **5**, followed by the elimination process towards the formation of the cyano-vinylene bond in (*Z*)-2-(4-(*tert*-butyl)phenyl)-3-phenylacrylonitrile (**3**) with aniline (**4**) as the by-product.

Aiming to optimize the reaction reactivity, we performed the model reaction in a mixed solvent of dimethylacetamide (DMAc)/H_2_O (v/v = 10/1) at 120 °C for 8 h under different catalytic conditions (2 equivalents of catalyst per C=N bond; see the synthetic details in the Supplementary Information). The results suggest that compound **3** can be obtained in 10–35% yield in the presence of mild bases such as NaOAc, KOAc, CsOAc, Na_2_CO_3_ and K_2_CO_3_. However, the yield significantly increases when using KOH (86%) or Cs_2_CO_3_ (98%) as a catalyst. By contrast, only hydrolysis of **1** occurs, and no product **3** was detected under acidic conditions (see the results for acids and other (in)organic bases in Supplementary Table 1). We further explored the role of the solvent under optimal conditions. The results reveal that mere DMAc (98%) and 1,3-dimethyl-2-imidazolidinone (91%) efficiently promote the reaction. At the same time, other polar solvents (for example, acetonitrile and dimethylformamide) provide yields ranging from 18% to 60%, while low-polar solvents (for example, dioxane and mesitylene) hinder the reaction with a yield less than 1%.

To clarify whether the reaction follows a Mannich-elimination mechanism or a pathway involving imine hydrolysis to an aldehyde and subsequent Knoevenagel condensation, we performed in situ ^1^H nuclear magnetic resonance (NMR) analysis using Cs_2_CO_3_ as the catalyst and DMAc/H_2_O as the solvent. The spectra display a decreased intensity of the imine (**1**) and cyano-methylene (**2**) protons at 8.6 ppm and 3.9 ppm, respectively, as accompanied by the gradual appearance of the amine proton (**4**) at 5.0 ppm over 240 min (Supplementary Fig. 1; see also Fig. 1c). However, no aldehyde signal was observed, indicative of the cascade Mannich-elimination reaction pathway. This is further verified by the synthesis of compound **3** in 98% yield in DMAc without the addition of H_2_O, which excludes the hydrolysis of imine (Supplementary Table 1). The reaction is largely facilitated in DMAc compared with that in DMAc/H_2_O (Supplementary Figs. 2 and 3), probably due to reduced reversibility of the C=C bond formation in the absence of water.

Moreover, the formation of the intermediate addition product **5** was detected by ^1^H NMR with characteristic cyano-C–H (ref. ^45^) proton signal at 4.06 ppm observed after 30 min of reaction, which fully disappeared after 120 min owing to the formation of product **3** (Fig. 1c and Supplementary Fig. 2; the ^1^H and ^13^C NMR spectra of isolated **5** are shown in Supplementary Fig. 4). The intermediate **5** was further verified by various mass spectrometry analyses (Supplementary Figs. 5 and 6), which supports our proposed reaction mechanism in Fig. 1b. This mechanism is further elucidated by DFT calculations on the relative Gibbs energy. When only DMAc is used as the solvent, the formation of **5** is energetically very favourable (Supplementary Fig. 7), while its transformation into the key transition state (TS_2_ in Fig. 1b) during the elimination process is the rate-determining step of the reaction. In comparison, the presence of water results in relatively sluggish reaction kinetics for the formation of the TS_1_ transition state in the Mannich step, accounting for the experimentally observed slower reactions. The presence of water is expected to improve the reversibility of C=C bond formation, which is essential for achieving high structural ordering in 2D PAVs. Meanwhile, water does not affect the calculated energy barriers for the formation of TS_2_ (Supplementary Fig. 7).

We thus envision that the Mannich-elimination reaction between crystalline 2D PIs and symmetric acetonitrile monomers would lead to 2D PAVs with a well-maintained structural order of the 2D PI precursors. To probe this hypothesis, we synthesized two F-labelled crystalline 2D PIs, that is, **2DPI-BTT-P(F)** (P = phenyl; all polymers in this work are named A–B–C, where A is 2DPI or 2DPAV, B is the aldehyde building block and C is the amine or methylene building block) and **2DPI-TPB-P(F)**, via Schiff-base polycondensation in solvothermal syntheses. Then, we monitored their transformation with the *C*_2_-symmetric 1,4-phenylenediacetonitrile into **2DPAV-BTT-P(F)** and **2DPAV-TPB-P(F)** (Fig. 1d) by solid-state ^19^F/^13^C cross-polarization (CP) NMR and Fourier-transform infrared (FT-IR) spectroscopies. Taking **2DPAV-BTT-P(F)** as an example, the ex situ ^13^C CP NMR spectra show peaks at 57 and 30 ppm for the isolated polymers after 1 day of synthesis, which correspond to the formation of the intermediate addition product (Fig. 1e and Supplementary Fig. 8). These peaks gradually vanish over 6 days, accompanied by the disappearance of the C=N signal at 157 ppm for **2DPI-BTT-P(F)** and the appearance of a cyano-vinylene peak at 117 and 108 ppm. The solid-state ^19^F NMR spectra show no ^19^F peak in the resultant **2DPAV-BTT-P(F)** (Supplementary Fig. 9), indicative of complete consumption of **2DPI-BTT-P(F)** after the synthesis. Ex situ FT-IR spectra show that the stretching vibration of the imine bond at 1,597 cm^−1^ gradually shifts to 1,590 cm^−1^ (C=C) owing to the formation of cyano-vinylene linkages in **2DPAV-BTT-P(F)** (Fig. 1f and Supplementary Fig. 10). In addition, the C≡N peak is observed at 2,214 cm^−1^. The solid-state ^19^F/^13^C CP NMR and FT-IR spectra of **2DPAV-TPB-P(F)** are shown in Supplementary Figs. 9 and 11.

### Highly crystalline 2D PAVs in different lattices by Mannich-elimination reaction

**Fig. 2:**
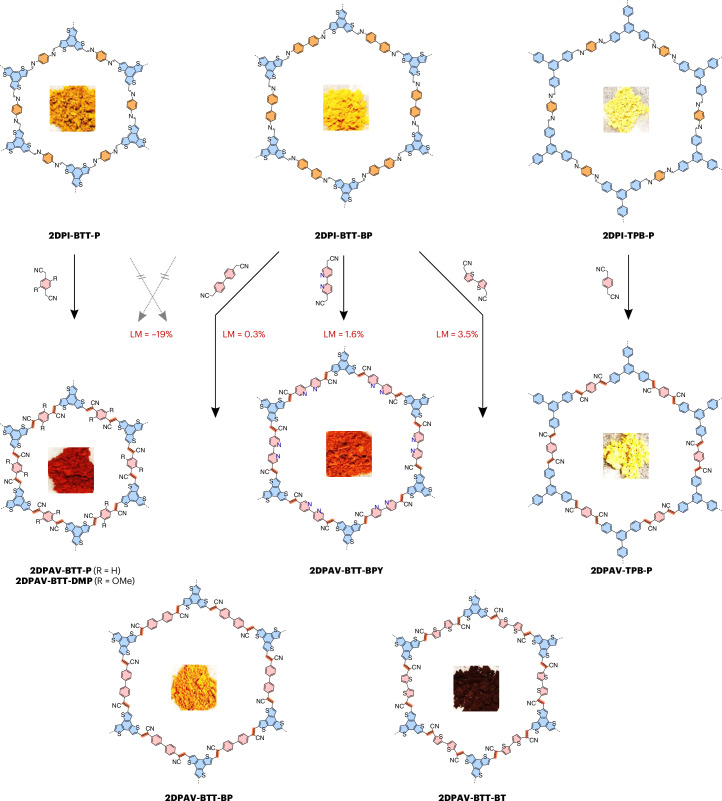
Schematic synthesis of various honeycomb 2D PAVs by Mannich-elimination reaction. Synthetic condition of 2D PAVs: Cs_2_CO_3_, DMAc/H_2_O (7/3), 130 °C, 6 days. The pictures within each of the structures are photos of the powder obtained.

**Fig. 3:**
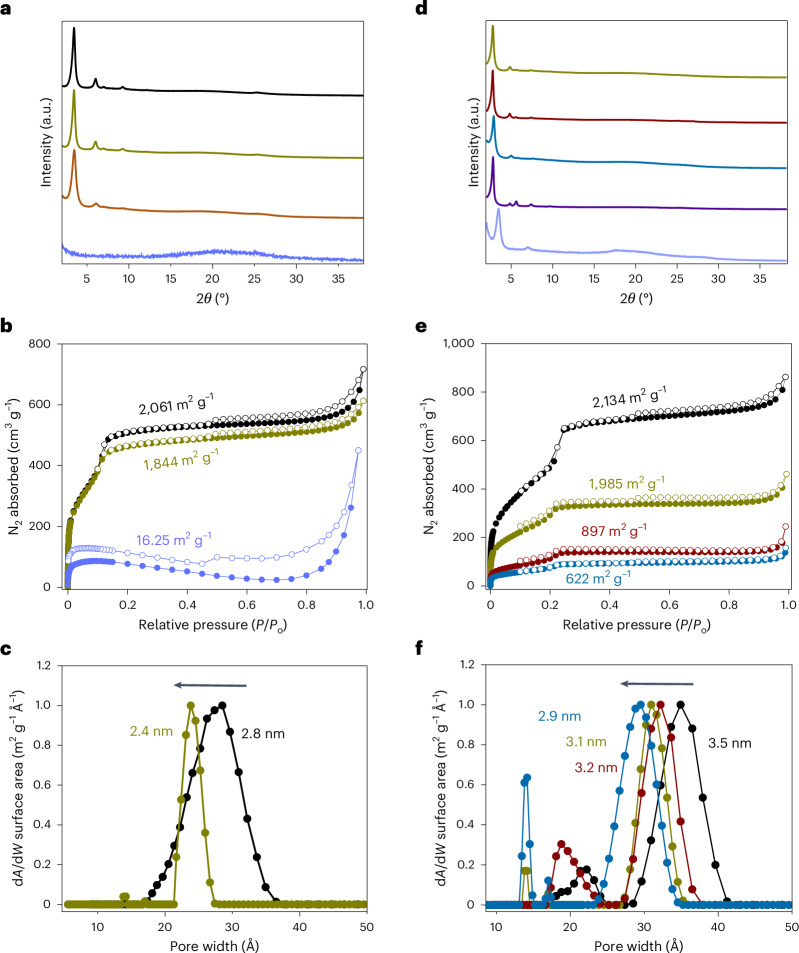
Crystal and pore structures of various 2D PAVs. **a**–**c**, PXRD patterns (**a**), N_2_ physisorption isotherms (**b**) and pore size distributions (**c**) for **2DPI-BTT-P** (black), **2DPAV-BTT-P** (olive), **2DPAV-BTT-P(F)** (orange) and **2DPAV-BTT-P(Kn)** (light violet). **d**–**f**, PXRD patterns (**d**), N_2_ physisorption isotherms (**e**) and pore size distributions (**f**) for **2DPI-BTT-BP** (black), **2DPAV-BTT-BP** (olive), **2DPAV-BTT-BPY** (dark red), **2DPAV-BTT-BT** (cyan), **2DPAV-DMP-TPB** (dark violet) and **2DPAV-HATN-P** (light violet). Source data

Encouraged by the above results, we synthesized the unlabelled powder-based **2DPI-BTT-P** and **2DPI-TPB-P**, which exhibit dark-yellow and light-yellow colours (Fig. 2), respectively. They display enhanced crystallinity compared with F-labelled 2D PIs (Supplementary Fig. 12) due to the high symmetry of the unlabelled building block, which would result in better layer stacking. Using the above Mannich-elimination approach, **2DPAV-BTT-P** and **2DPAV-TPB-P** were subsequently obtained as red and light-yellow powders, respectively (Fig. 2; see their FT-IR, Raman and ultraviolet–visible (UV–vis) absorption spectra as well as scanning electron microscopy (SEM) images in Supplementary Figs. 13–19; the PXRD pattern of **2DPAV-TPB-P**, **2DPAV-TPB-P(F)** and their 2D PIs is shown in Supplementary Fig. 20). PXRD analysis reveals the crystalline nature of **2DPAV-BTT-P** with distinct 2*θ* signals at 3.45° (corresponding to 2.56 nm), 6.03°, 7.00°, 9.42°, 12.16° and 25.25° (Fig. 3a, red line), which we assign to the (100), (110), (200), (210), (220) and (001) crystallographic planes, respectively, for an AA stacking geometry (see the Pawley refinement in Supplementary Fig. 21). The full width at half maximum (FWHM) of the (001) peak is determined to be 0.39°. As expected, **2DPAV-BTT-P(F)** exhibits similar PXRD signals but with a larger FWHM value of 0.55° (Fig. 3a, brown line), indicative of an inferior crystallinity in the material derived from the F-labelled 2D PI. In stark contrast, the directly synthesized sample, namely **2DPAV-BTT-P(Kn)**, through Knoevenagel polycondensation between benzo[1,2-b:3,4-b′:5,6-b″]trithiophene-2,5,8-tricarbaldehyde and 1,4-phenylenediacetonitrile is amorphous. It is also noted that performing the Mannich-elimination reaction on **2DPI-TPB-P** in the absence of water does not lead to the expected **2DPAV-TPB-P**, further highlighting the critical role of water in enhancing the reaction reversibility towards crystalline materials.

HR-TEM imaging shows the well-defined honeycomb lattice of **2DPAV-BTT-P** (Supplementary Fig. 22). N_2_ physisorption measurements were further performed to elucidate the pore characteristics of the polymer frameworks. Both **2DPI-BTT-P** and **2DPAV-BTT-P** show type I isotherms, with Brunauer–Emmett–Teller (BET) surface areas of 2,061 m^2^ g^−1^ and 1,844 m^2^ g^−1^, respectively. By contrast, the amorphous **2DPAV-BTT-P(Kn)** exhibits a surface area as small as 16 m^2^ g^−1^ (Fig. 3b). The pore size distribution calculated by the non-local DFT method reveals a pore diameter reduction from 2.8 nm in **2DPI-BTT-P** to 2.4 nm in **2DPAV-BTT-P** attributable to the presence of cyano groups in the pore channels of the latter (Fig. 3c; see more details in Supplementary Figs. 23 and 24). These results support our strategy for constructing highly crystalline 2D PAVs, especially those that cannot be directly synthesized by Knoevenagel polycondensation, via the Mannich-elimination reaction on well-defined 2D PIs.

To explore the universality of this strategy, we first synthesized **2DPAV-BTT-DMP** from **2DPI-BTT-P** and 2,2′-(2,5-dimethoxy-1,4-phenylene)diacetonitrile (Fig. 2 and Supplementary Fig. 25). We further extended the approach to obtain **2DPI-BTT-BP** (BP = biphenyl) and then **2DPAV-BTT-BP** with an enlarged lattice parameter. More interestingly, by using 2,2′-([2,2′-bipyridine]-5,5′-diyl)diacetonitrile and 2,2′-([2,2′-bithiophene]-5,5′-diyl)diacetonitrile, which share a similar core length to 2,2′-([1,1′-biphenyl]-4,4′-diyl)diacetonitrile, we synthesize a series of BTT-based 2D PAVs with distinct electronic structures from **2DPI-BTT-BP** (yellow powders) via the Mannich-elimination reaction: **2DPAV-BTT-BP** (in light-orange colour), **2DPAV-BTT-BPY** (in dark-orange colour; BPY = bipyridine) and **2DPAV-BTT-BT** (in dark-brown colour; BT = bithiophene) as depicted in Fig. 2. The successful synthesis of materials is demonstrated by FT-IR and solid-state ^13^C CP NMR, as well as Raman spectroscopies (Supplementary Figs. 26–29). The experimental Raman spectra are nicely reproduced by theoretical calculations, which indicate a gradual shift of the C=C stretching vibration from 1,585 cm^−^^1^ in **2DPI-BTT-BP** to 1,438–1,583 cm^−1^ for the 2D PAVs due to enhanced 2D conjugation (see more detailed discussion in Supplementary Fig. 29). DFT calculations reveal dihedral angles between the *C*_2_ building block and the vinylene linkage (Supplementary Figs. 30–32) following the sequence of **2DPI-BTT-BP** (ca. 30°) > **2DPAV-BTT-BP** > **2DPAV-BTT-BPY** > **2DPAV-BTT-BT** (smaller than 5°). These results highlight the high feasibility of finely tuning the chemical and electronic structures of 2D conjugated polymers using the Mannich-elimination synthetic strategy.

The lattice mismatch (LM) between **2DPI-BTT-BP** and the 2D PAVs can be tolerated up to 3.5% (Fig. 2), and **2DPAV-BTT-BP**, **2DPAV-BTT-BPY** and **2DPAV-BTT-BT** are highly crystalline, while the directly synthesized materials are amorphous (Supplementary Figs. 33–35). PXRD patterns display that the (100) peak of **2DPI-BTT-BP** shifts from 2.80° (corresponding to 3.15 nm) to 2.79°, 2.76° and 2.90° for the BP/BPY/BT-bridged 2D PAVs, respectively (Fig. 3d and Supplementary Fig. 36); the FWHM value changes slightly from 0.25° to 0.27°, 0.26° and 0.28°, respectively. We further studied the role of LM by synthesizing **2DPAV-BTT-BP(LM)** from **2DPI-BTT-P** and **2DPAV-BTT-P(LM)** from **2DPI-BTT-BP** (LM degree of ca. 19%). The former vinylene-linked polymer is amorphous, while the latter shows considerably inferior crystallinity to that of **2DPAV-BTT-P** (Supplementary Fig. 37). These results imply that, to achieve a high structural order in 2D PAV, the acetonitrile monomers need to penetrate the porous channels of the 2D PI, followed by the Mannich-elimination reaction at specific imine sites, without causing much lattice expansion or contraction.

The simulated PXRD pattern and SEM images of **2DPAV-BTT-BP**, **2DPAV-BTT-BPY** and **2DPAV-BTT-BT** are shown in Supplementary Figs. 38–41. Their honeycomb polymer frameworks are resolved by HR-TEM images. The distances between adjacent pore centres are 3.1, 3.1 and 2.9 nm, respectively (Supplementary Figs. 42–44), which are consistent with the PXRD results. Their BET surface areas estimated from N_2_ physisorption isotherms are 1,985, 897 and 622 m^2^ g^−1^, respectively; **2DPI-BTT-BP** shows a surface area of 2,134 m^2^ g^−1^ (Fig. 3e). It should be noted that the specific surface area of 1,985 m^2^ g^−1^ achieved for **2DPAV-BTT-BP** represents the record value for the reported 2D PAVs, emphasizing the critical importance of crystallinity. Similar to the above BTT/P-based system, changing from **2DPI-BTT-BP** (3.5 nm) to 2D PAVs also results in a pore size reduction to 3.1 (BP), 3.2 (BPY) and 2.9 (BT) nm (Fig. 3f).

**Fig. 4:**
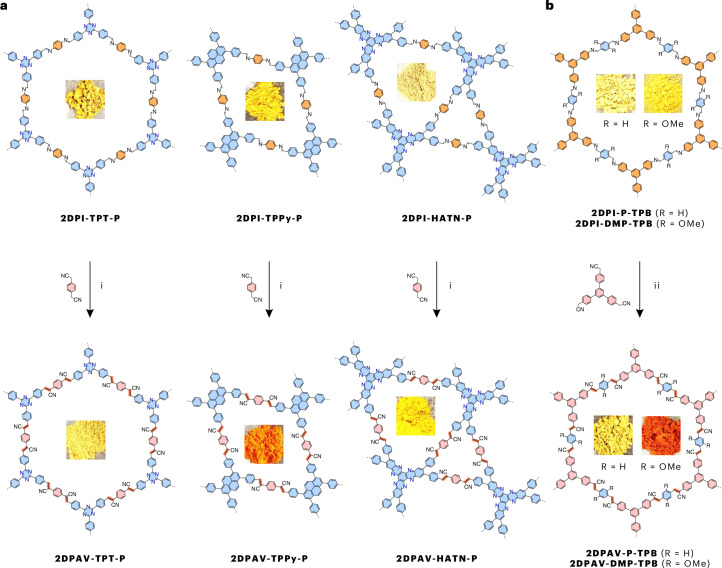
Two-dimensional PAVs by Mannich-elimination reaction showing various lattice and electronic structures. **a**, Honeycomb, square and kagome 2D PAVs synthesized from 2D PIs and *C*_2_-symmetric acetonitrile monomers. (i) Cs_2_CO_3_, 120–130 °C, DMAc/H_2_O, 6–8 days. **b**, Honeycomb 2D PAVs synthesized from 2D PIs and *C*_3_-symmetric acetonitrile monomers. (ii) Cs_2_CO_3_, 120–130 °C, DMAc/H_2_O, 6–12 days. The pictures within each of the structures are photos of the powder obtained.

As shown in Fig. 4a, the Mannich-elimination synthetic strategy is also powerful for the synthesis of three other highly crystalline 2D PAVs in honeycomb, square or kagome lattice based on *C*_2_-symmetric acetonitrile monomers, that is, **2DPAV-TPT-P** (TPT = triphenyltriazine; in yellow colour), **2DPAV-TPPy-P** (TPPy = tetraphenylpyrene; in orange colour) and **2DPAV-HATN-P** (HATN = hexaazatrinaphthalene; in yellow colour). It is significant that their BET surface areas are nearly twice those of the 2D PAVs directly synthesized via the Knoevenagel approach (Supplementary Figs. 45 and 46). Our synthetic strategy can be further extended to construct 2D PAVs based on *C*_3_-symmetric acetonitrile building blocks^46,47^. For instance, honeycomb **2DPAV-P-TPB** and **2DPAV-DMP-TPB** were successfully synthesized as yellow and dark-orange powders, respectively, from 2,2′-(5′-(4-(cyanomethyl)phenyl)-[1,1′:3′,1″-terphenyl]-4,4″-diyl)diacetonitrile (Fig. 4b). However, transformation of a *C*_3_-symmetric building block is more challenging, requiring harsher synthetic conditions—specifically, 8 equivalents of base per C=N bond at 130 °C for 12 days—to achieve full conversion. The characterization of the above five materials by PXRD, solid-state ^13^C CP NMR, FT-IR and Raman, along with related theoretical calculations, is shown in Fig. 3d and Supplementary Figs. 47–52.

### Single-crystalline 2D PAV by Mannich-elimination reaction

**Fig. 5:**
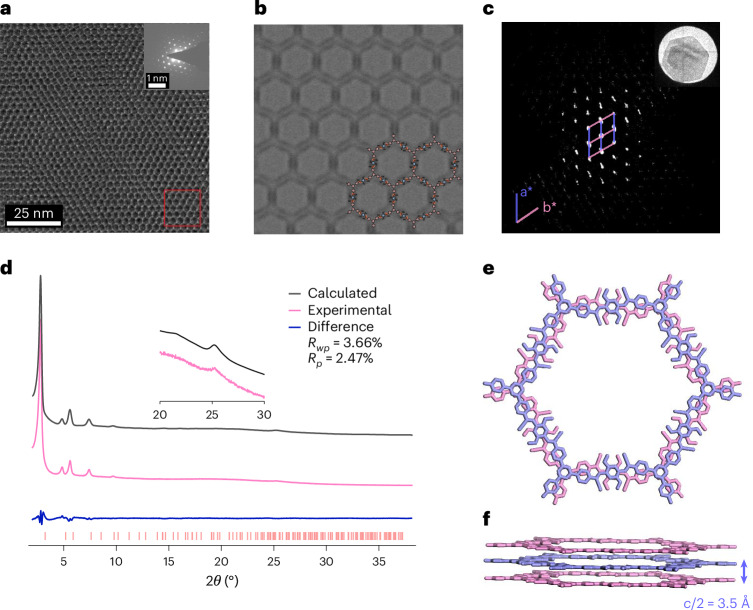
Single-crystalline structure of **2DPAV-DMP-TPB**. **a**, *C*_s_-corrected HR-TEM image along with the SAED pattern. **b**, Simulated HR-TEM image of selected area in **a** (red box). **c**, cRED of a hexagonal 2D PAV crystal. Inset: the crystal on a Cu grid. **d**, Experimental PXRD pattern (pink), Rietveld-refined profile (black) and their difference plots (dark blue). **e**,**f**, Top (**e**) and side (**f**) views of the resolved AA-serrated stacking structure. Source data

We further demonstrated the synthesis of single-crystalline **2DPI-DMP-TPB** under solvothermal conditions^48^. Optical, SEM and TEM images reveal a flake-like morphology for the hexagonal 2D PI crystals, with domain sizes exceeding 2 μm (Supplementary Figs. 53–55). After the Mannich-elimination transformation, the resulting **2DPAV-DMP-TPB** crystal can maintain the hexagonal morphology of the 2D PI precursor showing a domain size of approximately 2 μm (Supplementary Figs. 56 and 57) and a surface area of 1,901 m^2^ g^−1^ (Supplementary Fig. 58). HR-TEM images reveal its honeycomb polymer framework at the molecular level (Fig. 5a; see more details in Supplementary Fig. 59) with a lattice distance of 3 nm, which qualitatively agrees with the simulated structure (Fig. 5b). Supplementary Fig. 60 displays the selected area electron diffraction (SAED) patterns throughout the crystal. Although slightly tilted from the 0001 zone axis, they exhibit the same orientation, indicative of their single-crystalline nature.

cRED collected in low-dose mode at 99 K (Fig. 5c and Supplementary Fig. 61) revealed a hexagonal unit cell with parameters *a* = *b* = 36.55 Å, *c* = 7.13 Å and *β* = 120° for **2DPAV-DMP-TPB**. To validate the crystal structure, the PXRD pattern was simulated from the cRED-derived structural model and further refined by Rietveld analysis (Fig. 5d, Supplementary Fig. 62 and Supplementary Table 2). The refined unit cell parameters are *a* = *b* = 36.505(4) Å, *c* = 7.094(8) Å, *α* = *β* = 90°, *γ* = 120°, with convergence at *R*_wp_ = 3.66% (weighted profile residual factor) and *R*_p_ = 2.47% (profile residual factor). Collectively, these results establish **2DPAV-DMP-TPB** as a honeycomb-structured material adopting an AA-serrated stacking arrangement in the *P*6/*mcc* space group (Fig. 5e).

### Enhanced conjugation and charge transport in crystalline 2D PAVs

**Fig. 6:**
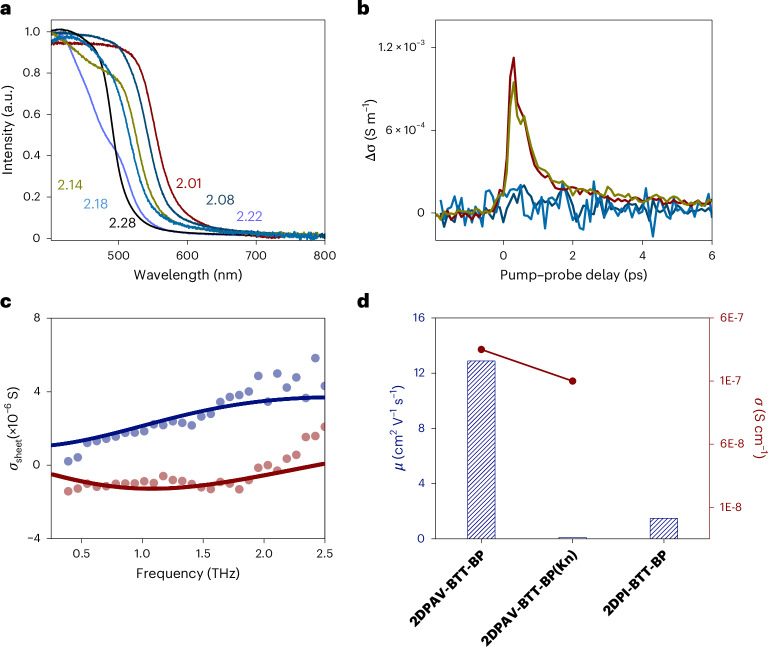
Charge transport properties of 2D PAVs. **a**, UV–vis absorption spectra of **2DPI-BTT-P** (light violet), **2DPAV-BTT-P** (dark red), **2DPAV-BTT-P(Kn)** (dark cyan), **2DPI-BTT-BP** (black), **2DPAV-BTT-BP** (olive) and **2DPAV-BTT-BP(Kn)** (cyan). **b**, Time-resolved terahertz photoconductivity of **2DPAV-BTT-P** (dark red), **2DPAV-BTT-P(Kn)** (dark cyan), **2DPAV-BTT-BP** (olive) and **2DPAV-BTT-BP(Kn)** (cyan). **c**, Frequency-resolved complex terahertz photoconductivity for **2DPAV-BTT-BP**. The solid blue and red lines represent the Drude–Smith fits describing the real and imaginary components of the complex terahertz photoconductivity, respectively. **d**, Comparison of charge carrier mobilities and electrical conductivities of **2DPAV-BTT-BP**, amorphous **2DPAV-BTT-BP(Kn)** and **2DPI-BTT-BP**. Source data

To elucidate the enhanced conjugation in 2D PAV compared with that in 2D PI, we calculated the electronic band structures of monolayer **2DPI-BTT-P, 2DPI-BTT-BP**, **2DPAV-BTT-P** and **2DPAV-BTT-BP**. The 2D PAVs show band gaps nearly 0.2 eV smaller than those of 2D PIs (Supplementary Fig. 63). All monolayers present relatively flat conduction band minima and valence band maxima (Supplementary Fig. 64) due to ineffective cross-conjugation associated with the honeycomb lattice. As shown in the zoom-in band structure (Supplementary Figs. 65 and 66) and partial charge distribution (Supplementary Fig. 67), **2DPAV-BTT-BP** and **2DPAV-BTT-P** present π-delocalized frontier orbitals and band dispersion intensities more than three times higher than those of the corresponding 2D PIs. UV–vis absorption spectra reveal an optical band gap reduction by approximately 0.05 and 0.2 eV for the above crystalline 2D PAVs compared with the amorphous 2D PAVs synthesized via the Knoevenagel reaction (labelled by Kn) and corresponding 2D PIs, respectively (see details in Fig. 6a and Supplementary Figs. 68 and 69).

To elucidate the charge transport properties of 2D PAVs associated with conjugation and crystallinity, we measured the time-resolved photoconductivity of crystalline and amorphous 2D PAVs as well as related 2D PIs by optical-pump terahertz probe spectroscopy. As shown in Fig. 6b, **2DPAV-BTT-BP** shows a photoconductivity intensity at least ten times higher than that of amorphous **2DPAV-BTT-BP(Kn)**, indicating that the intrinsic charge carrier mobility of crystalline 2D PAVs is at least one order of magnitude higher than their amorphous counterparts (a similar trend is observed for **2DPAV-BTT-P** system). Moreover, frequency-resolved photoconductivity measurements reveal an intrinsic charge carrier mobility of approximately 10 cm^2^ V^−^^1^ s^−^^1^ for **2DPAV-BTT-BP**, which is nearly ten times that of **2DPI-BTT-BP** (the charge carrier scattering time is 56 fs for the former and 17 fs for the latter; see more details in Figs. 5f and 6c and Supplementary Figs. 70 and 71). Characterizing pellet samples using the four-probe method further demonstrates an electrical conductivity of **2DPAV-BTT-BP** three times greater than that of **2DPAV-BTT-BP(Kn)** (Fig. 6d). These results underscore the crucial role of π-conjugation and crystallinity in enhancing the charge transport properties of 2D c-COFs.

## Discussion

In conclusion, we have developed a Mannich-elimination synthetic strategy that efficiently forms C=C bonds, as demonstrated through multiple model reactions and theoretical calculations. This approach is general to the synthesis of highly crystalline 2D PAVs in diverse lattice structures, such as honeycomb, square and kagome, derived from the 2D PI precursors. The specific surface areas of these 2D PAVs can be more than 100 times greater than those produced by the traditional Knoevenagel reaction. We resolve the honeycomb crystal structure of a single-crystalline 2D PAV at the molecular level. Our results further reveal that the crystallinity and π-conjugation of 2D PAVs significantly impact the charge carrier transport properties of 2D PAVs. This work not only deepens our understanding of 2D PAVs but also opens new avenues for crafting well-defined, conjugated, highly porous crystalline polymer materials for robust applications.

## Methods

### Synthesis of model compound 3 via Mannich-elimination reaction

A mixture of **1** (50 mg, 275.88 µmol), **2** (57.36 mg, 331.06 µmol), Cs_2_CO_3_ (179.77 mg, 551.76 µmol), DMAc (3 ml) and water (0.3 ml) was added to a Schlenk tube. The Schlenk tube was sealed under an inert environment and heated at 120 °C for 8 h. After the reaction, 50 ml water was added into the reaction mixture, which was then extracted with dichloromethane. The crude product was purified by silica gel chromatography using hexane/ethyl acetate = 97/3 as eluent to give **3** as a light-yellow solid in 98% yield (70 mg).

### Synthesis of 2DPAV-BTT-BP

A glass ampoule was charged with **2DPI-BTT-BP** (7 mg), 2,2′-([1,1′-biphenyl]-4,4′-diyl)diacetonitrile (14 mg, 2 equivalents per C=N bond), Cs_2_CO_3_ (50 mg, 4 equivalents per C=N bond) and a mixture of DMAc/H_2_O (0.7/0.3 ml). Afterwards, the mixture was sonicated for 10 min at room temperature, and the ampoule was degassed by three freeze–pump–thaw cycles, sealed under vacuum and heated at 130 °C for 6 days. After cooling to room temperature, the resulting powders were filtered and sequentially washed with dimethylformamide, H_2_O, ethanol and acetone. After Soxhlet extraction with tetrahydrofuran for 18 h, the sample was collected and dried under vacuum at 100 °C overnight, yielding **2DPAV-BTT-BP** (7.4 mg) as a light-orange solid with a 96% yield. Following the same conditions, 2,2′-([2,2′-bipyridine]-5,5′-diyl)diacetonitrile (14 mg) or 2,2′-([2,2′-bithiophene]-5,5′-diyl)diacetonitrile (14 mg) was used to synthesize **2DPAV-BTT-BPY** (7.4 mg, red solid) and **2DPAV-BTT-BT** (7 mg, dark-brown solid) in 95% and 91% yield, respectively.

### Synthesis of single-crystalline 2DPAV-DMP-TPB

A glass ampoule was charged with single-crystalline **2DPI-DMP-TPB** (5 mg), 2,2′-(5′-(4-(cyanomethyl)phenyl)-[1,1′:3′,1′′-terphenyl]-4,4′′-diyl)diacetonitrile (10 mg, 1.2 equivalents per C=N bond), Cs_2_CO_3_ (50 mg, 8 equivalents per C=N bond) and a mixture of DMAc/H_2_O (0.5/0.1 ml). Afterwards, the mixture was sonicated for 10 min at room temperature, and the ampoule was degassed by three freeze–pump–thaw cycles, sealed under vacuum and heated at 130 °C for 12 days. After cooling to room temperature, the resulting powders were filtered and sequentially washed with dimethylformamide, H_2_O, ethanol and acetone. After Soxhlet extraction with acetone for 18 h, the sample was collected and dried under vacuum at 100 °C overnight to give **2DPAV-DMP-TPB** (2.6 mg) as a dark-orange solid in 48% yield.

### HR-TEM imaging and image simulation

Experiments were performed on an image-side spherical aberration-corrected FEI Titan 80-300 operated at 300 kV. The FEI Titan is equipped with a CEOS hexapole spherical aberration coefficient (*C*_s_) corrector, capable of correcting geometrical axial aberrations up to the third order. The images were acquired using a Gatan UltraScan1000 CCD camera. HR-TEM image simulations were performed with the abTEM package^49^ using the Quantum Expresso plane-wave DFT code by using the GGA-PBE relaxed multilayer **2DPAV-DMP-TPB**.

## Online content

Any methods, additional references, Nature Portfolio reporting summaries, source data, extended data, supplementary information, acknowledgements, peer review information; details of author contributions and competing interests; and statements of data and code availability are available at 10.1038/s41557-025-02048-8.

## Supplementary information

Supplementary Information Supplementary Figs. 1–72, Discussion and Tables 1 and 2.

Supplementary Data 1 Coordinates for the optimized structures.

## Source data

Source Data Fig. 1 In situ NMR analysis of model reaction performed in DMAc (Fig. 1c), time-dependent solid-state ^13^C CP NMR (Fig. 1e) and FT-IR spectra (Fig. 1f) during the synthesis of **2DPAV-BTT-P(F)** from **2DPI-BTT-P(F)**.

Source Data Fig. 3 PXRD pattern (Fig. 3a,d), N_2_ physisorption isotherms (Fig. 3b,e) and pore size distribution (Fig. 3c,f).

Source Data Fig. 5 Experimental PXRD pattern, Rietveld-refined profile and their difference plots (Fig. 5d).

Source Data Fig. 6 UV–vis absorption data (Fig. 6a), time-resolved terahertz photoconductivity data (Fig. 6b), frequency-resolved terahertz photoconductivity data (Fig. 6c) and comparison of charge carrier mobilities and electrical conductivities (Fig. 6d).

## Data Availability

All data supporting the findings of this study are available in the Article or its Supplementary Information and are also available from the corresponding authors upon reasonable request. Source data are provided with this paper. These data are also available via Figshare at 10.6084/m9.figshare.30571898.v2 (ref. ^50^).

## References

[1] Ascherl, L. et al. Molecular docking sites designed for the generation of highly crystalline covalent organic frameworks. *Nat. Chem.* **8**, 310–316 (2016).

[2] Zhang, W. et al. Reconstructed covalent organic frameworks. *Nature* **604**, 72–79 (2022). 35388196 10.1038/s41586-022-04443-4 PMC8986529

[3] Xing, G. et al. Nonplanar rhombus and kagome 2D covalent organic frameworks from distorted aromatics for electrical conduction. *J. Am. Chem. Soc.* **144**, 5042–5050 (2022). 35189061 10.1021/jacs.1c13534

[4] Koner, K. & Banerjee, R. Porous polycyclic aromatic heterocycles via metal-free annulative π-extension. *Nat. Synth.* **3**, 1266–1274 (2024).

[5] Jin, Y., Hu, Y. & Zhang, W. Tessellated multiporous two-dimensional covalent organic frameworks. *Nat. Rev. Chem.* **1**, 0056 (2017).

[6] Guan, X. et al. Chemically stable polyarylether-based covalent organic frameworks. *Nat. Chem.* **11**, 587–594 (2019). 30962609 10.1038/s41557-019-0238-5

[7] Zhao, S. et al. Hydrophilicity gradient in covalent organic frameworks for membrane distillation. *Nat. Mater.* **20**, 1551–1558 (2021). 34294883 10.1038/s41563-021-01052-w

[8] Wang, M. et al. Exceptionally high charge mobility in phthalocyanine-based poly(benzimidazobenzophenanthroline)-ladder-type two-dimensional conjugated polymers. *Nat. Mater.* **22**, 880–887 (2023). 37337069 10.1038/s41563-023-01581-6 PMC10313522

[9] Galeotti, G. et al. Synthesis of mesoscale ordered two-dimensional π-conjugated polymers with semiconducting properties. *Nat. Mater.* **19**, 874–880 (2020). 32424372 10.1038/s41563-020-0682-z

[10] Wang, Z., Wang, M., Heine, T. & Feng, X. Electronic and quantum properties of organic two-dimensional crystals. *Nat. Rev. Mater.* **10**, 147–166 (2025).

[11] Wang, M. et al. Unveiling electronic properties in metal–phthalocyanine-based pyrazine-linked conjugated two-dimensional covalent organic frameworks. *J. Am. Chem. Soc.* **141**, 16810–16816 (2019). 31557002 10.1021/jacs.9b07644

[12] Zhao, W. et al. Using sound to synthesize covalent organic frameworks in water. *Nat. Synth.* **1**, 87–95 (2022).

[13] Zhan, G. et al. Observing polymerization in 2D dynamic covalent polymers. *Nature* **603**, 835–840 (2022). 35355001 10.1038/s41586-022-04409-6

[14] Jeon, J.-P. et al. Benzotrithiophene-based covalent organic framework photocatalysts with controlled conjugation of building blocks for charge stabilization. *Angew. Chem. Int. Ed.* **62**, e202217416 (2023). 10.1002/anie.202217416 36545845

[15] Zhou, Z. et al. Conformational chirality of single-crystal covalent organic frameworks. *J. Am. Chem. Soc.* **146**, 34064–34069 (2024). 39611551 10.1021/jacs.4c13394

[16] Auras, F. et al. Dynamic two-dimensional covalent organic frameworks. *Nat. Chem.* **16**, 1373–1380 (2024). 38702406 10.1038/s41557-024-01527-8

[17] Gruber, C. G., Frey, L., Guntermann, R., Medina, D. D. & Cortés, E. Early stages of covalent organic framework formation imaged in operando. *Nature* **630**, 872–877 (2024). 38839960 10.1038/s41586-024-07483-0 PMC11208157

[18] Cusin, L., Peng, H., Ciesielski, A. & Samorì, P. Chemical conversion and locking of the imine linkage: enhancing the functionality of covalent organic frameworks. *Angew. Chem. Int. Ed.* **60**, 14236–14250 (2021). 10.1002/anie.202016667 33491860

[19] Evans, A. M. et al. Two-dimensional polymers and polymerizations. *Chem. Rev.* **122**, 442–564 (2022). 34852192 10.1021/acs.chemrev.0c01184

[20] Zhuang, X. et al. A two-dimensional conjugated polymer framework with fully *sp*^2^-bonded carbon skeleton. *Polym. Chem.* **7**, 4176–4181 (2016).

[21] Jin, E. et al. Two-dimensional *sp*^2^ carbon–conjugated covalent organic frameworks. *Science* **357**, 673–676 (2017). 28818940 10.1126/science.aan0202

[22] Liu, Y. et al. A thiophene backbone enables two-dimensional poly(arylene vinylene)s with high charge carrier mobility. *Angew. Chem. Int. Ed.* **62**, e202305978 (2023). 10.1002/anie.202305978 37271733

[23] Liu, R. et al. Linkage-engineered donor–acceptor covalent organic frameworks for optimal photosynthesis of hydrogen peroxide from water and air. *Nat. Catal.* **7**, 195–206 (2024).

[24] Li, S. et al. Direct construction of isomeric benzobisoxazole–vinylene-linked covalent organic frameworks with distinct photocatalytic properties. *J. Am. Chem. Soc.* **144**, 13953–13960 (2022). 35877552 10.1021/jacs.2c06042

[25] Zhou, Z. et al. Carbon dioxide capture from open air using covalent organic frameworks. *Nature* **635**, 96–101 (2024). 39443804 10.1038/s41586-024-08080-x

[26] Wang, Y. et al. Facile construction of fully *sp*^2^-carbon conjugated two-dimensional covalent organic frameworks containing benzobisthiazole units. *Nat. Commun.* **13**, 100 (2022). 35013158 10.1038/s41467-021-27573-1 PMC8748616

[27] Bi, S. et al. Heteroatom-embedded approach to vinylene-linked covalent organic frameworks with isoelectronic structures for photoredox catalysis. *Angew. Chem. Int. Ed.* **61**, e202111627 (2022). 10.1002/anie.202111627 34813141

[28] Liu, M. et al. Two-dimensional covalent organic framework films prepared on various substrates through vapor induced conversion. *Nat. Commun.* **13**, 1411 (2022). 35301302 10.1038/s41467-022-29050-9 PMC8931112

[29] Xu, S. et al. Thiophene-bridged donor–acceptor *sp*^2^-carbon-linked 2D conjugated polymers as photocathodes for water reduction. *Adv. Mater.* **33**, 2006274 (2021). 33191503 10.1002/adma.202006274 PMC11468691

[30] Liu, Y. et al. Enhanced hydrogen peroxide photosynthesis in covalent organic frameworks through induced asymmetric electron distribution. *Nat. Synth.* **4**, 134–141 (2025).

[31] Zhang, Y., Guan, X., Meng, Z. & Jiang, H.-L. Supramolecularly built local electric field microenvironment around cobalt phthalocyanine in covalent organic frameworks for enhanced photocatalysis. *J. Am. Chem. Soc.* **147**, 3776–3785 (2025). 39817693 10.1021/jacs.4c16538

[32] Wang, Z. et al. Green synthesis of olefin-linked covalent organic frameworks for hydrogen fuel cell applications. *Nat. Commun.* **12**, 1982 (2021). 33790298 10.1038/s41467-021-22288-9 PMC8012354

[33] Jadhav, T. et al. 2D poly(arylene vinylene) covalent organic frameworks via aldol condensation of trimethyltriazine. *Angew. Chem. Int. Ed.* **58**, 13753–13757 (2019). 10.1002/anie.201906976 31359568

[34] Lyu, H., Diercks, C. S., Zhu, C. & Yaghi, O. M. Porous crystalline olefin-linked covalent organic frameworks. *J. Am. Chem. Soc.* **141**, 6848–6852 (2019). 31017397 10.1021/jacs.9b02848

[35] Acharjya, A., Pachfule, P., Roeser, J., Schmitt, F.-J. & Thomas, A. Vinylene-linked covalent organic frameworks by base-catalyzed aldol condensation. *Angew. Chem. Int. Ed.* **58**, 14865–14870 (2019). 10.1002/anie.201905886 PMC6851556 31340082

[36] Pastoetter, D. L. et al. Synthesis of vinylene-linked two-dimensional conjugated polymers via the Horner–Wadsworth–Emmons reaction. *Angew. Chem. Int. Ed.* **59**, 23620–23625 (2020). 10.1002/anie.202010398 PMC7814668 32959467

[37] Liu, Y. et al. Vinylene-linked 2D conjugated covalent organic frameworks by Wittig reactions. *Angew. Chem. Int. Ed.* **61**, e202209762 (2022). 10.1002/anie.202209762 PMC10098612 36161682

[38] Niu, C.-P., Zhang, C.-R., Liu, X., Liang, R.-P. & Qiu, J.-D. Synthesis of propenone-linked covalent organic frameworks via Claisen–Schmidt reaction for photocatalytic removal of uranium. *Nat. Commun.* **14**, 4420 (2023). 37479725 10.1038/s41467-023-40169-1 PMC10361971

[39] Ma, T. et al. Single-crystal X-ray diffraction structures of covalent organic frameworks. *Science* **361**, 48–52 (2018). 29976818 10.1126/science.aat7679

[40] Banerjee, T. et al. Sub-stoichiometric 2D covalent organic frameworks from tri- and tetratopic linkers. *Nat. Commun.* **10**, 2689 (2019). 31217421 10.1038/s41467-019-10574-6 PMC6584614

[41] Peng, L. et al. Ultra-fast single-crystal polymerization of large-sized covalent organic frameworks. *Nat. Commun.* **12**, 5077 (2021). 34426571 10.1038/s41467-021-24842-x PMC8382702

[42] Kang, C. et al. Growing single crystals of two-dimensional covalent organic frameworks enabled by intermediate tracing study. *Nat. Commun.* **13**, 1370 (2022). 35296677 10.1038/s41467-022-29086-x PMC8927472

[43] Arrayás, R. G. & Carretero, J. C. Catalytic asymmetric direct Mannich reaction: a powerful tool for the synthesis of α,β-diamino acids. *Chem. Soc. Rev.* **38**, 1940–1948 (2009). 19551174 10.1039/b820303b

[44] Selvi, T. & Velmathi, S. Indium(III) triflate-catalyzed reactions of aza-Michael adducts of chalcones with aromatic amines: retro-Michael addition versus quinoline formation. *J. Org. Chem.* **83**, 4087–4091 (2018). 29498283 10.1021/acs.joc.7b03151

[45] Poisson, T., Gembus, V., Oudeyer, S., Marsais, F. & Levacher, V. Product-catalyzed addition of alkyl nitriles to unactivated imines promoted by sodium aryloxide/ethyl(trimethylsilyl)acetate (ETSA) combination. *J. Org. Chem.* **74**, 3516–3519 (2009). 19358522 10.1021/jo802763b

[46] Xu, H. et al. Proton conduction in crystalline and porous covalent organic frameworks. *Nat. Mater.* **15**, 722–726 (2016). 27043780 10.1038/nmat4611

[47] Xu, H. et al. Stable, crystalline, porous, covalent organic frameworks as a platform for chiral organocatalysts. *Nat. Chem.* **7**, 905–912 (2015). 26492011 10.1038/nchem.2352

[48] Natraj, A. et al. Single-crystalline imine-linked two-dimensional covalent organic frameworks separate benzene and cyclohexane efficiently. *J. Am. Chem. Soc.* **144**, 19813–19824 (2022). 36265086 10.1021/jacs.2c07166

[49] Madsen, J. & Susi, T. The abTEM code: transmission electron microscopy from first principles. *Open Res. Eur.* **1**, 24 (2021). 37645137 10.12688/openreseurope.13015.2 PMC10446032

[50] Ghouse, S. et al. Towards Single-Crystalline Two-Dimensional Poly(arylene vinylene) Covalent Organic Frameworks. *Figshare*10.6084/m9.figshare.30571898.v2 (2025). 10.1038/s41557-025-02048-8 PMC13149328 41559418

