# Supplementary material for: Towards single-crystalline two-dimensional poly(arylene vinylene) covalent organic frameworks
Source: Nat Chem. 2026 Jan 20;18(5):853–62. doi: 10.1038/s41557-025-02048-8 (PMC13149328; doi:10.1038/s41557-025-02048-8)
Supplement: Supplementary file 1 — Supplementary Figs. 1–72, Discussion and Tables 1 and 2. [file 41557_2025_2048_MOESM1_ESM.pdf]

nature chemistry

Supplementary information https://doi.org/10.1038/s41557-025-02048-8

Towards single-crystalline two-dimensional poly(arylene vinylene) covalent organic frameworks

In the format provided by the authors and unedited

Table of Contents

Section A. Supplementary Methods………………………………….……………..S2

Section B. Materials and synthetic procedure………………………………...S6

Section C. Supplementary Figures…………………………………………….S19

Section D. Supplementary Tables……………………………………………...S65

Section E. Supplementary references…………………………………………S67

Section A. Supplementary Methods

Instrumentation and characterization

Nuclear magnetic resonance (NMR) spectroscopy was carried out on Bruker AV-II 300 spectrometer operating at 300.1 MHz for ^{1}H NMR. Chemical shifts are given in ppm relative to tetramethylsilane (TMS). **Solid-state NMR** spectrum were recorded on BRUKER Ascend 300 MHz spectrometer using a commercial 2.5 mm MAS NMR probe and operating at a resonance frequency of 75.48 MHz for ^{13}C and 300.13 MHz for ^{1}H. The MAS frequency was 15 kHz. Cross polarization (CP) and SPINAL 1H decoupling were used during signal acquisition. The ^{1}H and ^{13}C chemical shift were referenced relative to TMS using adamantane as secondary standard. The signals were assigned using the program ACD/Labs (ACD/Labs 2019, Advanced Chemistry Development, Inc., Toronto, Canada) As external standard, adamantane was used. **Fourier transform infrared (FT-IR)** spectroscopy was performed on a Bruker Optics ALPHA-E spectrometer with a universal Zn-Se ATR (attenuated total reflection) accessory in the 400−4000 cm^{-1}. **MALDI-TOF mass spectrometry** was recorded on a Bruker Autoflex Speed MALDI TOF MS (Bruker Daltonics, Bremen, Germany) with trans-2-[3-(4-tert-Butylphenyl)-2-methyl-2-propenylidene] malononitrile (DCTB) as the matrix. The instrument is equipped with an Agilent Series 1200 HPLC binary pump, and Autosampler, using Mass Hunter software. **Ultraviolet-Visible (UV/vis) absorption** was measured on an Agilent Cary 5000 UV-VIS-NIR spectrophotometer by using 10 mm optical-path quartz cell at room temperature. **Raman** spectrum were acquired using a RFT-6000-JASCO spectrometer equipped with a continuous-wave Nd:YAG laser working at λ=1064 nm and a InGaAs detector. Raman scattering radiation was collected in a back-scattering configuration with a standard spectruml resolution of 4 cm^{-1}. The laser power was maintained below 40 mW in all cases, and 1000 scans were averaged for each spectrum to ensure an optimal signal-to-noise ratio. **Powder X-ray diffraction (PXRD)** patterns were obtained on an X-ray diffractometer (Dectris Mythen 1K Strip Detector, Stoe Stadi-P) using Cu-Kα radiation (λ = 0.15418 nm) at 40 kV and 40 mA at room temperature. The as-obtained powder samples were measured in transmission geometry. Temperature-variable powdered X-ray diffraction patterns were recorded with a linear wire position sensitive detector. **Scanning electron microscopy (SEM)** images were recorded on a Zeiss Gemini S4 500. **Nitrogen physisorption (gas adsorption-desorption) isotherm measurements** were conducted on Micromeritics 3Flex automated, three-station, surface

area and porosity analyzer. The measurements were performed at 77 K, maintained by a liquid nitrogen bath, with pressures ranging from 0 to 760 Torr. The isotherms were collected in the relative pressure (P/Po) range 0 to 0.99 atm at 77 K. The Brunauer-Emmett-Teller (BET) method was utilized to calculate the specific surface areas within the relative pressure (P/Po) range of 0.01 to 0.2. And non-local density functional theory (NLDFT) model was used to determine the pore sizes and volumes. The total pore volume was calculated at P/Po=0.9. Prior to surface area analysis the samples were activated at 120 °C for 24 h. **High-resolution transmission electron microscopy (TEM)** experiments were performed on an image-side spherical aberration-corrected FEI Titan 80-300 operated at 300 kV. The FEI Titan is equipped with a CEOS hexapole C_s corrector, capable of correcting geometrical axial aberrations up to the 3rd-order. on the images were acquired using a Gatan UltraScan1000 CCD camera. Image simulation. HR-TEM image simulations were performed with the abTEM package[1] using the Quantum Expresso plane-wave DFT code by using the GGA-PBE relaxed multilayer **2DPAV-DMP-TPB**. The **continuous rotation electron diffraction (cRED)** data was collected on JEOL 2100Plus transmission electron microscope (200 kV) equipped with DiffPro software suit and Axion hybrid pixel detector. During the data collection the sample was cooled to −175 °C by using CryoHolder CHO1. The exposure time is 1s and the rotation speed is 1°/s.

Time-resolved THz spectroscopy. The measurements were performed using an optical pump—THz probe setup driven by a titanium:sapphire laser amplifier system generating ~40 fs width laser pulses with a central wavelength of 800 nm, a pump fluence of 300 $\mu J/cm2$, and a repetition rate of 1 kHz. The ~200-micron thick sample (O.D. at 400 nm = 2.3) was sandwiched between fused silica substrates and measured in transmission under a nitrogen environment. The phase-sensitive detection of the THz pulse allows the evaluation of the complex conductivity of the photo-excited sample. The frequency-

$$\sigma_{DS}(\omega) = \frac{\epsilon_0 \omega_p^2 \tau_{DS}}{1 - i\omega\tau_{DS}} (1 \frac{C}{1 - i\omega\tau_{DS}})$$

resolved complex photoconductivity spectrum of the sample were fitted by using the Drude-Smith model:

The Drude-Smith model is an extension of the classical Drude model that incorporates several parameters to describe the electrical conductivity of materials, particularly in the terahertz frequency range. The key parameters in the Drude-Smith model are the momentum relaxation time (τ_{DS}): This parameter represents the average time between

collisions of charge carriers. It is also referred to as the scattering time and is typically measured in femtoseconds (fs); plasma frequency (ω_p), related to the carrier effective mass, and the square of which is proportional to the number of charge carriers per unit volume in the material; persistence of velocity, also known as the *c* parameter, this coefficient describes the degree of backscattering or localization effect for each electronic scattering event. It ranges from 0 to -1, with values closer to -1 indicating stronger carrier localization. These parameters allow the Drude-Smith model to account for carrier confinement on the mesoscopic scale and provide a more accurate representation of conductivity in systems where free charges are accumulated by localized scatterers.

Conductivity 4-probe conductivity at room temperature of pellet samples (2x1x5 mm, pressed at 1.25 t) was recorded using a commercial Signatone Pro4 resistivity measurement system. For each sample a minimum of 5 repetitions were performed.

Theoretical calculations of molecular fragments. Dimeric systems, where two benzo[1,2-b:3,4-b':5,6-b"]trithiophene units are linked by different π-spacers (see Supplementary Fig. 25), were considered as theoretical models for the 2D conjugated polymers under study. These models provide a reasonable starting point for constructing the full periodic systems, as quantum chemical calculations of molecular fragments have been demonstrated to provide valuable insights into the molecular, electronic structure and charge-transport properties of 2D conjugated polymers.[2–10] The dimeric models were optimized using Density Functional Theory (DFT) with the hybrid, generalized gradient approximation (GGA) functional PBE0[11] and the 6-31G**[12,13] basis set, as implemented in the GAUSSIAN16 program.[14] A preliminary study of dihedral potential energy hypersurfaces was performed to deeper insight into the conformational preferences of these systems (Supplementary Fig. 26). The relaxed potential energy hypersurfaces were computed by constraining the selected dihedral angles (by step of 10°) while allowing all other degrees of freedom to relax to their potentially energy minima. The minimum energy conformers were then used to model the dimeric structures corresponding to the respective 2D polymers. Harmonic frequencies calculations were performed on the optimized structures, confirming the absence of imaginary frequencies. Raman intensities were calculated by using an adjustment of the theoretical force fields in which the frequencies are scaled down by a factor of 0.94 to disentangle experimental misassignments. The theoretical spectrum were obtained by convolving the scaled frequencies and the Raman scattering activities with Gaussian functions (8 cm^{-1} width at

the half-height). Molecular orbitals distribution and vibrational eigenvectors were plotted using the ChemCraft 1.8 molecular modelling software.[15]

Theoretical calculations of periodic systems. The previously optimized building blocks were considered reasonable starting point geometries towards the assembling of the whole bidimensional polymeric systems. Once the 2D porous polymers were constructed, they were fully optimized (simultaneous lattice/cell and structure optimizations) with the Quantum Expresso plane-wave DFT code[16] by using the GGA-PBE[17] functional and ultra-soft pseudopotentials.[18,19] Furthermore, we use the Grimme DFT-D3 semiempirical efficient vdW correction to include dispersion forces and energies.[20] The Brillouin zones were sampled by means of optimal Monkhorst-Pack grids,[21] with the one-electron wave-functions being expanded in a basis of plane-waves with a kinetic energy cutoff of 41 Ry and 260 Ry for electronic density. The energy cutoff values have been tested to achieve sufficient accuracy to guarantee a full convergence in total energy and electronic density. Relaxations of cell shape and size have been double-checked with two different algorithms: a damped dynamics, and a Broyden–Fletcher–Goldfarb–Shanno-like relaxation.[22–24] These two approaches, tested thoroughly in the literature for different crystal bulk and molecular configurations, provide very similar and satisfactory results for cell-shape and lattice parameters. Topologies of valence and conduction bands at the k-point have been plotted using the VMD 1.9.3 program,[25] by using an isosurface value of 0.0003 e-/A. The same program has been used in order to represent the surface charge distributions of the resulting 2D polymers.

Section B. Materials and Synthetic Procedures

Materials

All solvents, reagents and chemicals were purchased from commercial suppliers, such as Sigma-Aldrich, TCI and abcr GmbH, and used without further purification unless otherwise specified. All the reactions were carried out under argon or vacuum using the standard vacuum-line and Schleck techniques. Column chromatography was performed using silica gel.

Synthesis of 2-(4-(tert-butyl)phenyl)-3-phenylacrylonitrile (3)

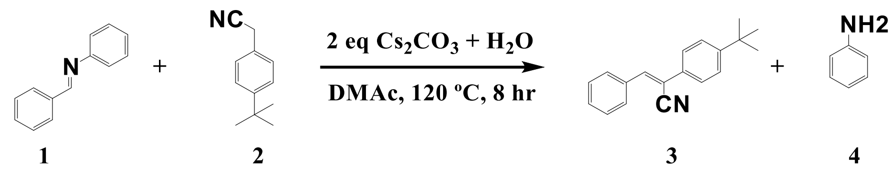

Supplementary Scheme 1. Synthesis of model compound **3** via Mannich-elimination reaction.

A mixture of **1** (50 mg, 275.88 µmol), **2** (57.36 mg, 331.06 µmol) and Cs_2CO_3 (179.77 mg, 551.76 µmol) was along with 0.3 mL of water and 3 mL of dimethylacetamide was added to a Schlenk tube. The Schlenk tube was sealed under an inert environment and heated to 120 °C for 8 h. After the reaction, to the crude solution 50 mL water was added and extracted with dichloromethane (3 x 50 mL) and the organic layers were combined, dried over Na_2SO_4, and filtered. The volatiles were removed in vacuo and the crude product was purified by silica gel chromatography. (PE/AcOEt = 97:3) to yield pale yellow solid (70 mg, 98% yield). ^{1}H NMR (300 MHz, DMSO) δ 8.00 (s, 1H), 7.94 (dd, *J* = 7.7, 1.5 Hz, 2H), 7.75 – 7.66 (m, 2H), 7.61 – 7.46 (m, 5H), 1.32 (s, 9H).

Synthesis of Imine-linked 2D COFs (2D PIs)

The 2D PIs were synthesized according to the previously reported procedures for **2DPI-TPB-P**[26], **2DPI-TPT-P**[27], **2DPI-TPPy-P**[28], **2DPI-HATN-P**[29], **and 2DPI-T-TPB**[30].

Synthesis of 2DPI-TPB-P

A glass ampoule was charged with 1,3,5–tris(4–formylphenyl)benzene **TPB** 68 mg, 1,4 phenylene diamine **PDA** 13 mg and mesitylene/ethyl alcohol (1/1 by vol, 4ml). The ampoule was sonicated for 3 min at room temperature, and 0.1 mL of glacial acetic acid was then added to the solution. The reaction mixture was degassed via three freeze-pump-thaw cycles, sealed under vacuum, and heated at 120℃ for 3 days. After cooling to room temperature, the resulting powders were filtered and sequentially washed with dimethylformamide, H_2O, ethanol and acetone. After Soxhlet extraction with tetrahydrofuran for 10 h, the sample was collected and dried under vacuum at 100℃ overnight, yellow crystallites in 91 % isolated yield were obtained and characterized via PXRD, FT-IR, and Raman spectroscopy, as shown in Supplementary Figs. 12, 14 and 16.

Synthesis of 2DPI-TPT-P

A glass ampoule was charged with 8.1 mg **PDA**, 19.7 mg 2,4,6-tris(4-formylphenyl)-1,3,5- triazine **TPT**, 1 mL mesitylene and 1 mL1,4-dioxane. The ampoule was sonicated for 3 min at room temperature, and 0.1 mL of glacial acetic acid was then added to the solution. The reaction mixture was degassed via three freeze-pump-thaw cycles, sealed under vacuum, and heated at 120℃ for 3 days. After cooling to room temperature, the resulting powders were filtered and sequentially washed with dimethylformamide, H_2O, ethanol and acetone. After Soxhlet extraction with tetrahydrofuran for 10 h, the sample was collected and dried under vacuum at 100℃ overnight, yellow powder in 85 % isolated yield were obtained and characterized via PXRD, FT-IR, and ^{13}C CP NMR spectroscopy, as shown in Supplementary Fig. 47.

Synthesis of 2DPI-TPPy-P

A glass ampoule was charged with a mixture of TPPy 15.00 mg and PDA 5.19 mg in 1,4-dioxane/3 M HAc (10/1 by vol.; 1.1 mL). The ampoule was sonicated for 3 min at room temperature, and 0.1 mL of glacial acetic acid was then added to the solution. The reaction mixture was degassed via three freeze-pump-thaw cycles, sealed under vacuum, and heated at 90℃ for 3 days. After cooling to room temperature, the resulting powders were filtered and sequentially washed with dimethylformamide, H_2O, ethanol and acetone. After Soxhlet extraction with tetrahydrofuran for 10 h, the sample was collected and dried under vacuum at 100℃ overnight, yellow powder in 92 % isolated yield were obtained

and characterized via PXRD, FT-IR, and ^{13}C CP NMR spectroscopy, as shown in Supplementary Fig. 48.

Synthesis of 2DPI-HATN-P

A glass ampoule was charged with PDA, 4.8 mg, and HATN 15 mg and were added into a mixture of dimethylacetamide (DMAc, 0.5 mL), mesitylene (Mes, 0.5 mL) and acetic acid (aq. 6M. 0.1 mL). The ampoule was sonicated for 3 min at room temperature, and 0.1 mL of glacial acetic acid was then added to the solution. The reaction mixture was degassed via three freeze-pump-thaw cycles, sealed under vacuum, and heated at 90℃ for 3 days. After cooling to room temperature, the resulting powders were filtered and sequentially washed with dimethylformamide, H_2O, ethanol and acetone. After Soxhlet extraction with tetrahydrofuran for 10 h, the sample was collected and dried under vacuum at 100℃ overnight, greenish yellow powder in 67 % isolated yield were obtained and characterized via PXRD, FT-IR and ^{13}C CP NMR spectroscopy, as shown in Supplementary Fig. 49.

Synthesis of 2DPI-P-TPB

A glass ampoule was charged with a mixture of **TPB** (28 mg, 0.08 mmol), Terepthaldehyde **TP** (16.1 mg, 0.120 mmol), and an aqueous acetic acid solution (6 M, 0.1 mL) in BuOH/o-DCB (0.5 mL/0.5 mL). The ampoule was sonicated for 3 min at room temperature, and 0.1 mL of glacial acetic acid was then added to the solution. The reaction mixture was degassed via three freeze-pump-thaw cycles, sealed under vacuum, and heated at 90℃ for 3 days. After cooling to room temperature, the resulting powders were filtered and sequentially washed with dimethylformamide, H_2O, ethanol and acetone. After Soxhlet extraction with tetrahydrofuran for 10 h, the sample was collected and dried under vacuum at 100℃ overnight, greenish yellow powder in 83 % isolated yield were obtained and characterized via PXRD, and FT-IR spectroscopy, as shown in Supplementary Fig. 50.

Synthesis of 2DPAV-TPB-P

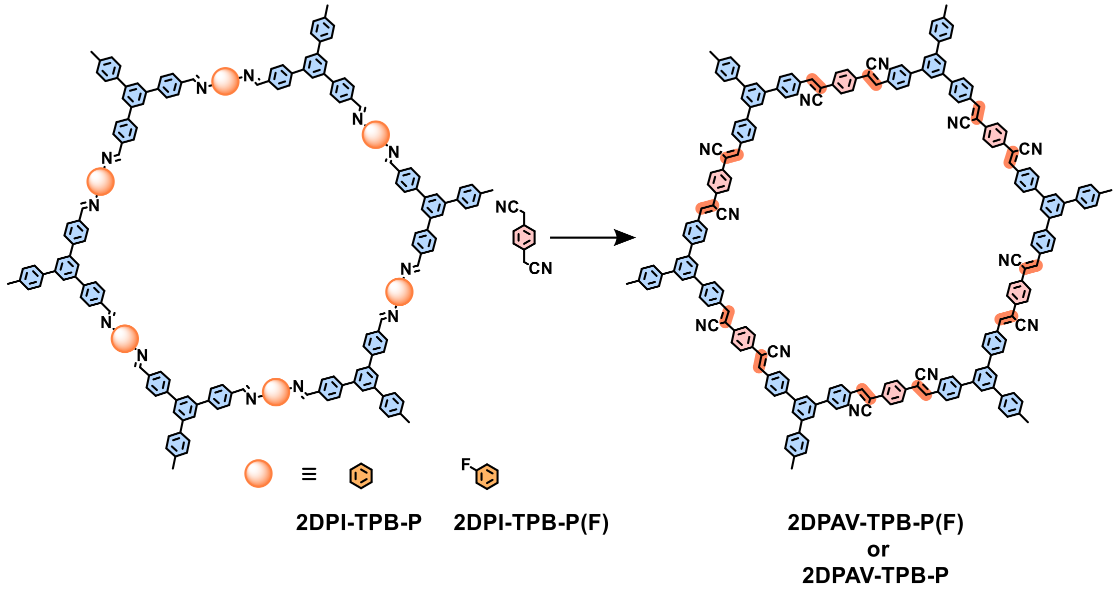

Supplementary Scheme 2. Ideal complete transformation of imine-linked 2D polymers (simple and fluorine-substituted) into 2DPAVs via the Mannich-elimination reaction.

A glass ampoule was charged with **2DPI-TPB-P** (7 mg), 2,2'-(1,4-phenylene)diacetonitrile (5 mg, 1 equivalent per C=N bond), Cs_2CO_3 (25 mg, 2 equivalents per C=N bond), and a solvent mixture of DMAc/H_2O (0.7/0.3 mL). The ampoule was degassed using three freeze-pump-thaw cycles, sealed under vacuum, and heated at 120℃ for 5 days. After cooling to room temperature, the resulting powder was filtered and subsequentially washed with dimethylformamide, H_2O, ethanol and acetone. The product was then subjected to Soxhlet extraction with tetrahydrofuran for 18 h. Finally, the sample was collected and dried under vacuum at 100℃ overnight, yielding **2DPAV-TPB-P** (90%) as yellow solid with a and characterized via PXRD, and FT-IR spectroscopy, as shown in Supplementary Fig. 12, 15 and 16.

Synthesis of 2DPAV-TPB-P(F)

A glass ampoule was charged with **2DPI-TPB-P(F)** (7 mg), 2,2'-(1,4-phenylene)diacetonitrile (4.8 mg, 1 equivalent per C=N bond) , Cs_2CO_3 (24 mg, 2 equivalents per C=N bond), and a solvent mixture of DMAc/H_2O (0.7/0.3 mL). The ampoule was degassed using three freeze-pump-thaw cycles, sealed under vacuum, and heated at 120℃ for 5 days. After cooling to room temperature, the resulting powder was filtered and subsequentially washed with dimethylformamide, H_2O, ethanol and acetone.

The product was then subjected to Soxhlet extraction with tetrahydrofuran for 18 h. Finally, the sample was collected and dried under vacuum at 100℃ overnight, yielding **2DPAV-TPB-P(F)** as yellow solid with a 90% yield and characterized via PXRD, FT-IR, Raman, ^{19}F NMR, and ^{13}C CP NMR spectroscopy, as shown in Supplementary Fig. 9,11, and 12.

General procedure to synthesize BTT-based 2D PIs

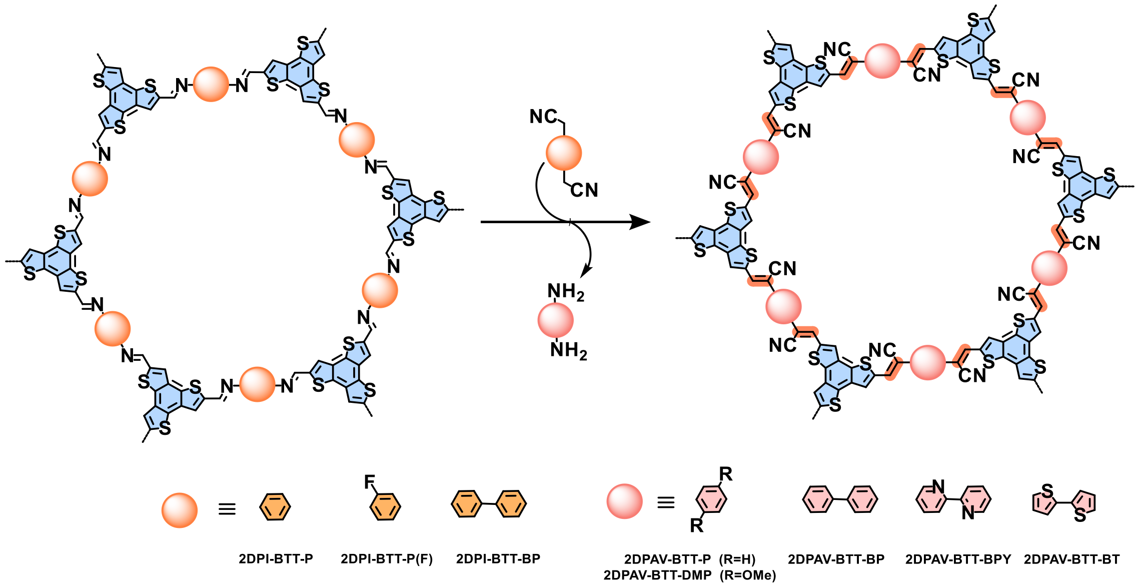

Supplementary Scheme 3. Ideal complete transformation of various BTT-based 2DPIs polymers (**2DPI-BTT-P**, **2DPI-BTT-P(F)** and **2DPI-BTT-BP**) into their respective 2DPAVs (**2DPAV-BTT-P**, **2DPAV-BTT-DMP, 2DPAV-BTT-BP**, **2DPAV-BTT-BPY** and **2DPAV-BTT-BT**) via Mannich-elimination reaction.

A glass ampoule was charged with benzo[1,2-b:3,4-b':5,6-b'']trithiophene-2,5,8-tricarbaldehyde (30 µmol), di-amine based monomer (45 µmol) and a mixture of DMAc/*o*-DCB (0.7/0.3 mL). The ampoule was sonicated for 3 min at room temperature, and 0.1 mL of glacial acetic acid was then added to the solution. The reaction mixture was degassed via three freeze-pump-thaw cycles, sealed under vacuum, and heated at 120℃ for 3 days. After cooling to room temperature, the resulting powders were filtered and sequentially washed with dimethylformamide, H_2O, ethanol and acetone. After Soxhlet extraction with tetrahydrofuran for 10 h, the sample was collected and dried under vacuum at 100℃ overnight, yielding the imine-linked 2D PIs with a yield exceeding 93%

and characterized via PXRD, FT-IR, Raman, ^{19}F NMR, and ^{13}C CP NMR spectroscopy, as shown in Supplementary Fig. 9,12,13,14, 26, 28, and 29.

Synthesis of 2DPAV-BTT-P

A glass ampoule was charged with **2DPI-BTT-P** (7 mg), 2,2'-(1,4-phenylene)diacetonitrile (12 mg, 2 equivalents per C=N bond), Cs_2CO_3 (50 mg, 4 equivalents per C=N bond), and a mixture of DMAc/H_2O (0.7/0.3 mL). . Afterwards, the mixture was sonicated for 10 minutes at room temperature and the ampoule was degassed by three freeze-pump-thaw cycles, sealed under vacuum, and heated at 130℃ for 6 days. After cooling to room temperature, the resulting powder was filtered and sequentially washed with dimethylformamide, H_2O, ethanol and acetone. After Soxhlet extraction with tetrahydrofuran for 18 h, the sample was collected and dried under vacuum at 100℃ overnight to give **2DPAV-BTT-P** (7.4 mg) as a dark red powder with a 95% yield and characterized via PXRD, FT-IR, and Raman spectroscopy, as shown in Supplementary Fig. 13,14, and 21.

Synthesis of 2DPAV-BTT-DMP

A glass ampoule was charged with **2DPI-BTT-P** (7 mg), 2,2'-(2,5-dimethoxy-1,4-phenylene)diacetonitrile(17 mg, 2 equivalents per C=N bond), Cs_2CO_3 (50 mg, 4 equivalents per C=N bond), and a mixture of DMAc/H_2O (0.7/0.3 mL). . Afterwards, the mixture was sonicated for 10 minutes at room temperature and the ampoule was degassed by three freeze-pump-thaw cycles, sealed under vacuum, and heated at 130℃ for 7 days. After cooling to room temperature, the resulting powder was filtered and sequentially washed with dimethylformamide, H_2O, ethanol and acetone. After Soxhlet extraction with tetrahydrofuran for 18 h, the sample was collected and dried under vacuum at 100℃ overnight to give **2DPAV-BTT-DMP** (9.1 mg) as a dark red powder with a 94% yield and characterized via PXRD, FT-IR, and ^{13}C CP NMR spectroscopy, as shown in Supplementary Fig. 25.

Synthesis of 2DPAV-BTT-P(F)

A glass ampoule was charged with **2DPI-BTT-P(F)** (7 mg), 2,2'-(1,4-phenylene)diacetonitrile (12 mg, 2 equivalents per C=N bond), Cs_2CO_3 (50 mg, 4 equivalents per C=N bond), and a mixture of DMAc/H_2O (0.7/0.3 mL). Afterwards, the

mixture was sonicated for 10 minutes at room temperature and the ampoule was degassed by three freeze-pump-thaw cycles, sealed under vacuum, and heated at 130℃ for 6 days. After cooling to room temperature, the resulting powder was filtered and sequentially washed with dimethylformamide, H_2O, ethanol and acetone. After Soxhlet extraction with tetrahydrofuran for 18 h, the sample was collected and dried under vacuum at 100℃ overnight to give **2DPAV-BTT-P(F)** (7.1 mg) as a dark red solid with a 95% yield and characterized via PXRD, FT-IR, ^{19}F NMR, and ^{13}C CP NMR spectroscopy, as shown in Supplementary Figs. 9, 10, 12 and 14.

Synthesis of 2DPAV-BTT-BP

A glass ampoule was charged with **2DPI-BTT-BP** (7 mg), 2,2'-([1,1'-biphenyl]-4,4'-diyl)diacetonitrile (14 mg, 2 equivalents per C=N bond), Cs_2CO_3 (50 mg, 4 equivalents per C=N bond), and a mixture of DMAc/H_2O (0.7/0.3 mL). Afterwards, the mixture was sonicated for 10 minutes at room temperature and the ampoule was degassed by three freeze-pump-thaw cycles, sealed under vacuum, and heated at 130℃ for 6 days. After cooling to room temperature, the resulting powder was filtered and sequentially washed with dimethylformamide, H_2O, ethanol and acetone. After Soxhlet extraction with tetrahydrofuran for 18 h, the sample was collected and dried under vacuum at 100℃ overnight, yielding **2DPAV-BTT-BP** (7.4 mg) as a light orange solid with a 96% yield and characterized via PXRD, FT-IR, Raman and ^{13}C CP NMR spectroscopy, as shown in Supplementary Figs. 28, 29, and 38.

Synthesis of 2DPAV-BTT-BPY

A glass ampoule was charged with **2DPI-BTT-BP** (7 mg), 2,2'-([2,2'-bipyridine]-5,5'-diyl)diacetonitrile (14 mg, 2 equivalents per C=N bond), Cs_2CO_3 (50 mg, 4 equivalents per C=N bond), and a mixture of DMAc/H_2O (0.7/0.3 mL). Afterwards, the mixture was sonicated for 10 minutes at room temperature and the ampoule was degassed by three freeze-pump-thaw cycles, sealed under vacuum, and heated at 130℃ for 6 days. After cooling to room temperature, the resulting powder was filtered and sequentially washed with dimethylformamide, H_2O, ethanol and acetone. After Soxhlet extraction with tetrahydrofuran for 18 h, the sample was collected and dried under vacuum at 100℃ overnight to give **2DPAV-BTT-BPY** (7.4 mg) as a red solid with a 95% yield and

characterized via PXRD, FT-IR, Raman and ^{13}C CP NMR spectroscopy, as shown in Supplementary Figs. 28, 29, and 38.

Synthesis of 2DPAV-BTT-BT

A glass ampoule was charged with **2DPI-BTT-BP** (7 mg), 2,2'-([2,2'-bithiophene]-5,5'-diyl)diacetonitrile (14 mg, 2 equivalents per C=N bond), Cs_2CO_3 (50 mg, 4 equivalents per C=N bond), and a mixture of DMAc/H_2O (0.7/0.3 mL). Afterwards, the mixture was sonicated for 10 minutes at room temperature and the ampoule was degassed using three freeze-pump-thaw cycles, sealed under vacuum, and heated at 120℃ for 6 days. After cooling to room temperature, the resulting powder was filtered and sequentially washed with dimethylformamide, H_2O, ethanol and acetone. After Soxhlet extraction with tetrahydrofuran for 18 h, the sample was collected and dried under vacuum at 100℃ overnight, yielding **2DPAV-BTT-BT** (7 mg) as a dark brown solid with a 91% yield characterized via PXRD, FT-IR, Raman, and ^{13}C CP NMR spectroscopy, as shown in Supplementary Figs. 28, 29, and 38.

Synthesis of 2DPAV-TPT-P

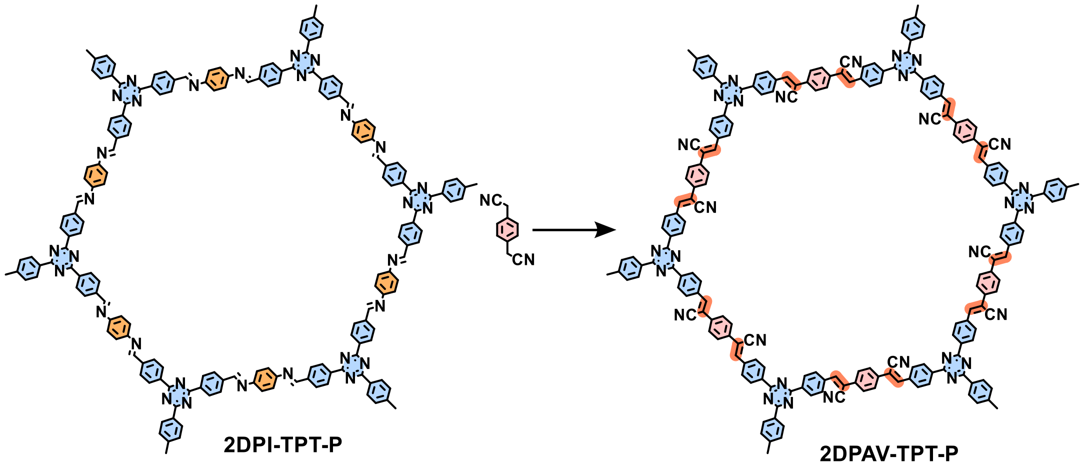

Supplementary Scheme 4. Ideal complete transformation of **2DPI-TPT-P** to **2DPAV-TPT-P** via Mannich-elimination reaction.

A glass ampoule was charged with **2DPI-TPT-P** (7 mg), 2,2'-(1,4-phenylene)diacetonitrile (5 mg, 1 equivalent per C=N bond), Cs_2CO_3 (25 mg, 2 equivalents per C=N bond), and a mixture of DMAc/H_2O (0.7/0.3 mL). The ampoule was degassed by three freeze-pump-thaw cycles, sealed under vacuum, and heated at 120℃ for 5 days. After cooling to room temperature, the resulting powder was filtered and

sequentially washed with dimethylformamide, H_2O, ethanol and acetone. After Soxhlet extraction with tetrahydrofuran for 18 h, the sample was collected and dried under vacuum at 100℃ overnight to give **2DPAV-TPT-P** (7 mg) as yellow solid with a 90% yield and characterized via PXRD, FT-IR and ^{13}C CP NMR spectroscopy, as shown in Supplementary Fig. 47.

Synthesis of 2DPAV-TPPy-P

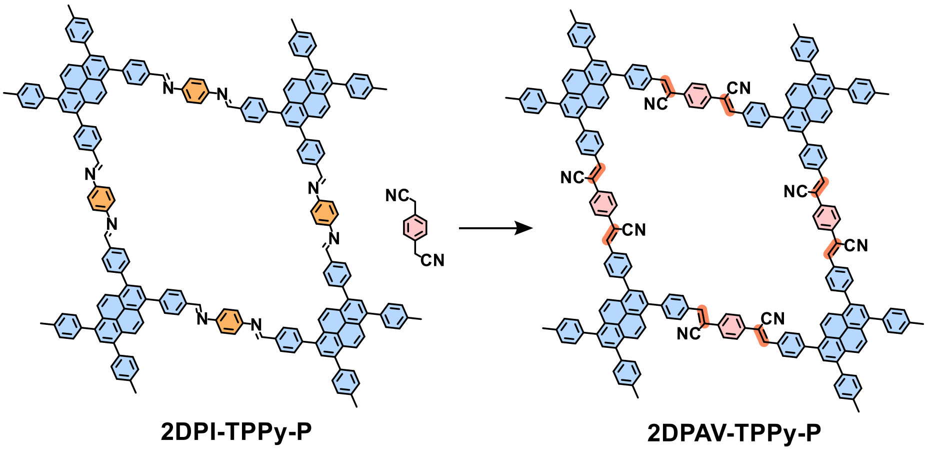

Supplementary Scheme 5. Ideal complete transformation of **2DPI-TPPy-P** to **2DPAV-TPPy-P** via Mannich-elimination reaction.

A glass ampoule was charged with **2DPI-TPPy-P** (7 mg), 2,2'-(1,4-phenylene)diacetonitrile (10 mg, 4 equivalents per C=N bond), Cs_2CO_3 (50 mg, 8 equivalents per C=N bond), and a mixture of DMAc/H_2O (0.7/0.2 mL). Afterwards, the mixture was sonicated for 10 minutes at room temperature and the ampoule was degassed by three freeze-pump-thaw cycles, sealed under vacuum, and heated at 130℃ for 8 days. After cooling to room temperature, the resulting powder was filtered and sequentially washed with dimethylformamide, H_2O, ethanol and acetone, After Soxhlet extraction with tetrahydrofuran for 18 h, the sample was collected and dried under vacuum at 100℃ overnight, yielding **2DPAV-TPPy-P** (7 mg) as bright orange solid with a 93% yield and characterized via PXRD, FT-IR and ^{13}C CP NMR spectroscopy, as shown in Supplementary Fig. 48.

Synthesis of 2DPAV-HATN-P

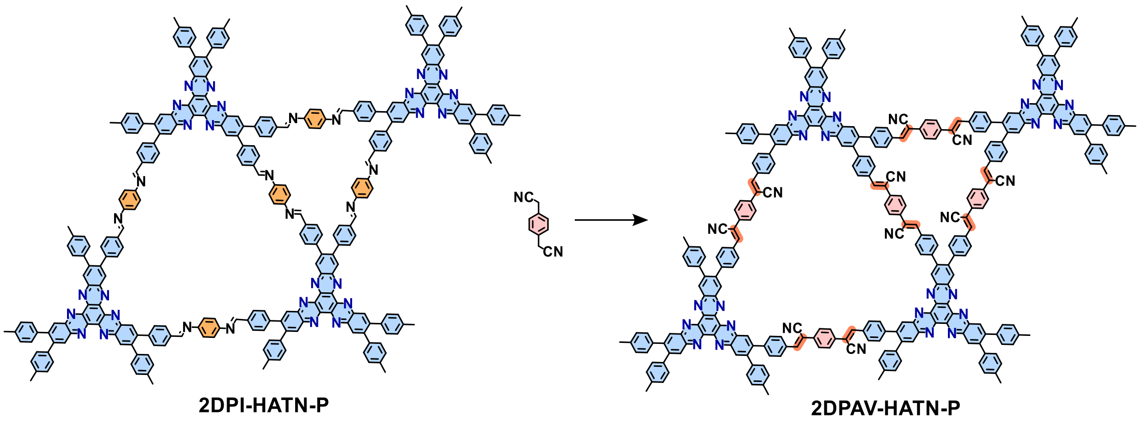

Supplementary Scheme 6. Ideal complete transformation of **2DPI-HATN-P** to **2DPAV-HATN-P** via Mannich-elimination reaction.

A glass ampoule was charged with **2DPI-HATN-P** (7 mg), 2,2'-(1,4-phenylene)diacetonitrile (10 mg, 4 equivalents per C=N bond), Cs_2CO_3 (50 mg, 8 equivalents per C=N bond), and a mixture of DMAc/H_2O (0.7/0.3 mL). Afterwards, the mixture was sonicated for 10 minutes at room temperature and the ampoule was degassed by three freeze-pump-thaw cycles, sealed under vacuum, and heated at 130℃ for 8 days. After cooling to room temperature, the resulting powder was filtered and sequentially washed with dimethylformamide, H_2O, ethanol and acetone. After Soxhlet extraction with tetrahydrofuran for 18 h, the sample was collected and dried under vacuum at 100℃ overnight to give **2DPAV-HATN-P** (6.9 mg) as yellow solid with a 94% yield and characterized via PXRD, FT-IR and ^{13}C CP NMR spectroscopy, as shown in Supplementary Fig. 49.

Synthesis of 2DPAV-P-TPB

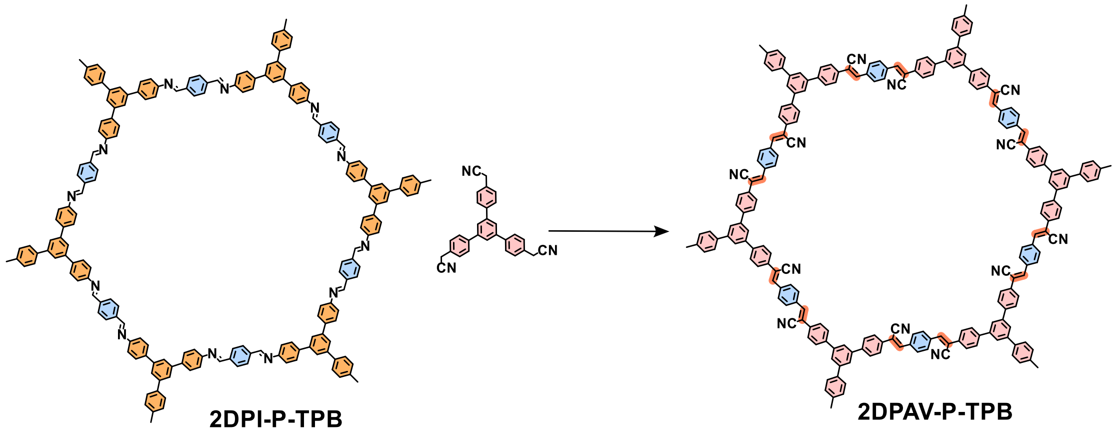

Supplementary Scheme 7. Ideal complete transformation of **2DPI-P-TPB** to **2DPAV-P-TPB** via Mannich-elimination reaction.

A glass ampoule was charged with **2DPI-P-TPB** (7 mg), 2,2'-(5'-(4-(Cyanomethyl)phenyl)-[1,1':3',1"-terphenyl]-4,4"-diyl)diacetonitrile (7 mg, 0.5 equivalent per C=N bond), Cs_2CO_3 (40 mg, 3 equivalents per C=N bond), and a mixture of DMAc/H_2O (0.7/0.3 mL). The ampoule was degassed by three freeze-pump-thaw cycles, sealed under vacuum, and heated at 120℃ for 6 days. After cooling to room temperature, the resulting powder was filtered and sequentially washed with dimethylformamide, H_2O, ethanol and acetone. After Soxhlet extraction with tetrahydrofuran for 18 h, the sample was collected and dried under vacuum at 100℃ overnight, yielding **2DPAV-P-TPB** as yellow solid with a 90% yield and and characterized via PXRD, and FT-IR spectroscopy, as shown in Supplementary Fig. 50.

Synthesis of 2DPAV-DMP-TPB

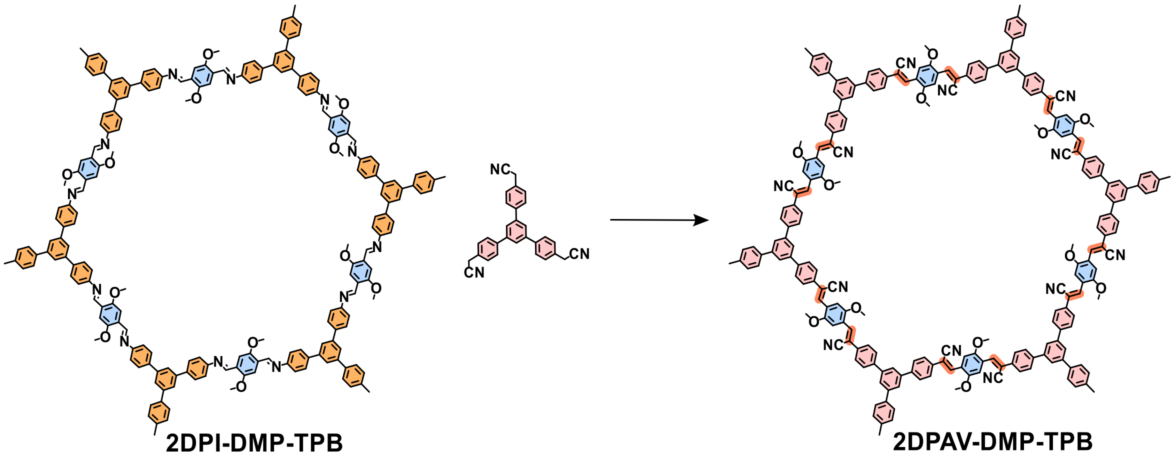

Supplementary Scheme 8. Ideal complete transformation of **2DPI-DMP-TPB** to **2DPAV-DMP-TPB** via Mannich-elimination reaction.

A glass ampoule was charged with **2DPI-DMP-TPB** (5 mg), 2,2'-(5'-(4-(cyanomethyl)phenyl)-[1,1':3',1"-terphenyl]-4,4"-diyl)diacetonitrile (10 mg, 1.2 equivalents per C=N bond), Cs_2CO_3 (50 mg, 8 equivalents per C=N bond), and a mixture of DMAc/H_2O (0.5/0.1 mL). Afterwards, the mixture was sonicated for 10 minutes at room temperature and the ampoule was degassed by three freeze-pump-thaw cycles, sealed under vacuum, and heated at 130℃ for 10-12 days. After cooling to room temperature, the resulting powder was filtered and sequentially washed with dimethylformamide, H_2O, ethanol and acetone. After Soxhlet extraction with acetone for 18 h, the sample was collected and dried under vacuum at 100℃ overnight to give **2DPAV-DMP-TPB** (2.6 mg) as a dark-orange solid with a 48% yield and characterized via PXRD, FT-IR, Raman, and ^{13}C CP NMR spectroscopy, as shown in Supplementary Figs. 51 and 52.

General synthesis of 2D PAVs using Knoevenagel 2D polycondensation

A glass ampoule containing C_3 aldehyde monomer (30 µmol), C_2 acetonitrile monomer (45 µmol), and 0.1 mL Cs_2CO_3 (0.1 M, aqueous solution) were mixed with 1.0 mL dimethylacetamide (DMAc). The ampoule was then sealed under vacuum after three freeze-pump-thaw cycles. Afterwards, the mixture was sonicated for 1~3 minutes at room temperature and heated at 120 °C for 3 days. After cooling down to room temperature, the resulting precipitated sample was filtered and sequentially washed three times with

DMF, water, and ethanol yielding amorphous **2DPAV-BTT-P**, **2DPAV-BTT-BP**, **2DPAV-BTT-BPY** and **2DPAV-BTT-BT**.

Section C. Supplementary Figures.

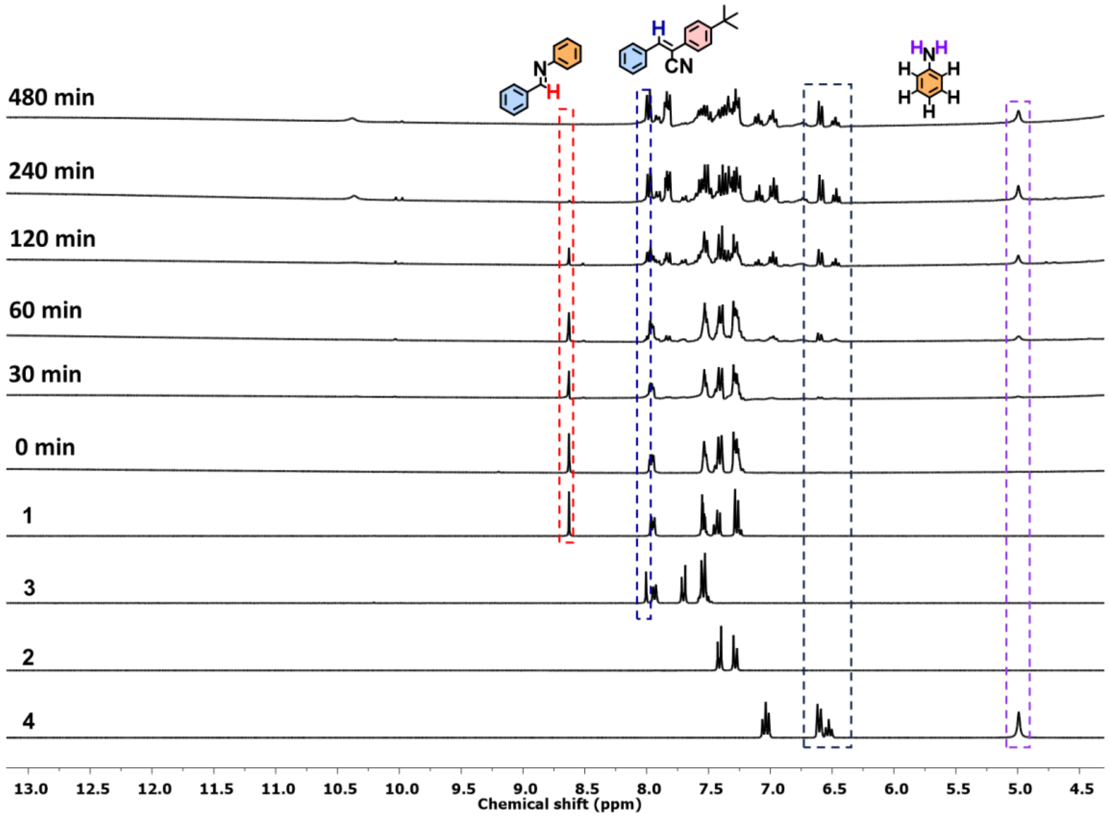

Supplementary Figure 1. *In-situ* ^{1}H NMR spectrum, monitoring of the Mannich-elimination reaction between compounds **1** and **2** in DMAc, with H_2O (0.1 mL per mL DMAc) and Cs_2CO_3 (2 equivalents per **1**) at 120 °C for 8 h in DMSO-d$_6$.

At specified intervals during the Mannich-elimination reaction in the Schlenk tube, aliquots 0.5 mL of the reaction mixture were withdrawn and mixed with 0.1 mL of DMSO-d$_6$ in an NMR tube for *in-situ* analysis, as depicted in Supplementary Figure 1.

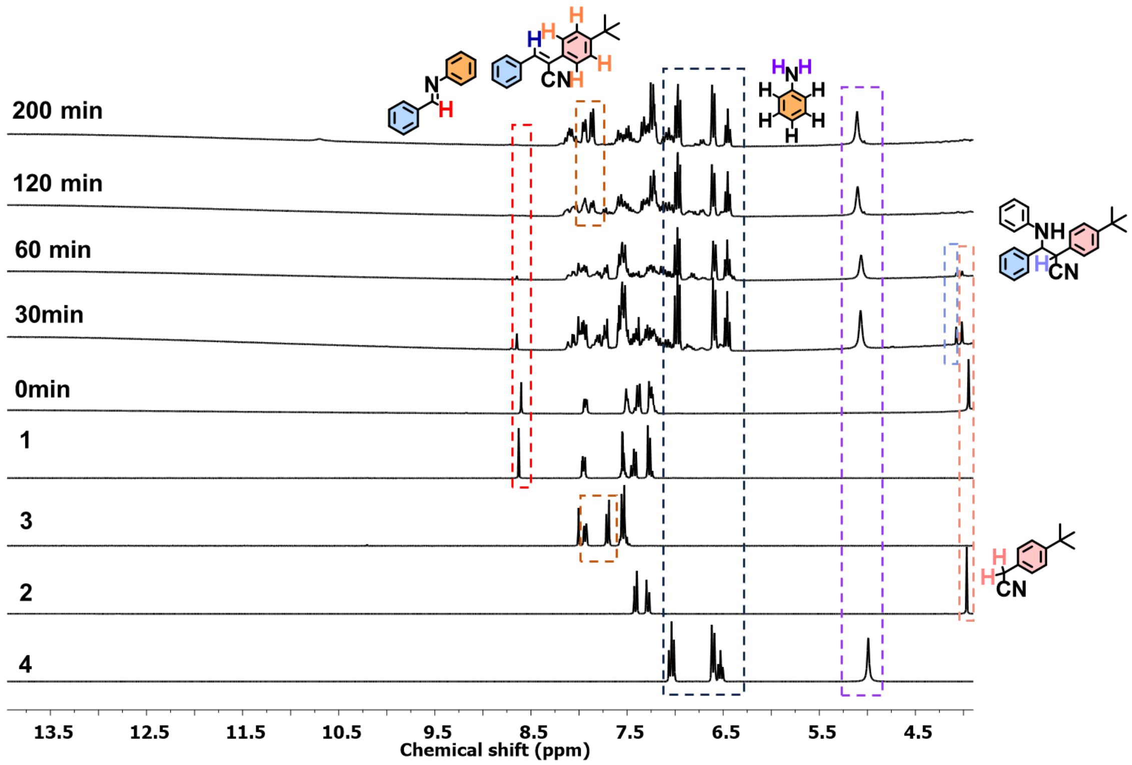

Supplementary Figure 2. *In-situ* ^{1}H NMR spectrum, monitoring of the Mannich reaction between compounds **1** and **2** in DMAc with Cs_2CO_3 (2 equivalents per **1**) at 120 °C for 4 h in DMSO-d$_6$.

At specified intervals during the Mannich-elimination reaction in the Schlenk tube, aliquots of 0.5 mL of the reaction mixture were withdrawn and mixed with 0.1 mL of DMSO-d$_6$ in an NMR tube for *in-situ* analysis, as depicted in Supplementary Figure 2.

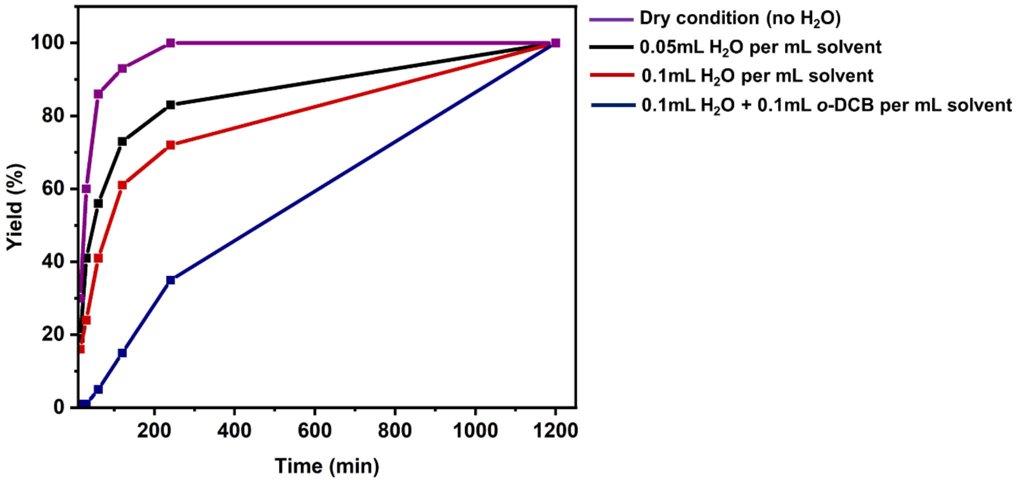

Supplementary Figure 3. Graph illustrating the *in-situ* analysis of the Mannich-elimination reaction, showing yield (%) versus reaction time (min) as a function of the water content. The purple line represents the reaction conducted using 1 mL of DMAc as the unique solvent, without the presence of water. The black line corresponds to 0.95 mL of DMAc with 0.05 mL of water. The red line indicates 0.90 mL of DMAc with 0.1 mL of water. Finally, the blue line represents 0.90 mL of DMAc with 0.05 mL of water and 0.05 mL of *o*-DCB as a non-polar solvent.

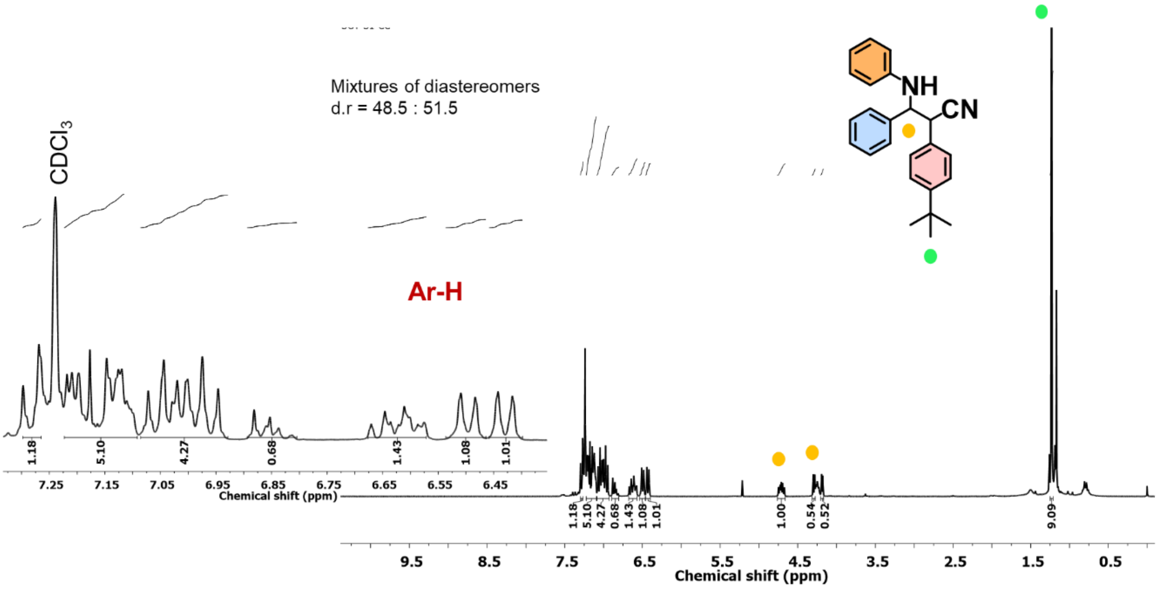

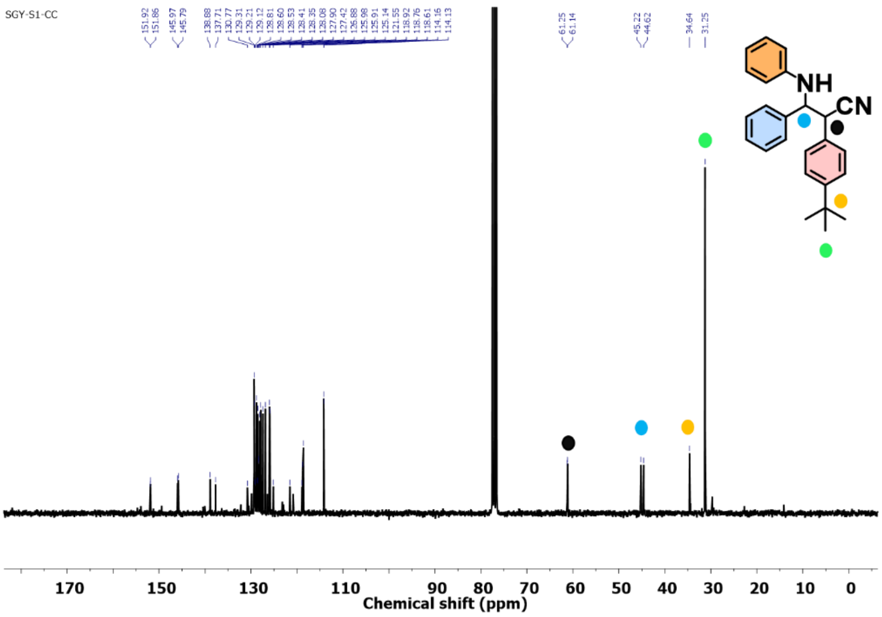

Supplementary Figure 4. ^{1}H NMR spectrum and ^{13}C NMR spectrum (300 MHz) of **5** in $CDCl_3$ solvent.

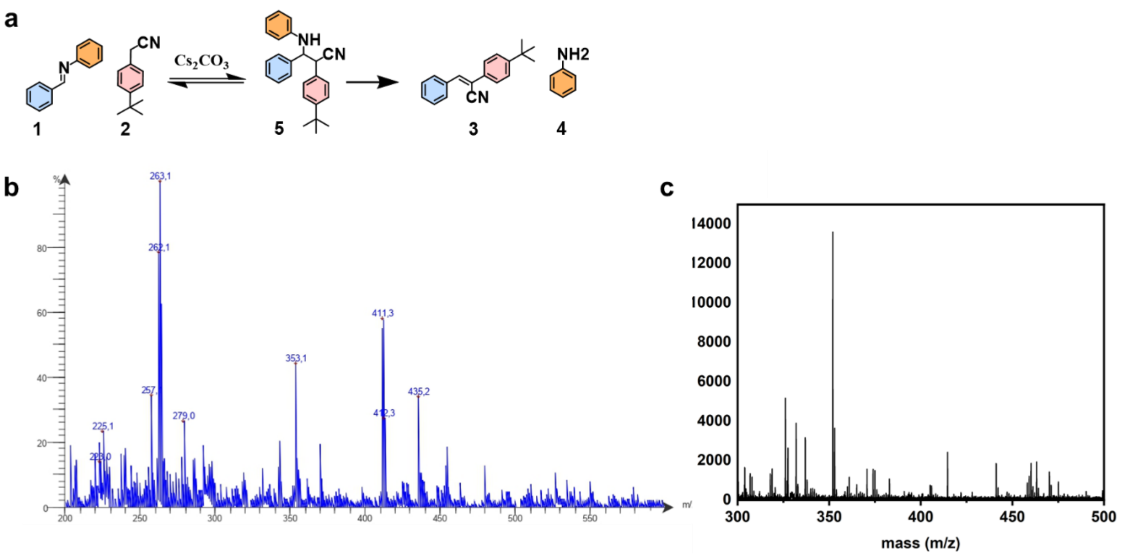

Supplementary Figure 5. a, Mannich-elimination reaction pathway. **b**, ESI spectrum showed showing the m/z values of 263.1, and 353.1, corresponding to compounds for **3** and **5** , respectively. **c**, Maldi spectrum shows the intermediate mass, further supporting the Mannich-emilination mechanism.

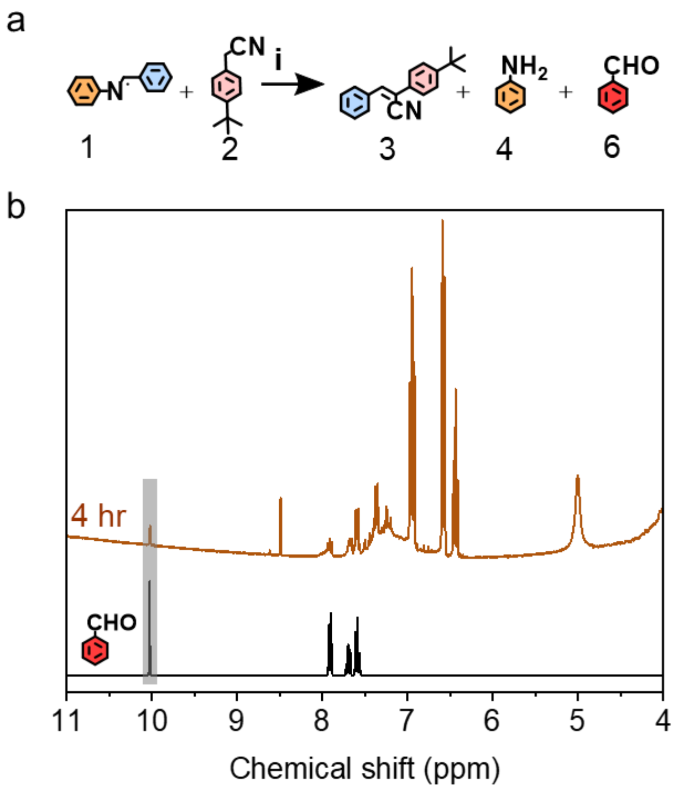

Supplementary Figure 6. a, Schematic synthesis of model compound **3** along with side reaction, hydrolysis. i: KOH, DMAc/H_2O (10/1), 120℃, 4 h. **b,** ^{1}H-NMR analysis of model reaction performed in DMAc. The spectrum of compounds **6** measured in DMSO-

d_6 are shown for comparison. The in-situ spectrum are recorded in DMAc/H_2O with trace DMSO-d_6 for calibration.

Further investigation into the KOH system revealed the presence of benzaldehyde as a side product after the reaction, suggesting that water in presence of strong inorganic base promotes competing hydrolysis pathways of imine units alongside the Mannich-elimination mechanism. This accounts for the observed yield reduction in the KOH system, while the Cs_2CO_3 system appears less sensitive to water-induced side reactions (Supplementary Fig. 1) and promotes high yield and efficiency with extended reaction time, which in turn helps towards high crystallinity of 2D PAVs.

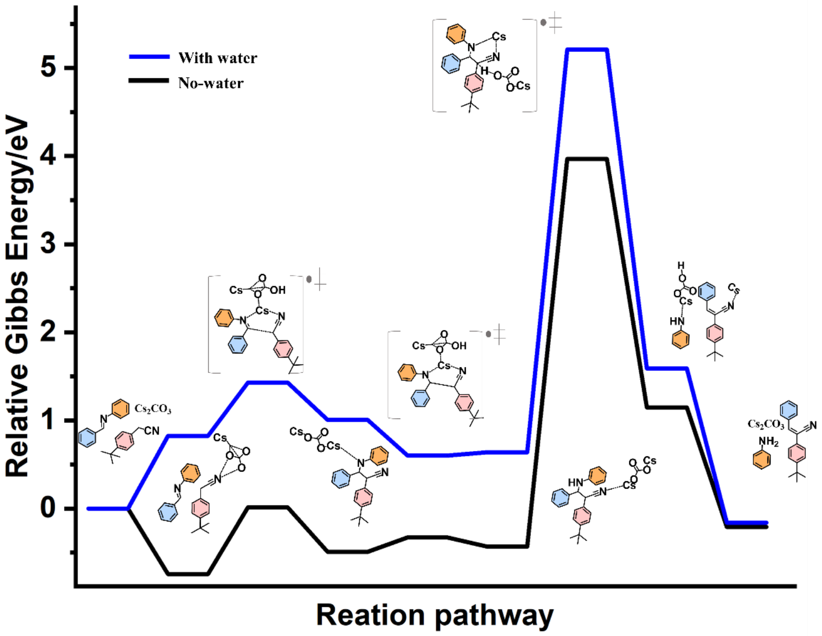

Supplementary Figure 7. Reaction mechanism and calculated Gibbs free energies for the Mannich-elimination reaction. The mechanism highlights the crucial role of water, demonstrating that in the absence of water, the intermediate and transition states exhibit lower energy levels compared to those in its presence.

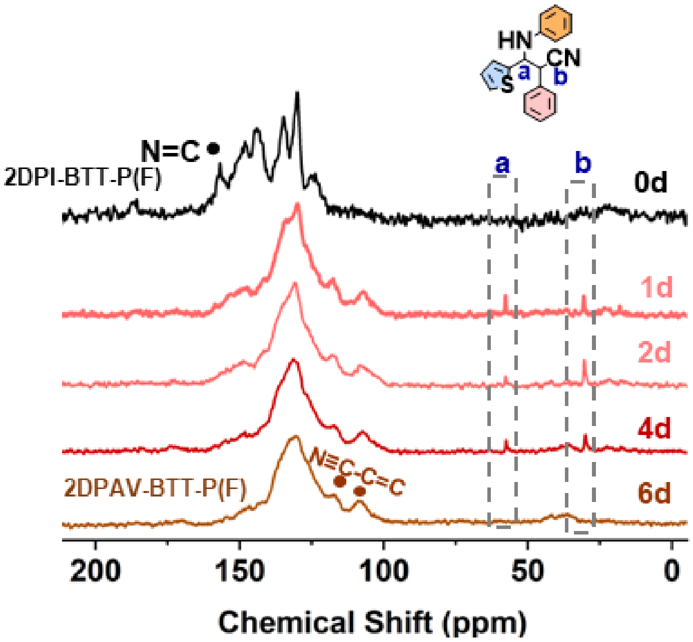

Supplementary Figure 8. Solid state ^{13}C CP NMR spectrum tracking the transformation of **2DPI-BTT-P(F)** to **2DPAV-BTT-P(F)** over 6 days, highlighting the intermediate evolution and offering detailed insights into the reaction pathway.

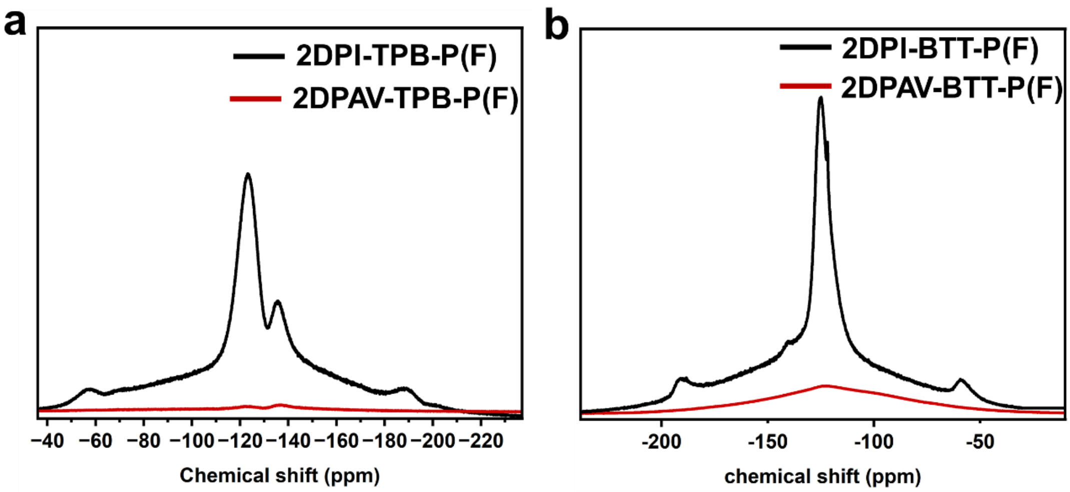

Supplementary Figure 9. a, ^{19}F-solid state NMR spectrum of the pristine imine-linked **2DPI-TPB-P(F)** (black line) and fully transformed **2DPAV-TPB-P(F)** (red line). **b**, ^{19}F-solid state NMR spectrum of the pristine imine-linked **2DPI-BTT-P(F)** (black line) and fully transformed **2DPAV-BTT-P(F)** (red line).

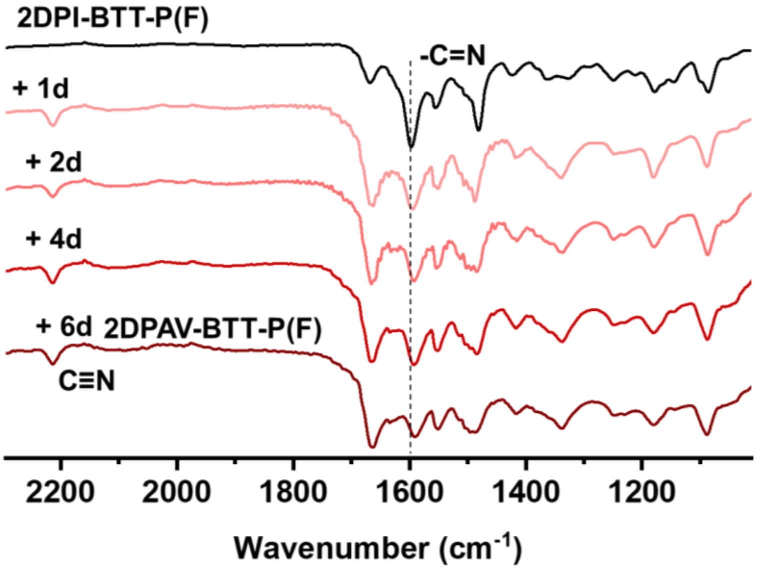

Supplementary Figure 10. FT-IR spectrum monitoring the transformation from **2DPI-BTT-P(F)** to **2DPAV-BTT-P(F)** over 6 days, highlighting the evolution of the characteristic -C≡N and -C=C vibrations.

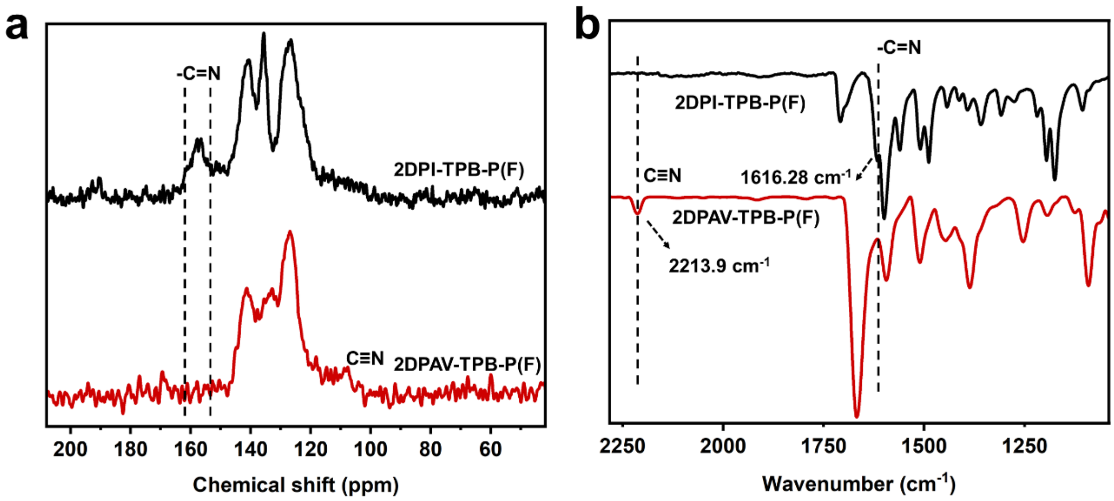

Supplementary Figure 11. a, solid state ^{13}C CP NMR spectrum of pristine imine-linked **2DPI-TPB-P(F)** (black line) and fully transformed **2DPAV-TPB-P(F)** (red line). **b**, FT-IR spectrum of pristine imine-linked **2DPI-TPB-P(F)** (black line) and fully transformed **2DPAV-TPB-P(F)** (red line).

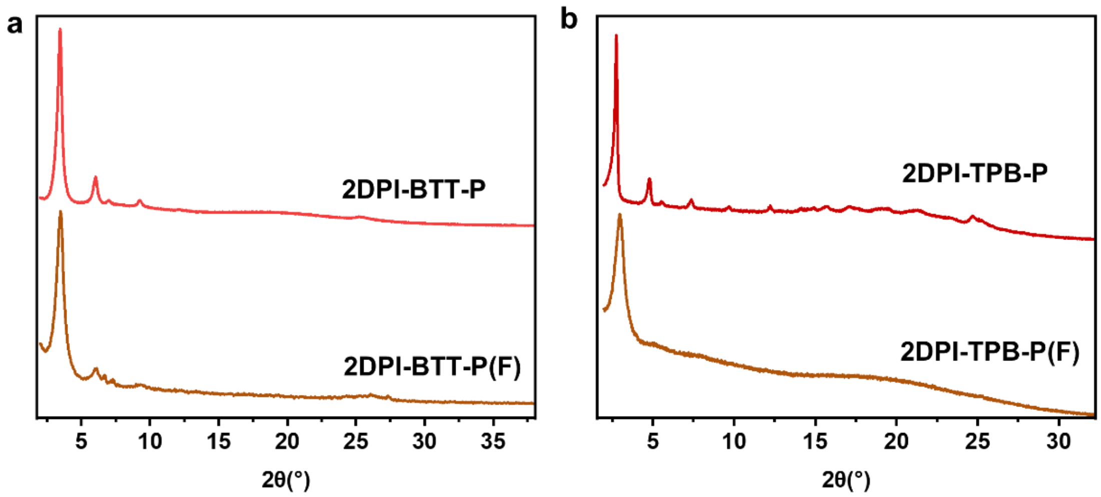

Supplementary Figure 12. Comparison of the PXRD patterns of **a,** fluorine-labelled **2DPI-BTT-P** (red line) and **2DPI-BTT-P(F)** (orange line), and **b,** fluorine-labelled **2DPI-TPB-P** (red line) and **2DPI-TPB-P(F)** (orange line).

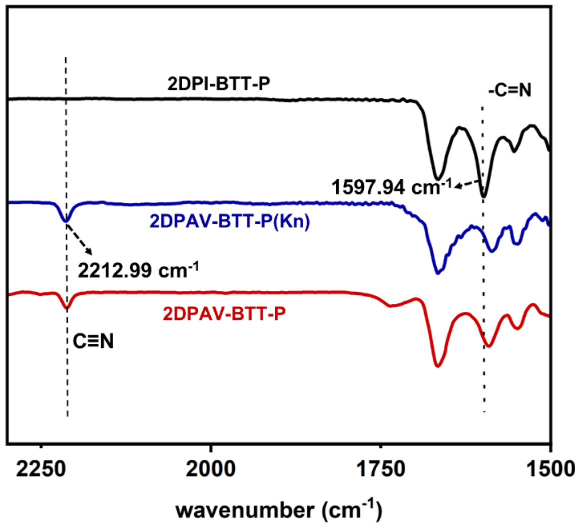

Supplementary Figure 13. FT-IR spectrum of **2DPI-BTT-P** (black line), **2DPAV-BTT-P** (red line), and amorphous **2DPAV-BTT-P(Kn)** (blue line) synthesized via Knoevenagel condensation reaction between **BTTC** and **PDAN**.

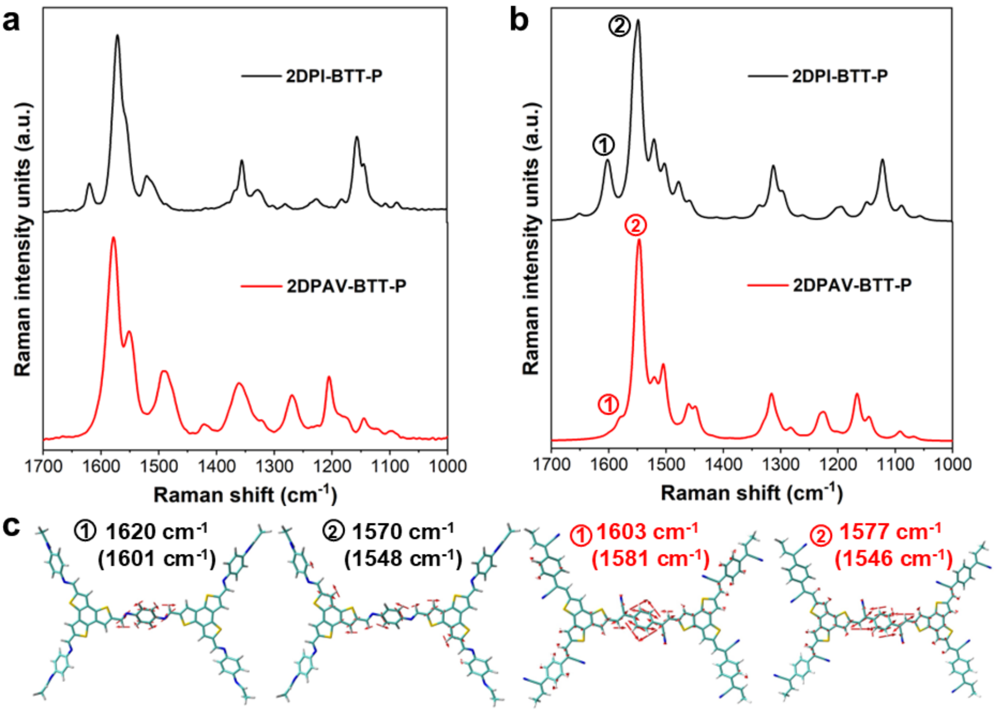

Supplementary Figure 14. a, Experimental and **b,** DFT-simulated (**b**) Raman spectrum of **2DPI-BTT-P** (black line) and **2DPAV-BTT-P** (red line), respectively. **c**, Vibrational eigenvectors associated with the most outstanding Raman features of **2DPI-BTT-P** (black numbers) and **2DPAV-BTT-P** (red numbers). The experimental and theoretical (in parentheses) wavenumbers are also shown.

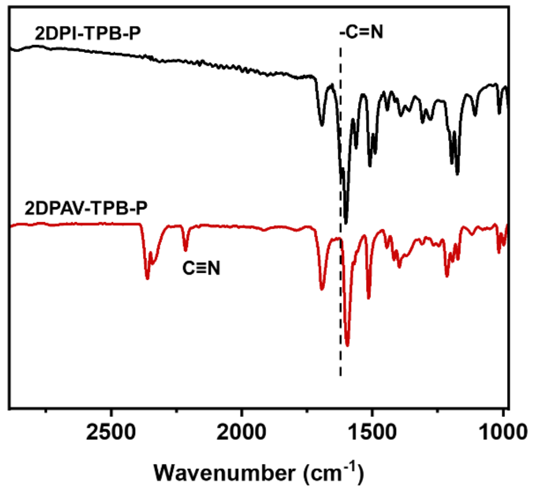

Supplementary Figure 15. FT-IR spectrum of **2DPI-TPB-P** (black line) and **2DPAV-TPB-P** (red line).

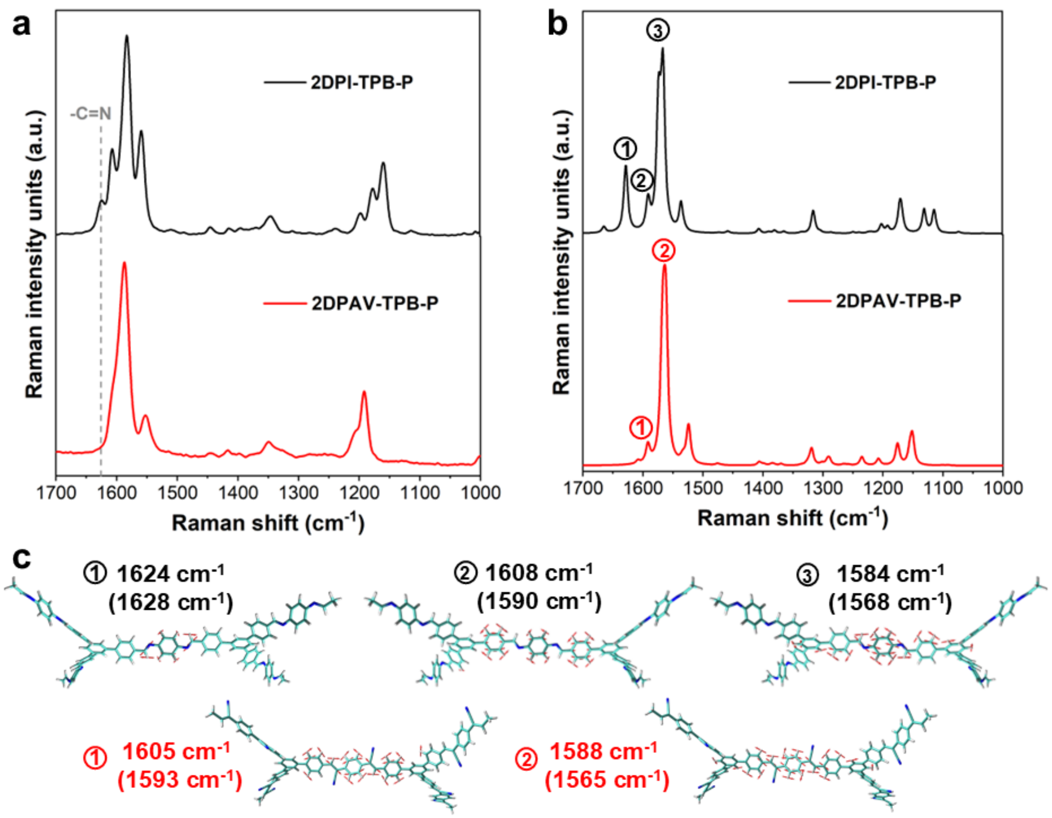

Supplementary Figure 16. a, Experimental and **b,** DFT-simulatedRaman spectrum of of **2DPI-TPB-P** (black line) and **2DPAV- TPB-P** (red line). **c**, Vibrational eigenvectors associated with the most outstanding Raman features of **2DPI-TPB-P** (black numbers) and **2DPAV- TPB-P** (red numbers). The experimental and theoretical (in parentheses) wavenumbers are also shown.

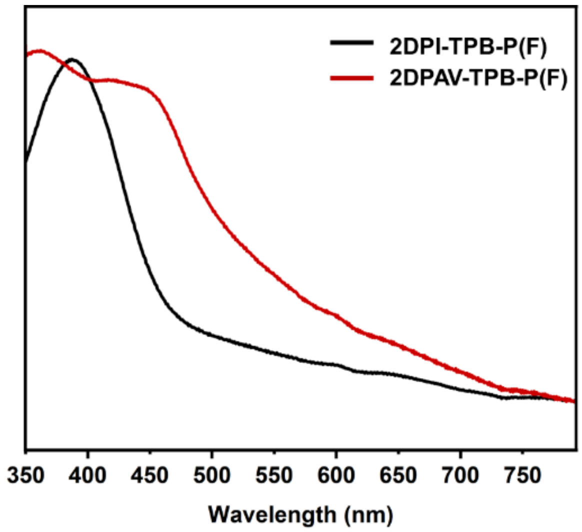

Supplementary Figure 17. UV-Visible spectrum of the pristine imine-linked **2DPI-TPB-P(F)** (black line) and fully transformed **2DPAV-TPB-P(F)** (red line), evidencing a significant redshift in the absorption spectrum of **2DPAV-TPB-P(F)**.

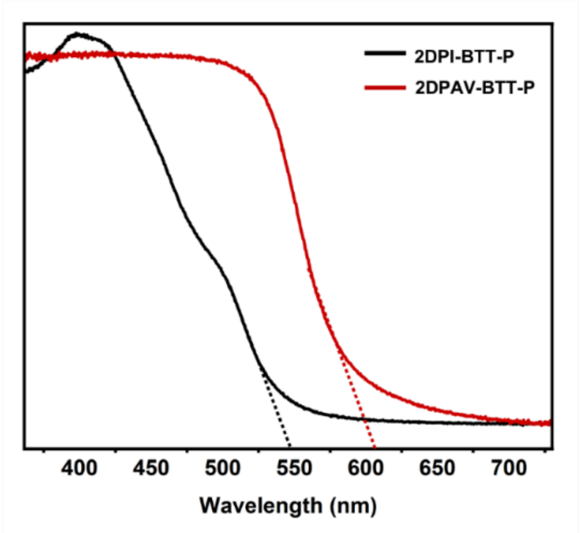

Supplementary Figure 18. UV-visible absorption spectrum of **2DPI-BTT-P** (black line) and fully transformed **2DPAV-BTT-P** (red line), showing a remarkable redshift for the absorption spectrum of **2DPAV-BTT-P**.

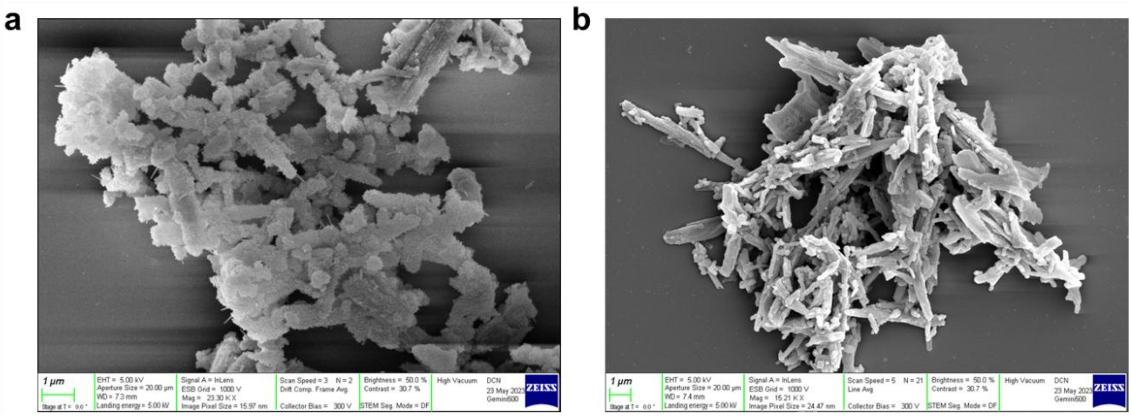

Supplementary Figure 19. SEM images of **a**, **2DPI-BTT-P** and **b, 2DPAV-BTT-P.**

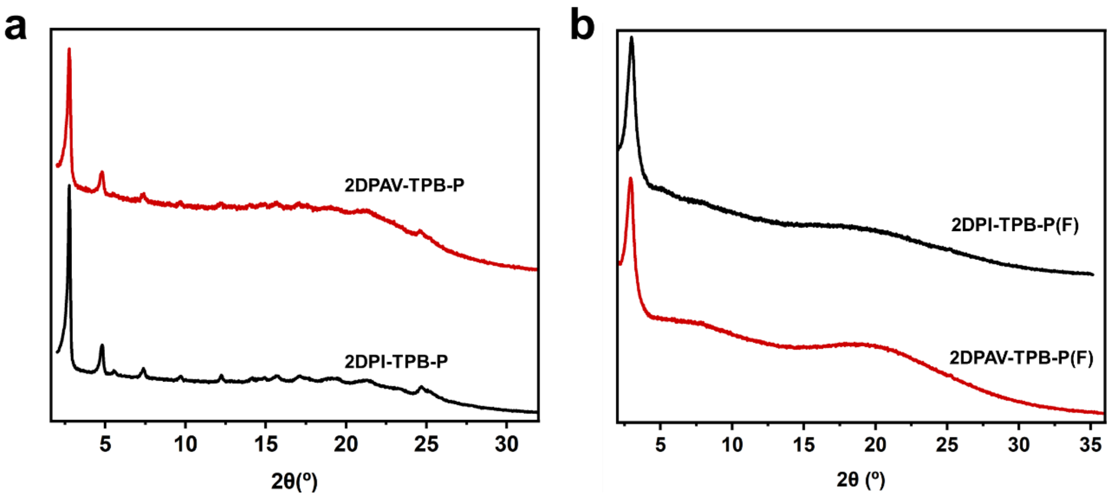

Supplementary Figure 20. a, PXRD patterns of **2DPI-TPB-P** (black line) and **2DPAV-TPB-P** (red line). **b**, PXRD patterns of **2DPI-TPB-P(F)** (black line) and **2DPAV- TPB-P(F)** (red line).

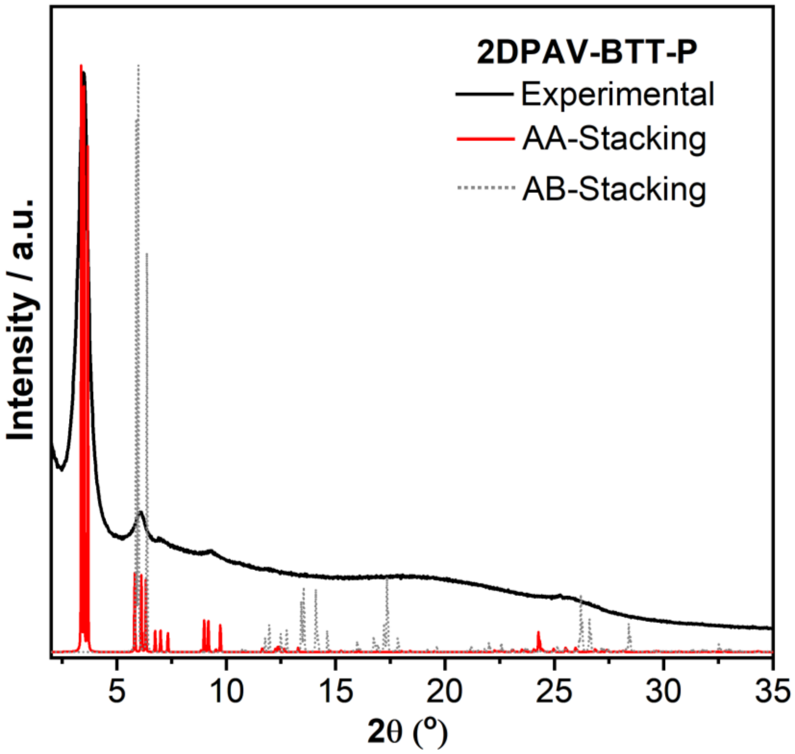

Supplementary Figure 21. Comparison of experimental diffraction data with the simulated patterns for periodic models of **2DPAV-BTT-P**, considering both AA- and AB-stacking configurations.

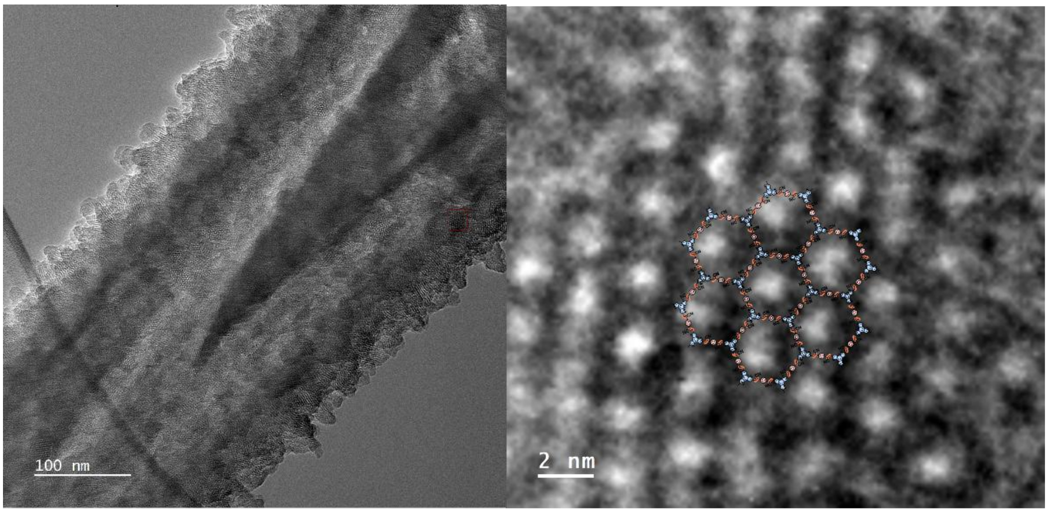

Supplementary Figure 22. TEM images of **2DPAV-BTT-P**, displaying a uniformly distributed hexagonal lattice with a distance of 2.44 nm.

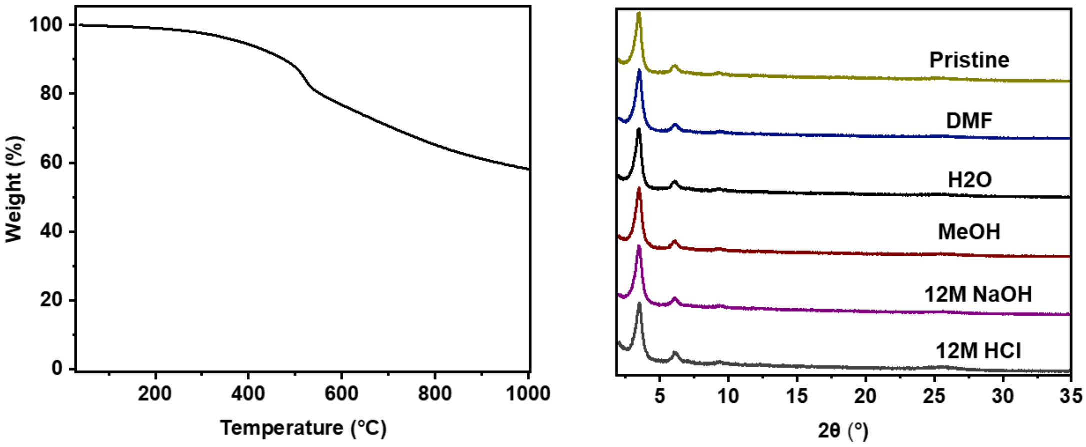

Supplementary Figure 23. | TGA for **2DPAV-BTT-P** to assess its thermal stability, along with X-ray diffraction (XRD) data under various conditions over a period of one week.

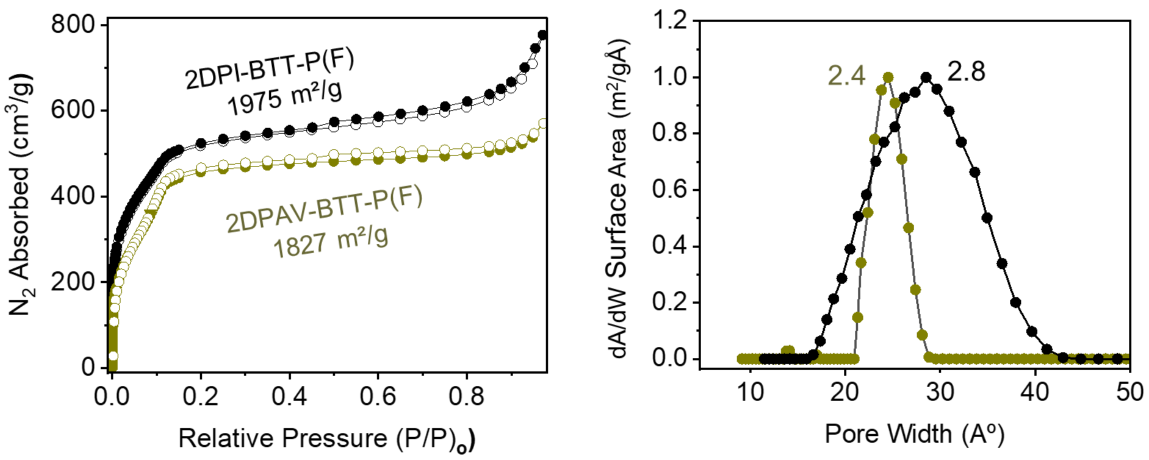

Supplementary Figure 24. N_2 Physisorption isotherms, and pore size distributions for **2DPI-BTT-P(F)** and **2DPAV-BTT-P(F).**

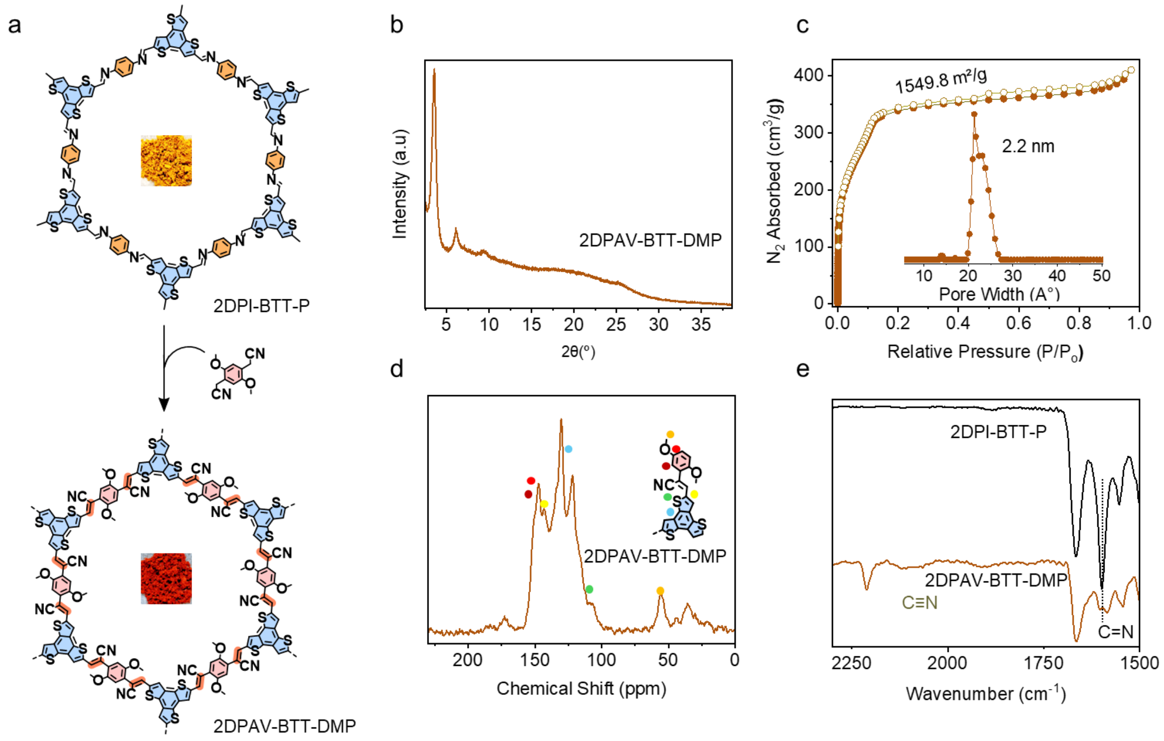

Supplementary Figure 25. a, Schematic synthesis of **2DPAB-BTT-DMP** using Mannich-elimination **b,c,d,** PXRD pattern, N_2 physisorption isotherms, and pore size distributions and ^{13}C CP NMR, respectively, for **2DPAB-BTT-DMP. e,** IR spectrum for **2DPI-BTT-P** and **2DPAV-BTT-DMP**.

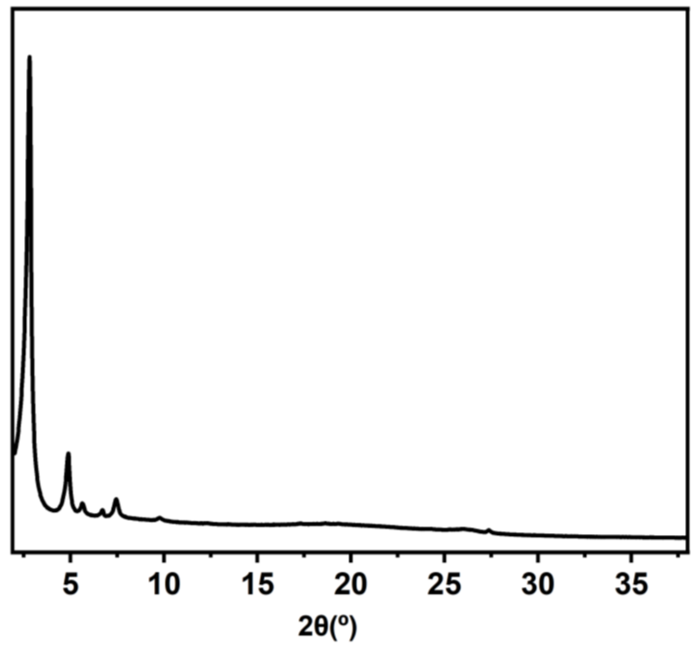

Supplementary Figure 26. PXRD pattern of **2DPI-BTT-BP.**

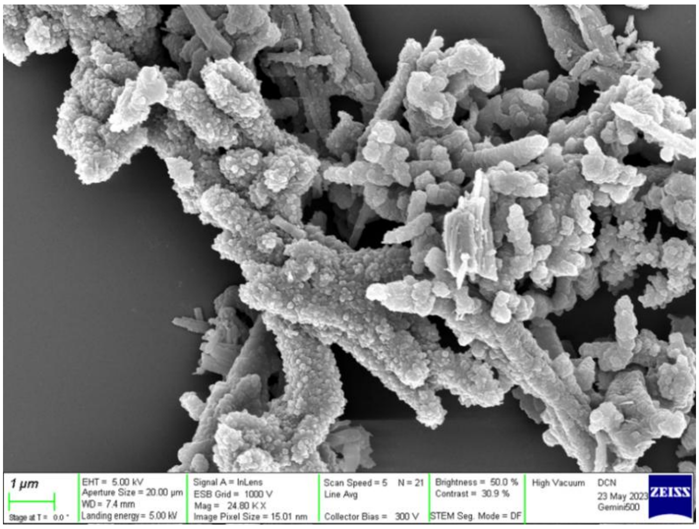

Supplementary Figure 27. SEM image of **2DPI-BTT-BP.**

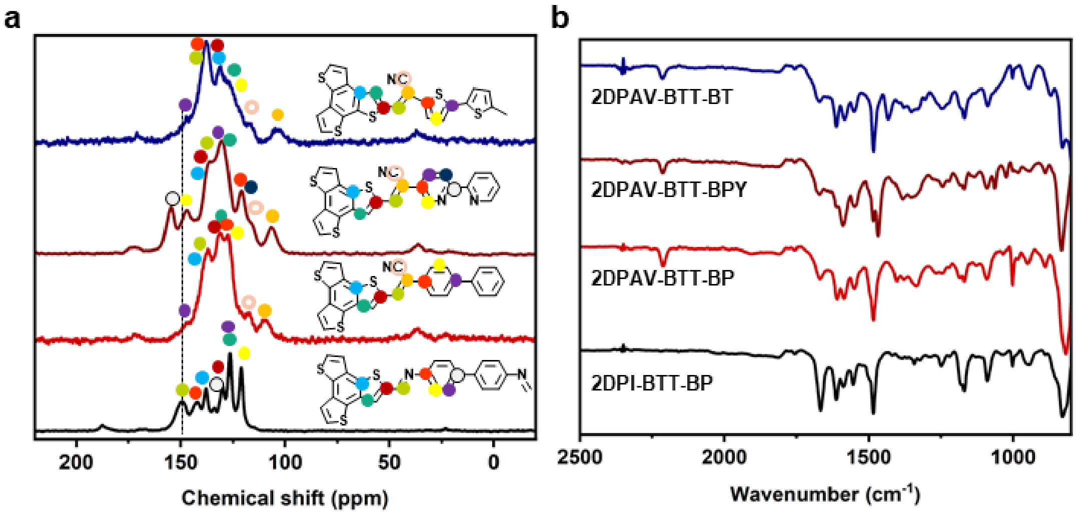

Supplementary Figure 28. a,b, Solid state ^{13}C CP NMR and FT-IR spectrum of **2DPI-BTT-BP** (black line) and its fully transformed 2DPAVs products **2DPAV-BTT-BP** (red line), **2DPAV-BTT-BPY** (dark red line), and **2DPAV-BTT-BT** (blue line).

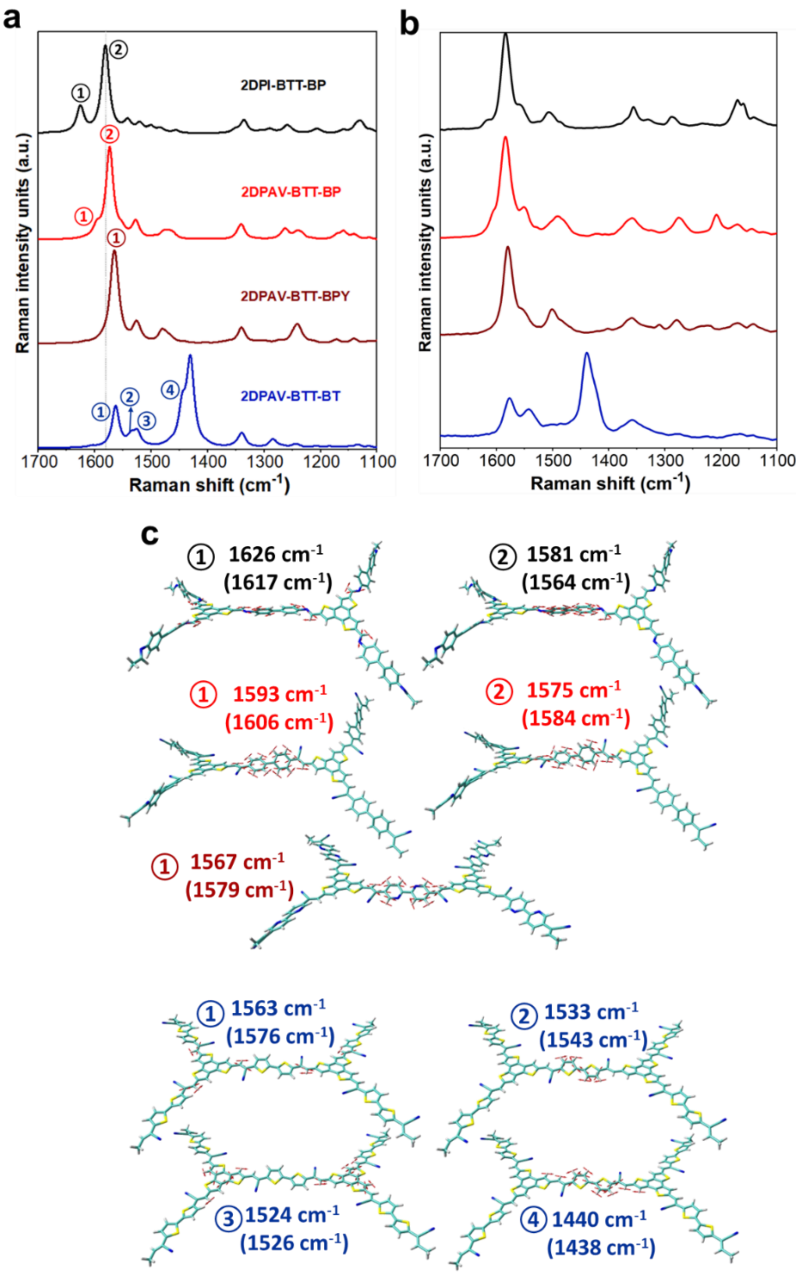

Supplementary Figure 29. a, DFT-simulated and **b,** Experimental Raman spectrum of **2DPI-BTT-BP** (black line), **2DPAV-BTT-BP** (red line), **2DPAV-BTT-BPY** (dark red line) and **2DPAV-BTT-BT** (blue line). **c**, Vibrational eigenvectors associated with the most outstanding Raman features of **2DPI-BTT-BP** (black numbers), **2DPAV-BTT-BP** (red numbers), **2DPAV-BTT-BPY** (dark red number) and **2DPAV-BTT-BT** (blue numbers). The theoretical and experimental (in parentheses) wavenumbers are also shown.

Raman spectrum indicate the disappearance of the C=N stretching vibration (localized at 1616 cm^{1} in **2DPI-BTT-BP**) in the 2D PAVs. Interstingly, the C=C/C-C stretching

vibration associated with the conjugated bridge shifts from 1585 cm^{-1} in **2DPI-BTT-BP** to 1583 cm^{-1} (**2DPAV-BTT-BP**), 1579 cm^{-1} (**2DPAV-BTT-BPY**), and 1438 cm^{-1} (**2DPAV-BTT-BT**); this reflects effective modulation of π-conjugation and electronic communication between BTT cores as a function of the different linkers. These experimental findings are nicely reproduced by theoretical modeling. The slight Raman shift from **2DPI-BTT-BP** to **2DPAV-BTT-BP** reveals the crucial role played by the biphenyl rings that remain highly distorted in both polymers, constraining the p-electron delocalization despite the planarization occurring when connecting the linkers to vinylene groups in the 2D PAVs polymer (Supplementary Figs. 26 and 27). However, the introduction of bipyridine units in **2DPAV-BTT-BPY** promotes effective N-H intramolecular non-covalent interactions leading to a further structural planarization between the pyridine units, enhancing π-conjugation efficiency as demonstrated by the notable Raman shift. Notably, the incorporation of thiophene rings instead of phenyl units results in a more polarizable p-electron cloud along with a marked enhancement of molecular planarity (Supplementary Fig. 27), resulting in a softening of the stretching CC bonds and thus a pronounced Raman shift.

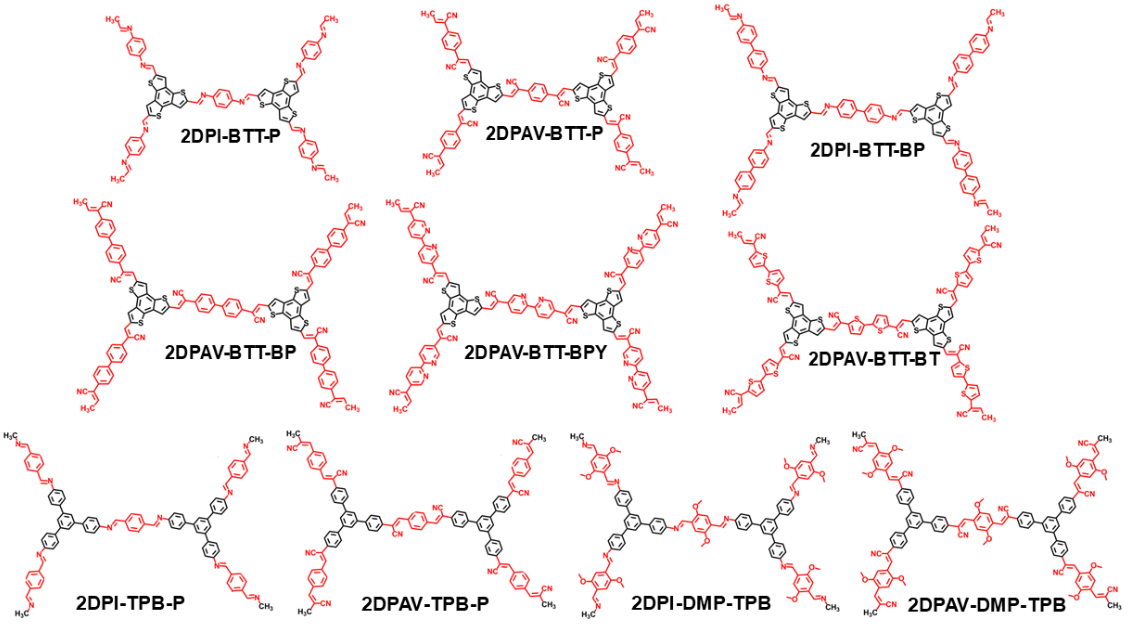

Supplementary Figure 30. Chemical structures for the dimeric models of the 2D polymers studied theoretically in this work.

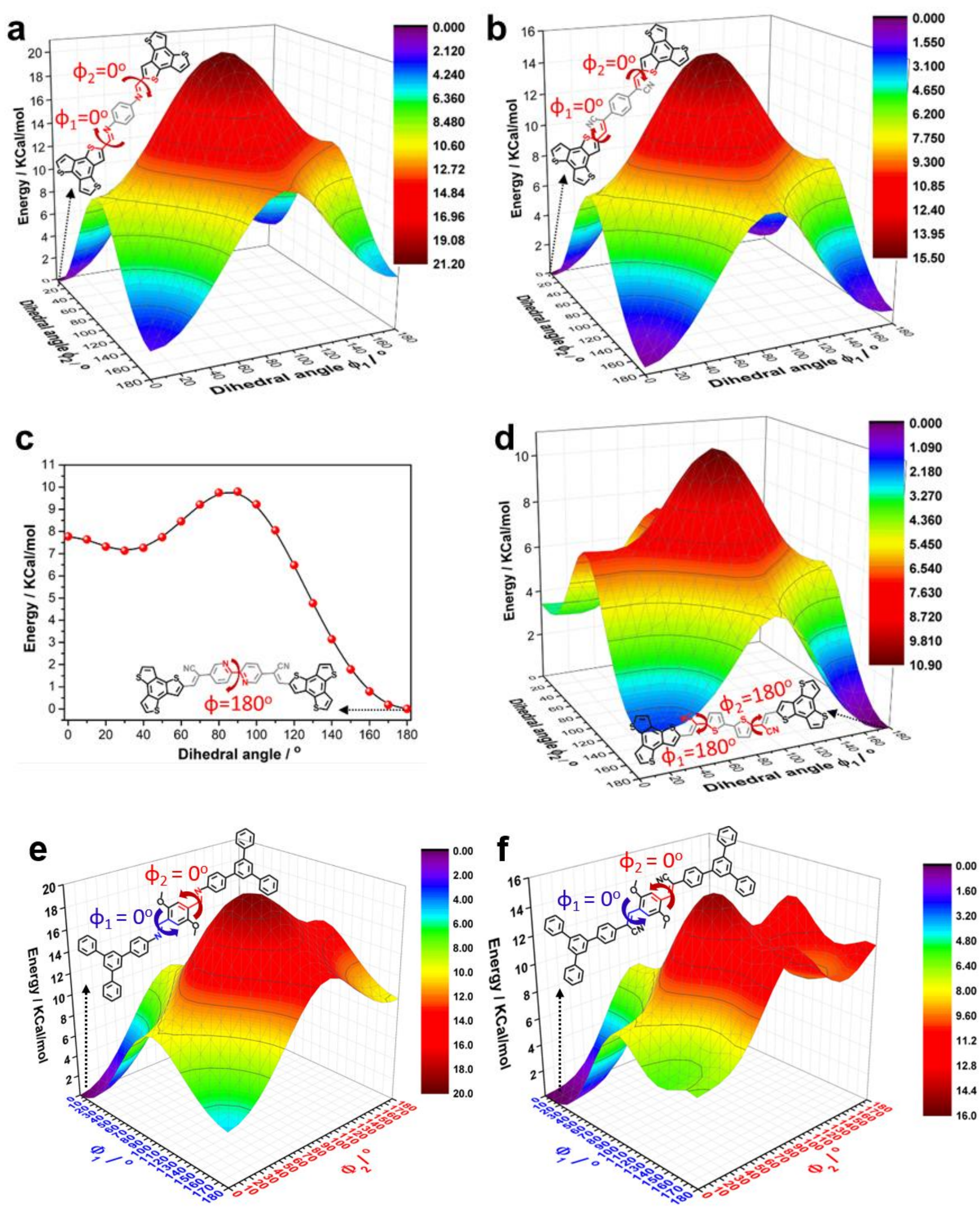

Supplementary Figure 31. Dihedral potential energy hypersurfaces calculated at the PBE0/6-31G** level of theory. The dihedral potential energy hypersurfaces are relative to the rotations of: **a**, the imine groups of **2DPI-BTT-P** and **2DPI-BTT-BP**, and **b**, the nitrile groups of **2DPAV-BTT-P** and **2DPAV-BTT-BP** with respect the adjacent benzo[1,2-b:3,4-b':5,6-b'']trithiophene platforms, **c**, the 2,2´-bipyridine unit of **2DPAV-BTT-BPY**, **d**, the nitrile groups with respect the adjacent thiophene rings of **2DPAV-BTT-BT** and **e**, **f**, the imine and nitrile groups of **2DPI-DMP-TPB** and **2DPAV-DMP-TPB**, respectively, with respect the central dimethoxybenzene moiety. Dihedral angle values of the most stable conformers are also shown.

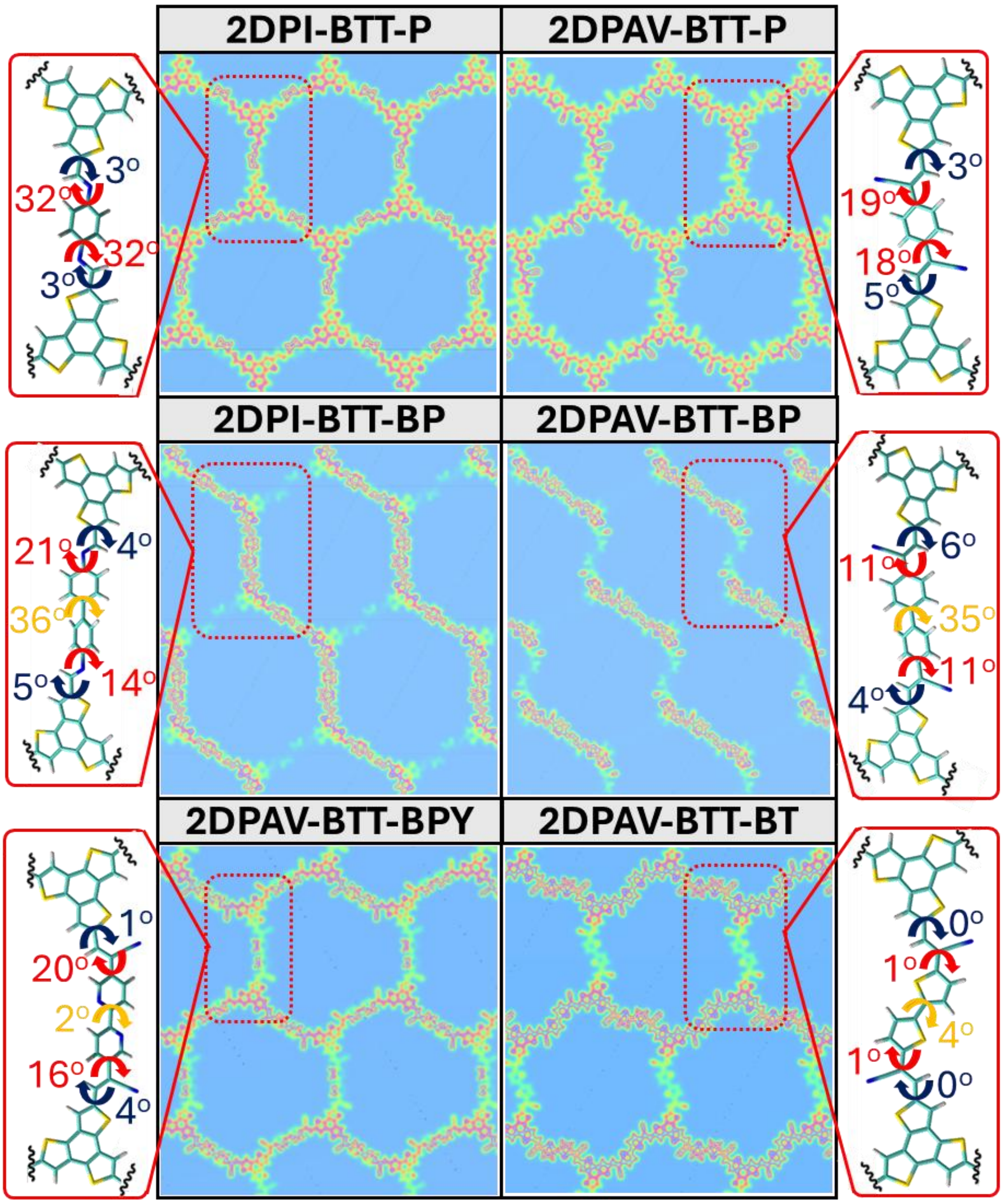

Supplementary Figure 32. DFT-PBE-calculated surface charge density color maps in the xy-plane (at the z-position) for all the BTT based 2D polymers under study. The dihedral angles between the conjugated cores and the π-bridges are also shown.

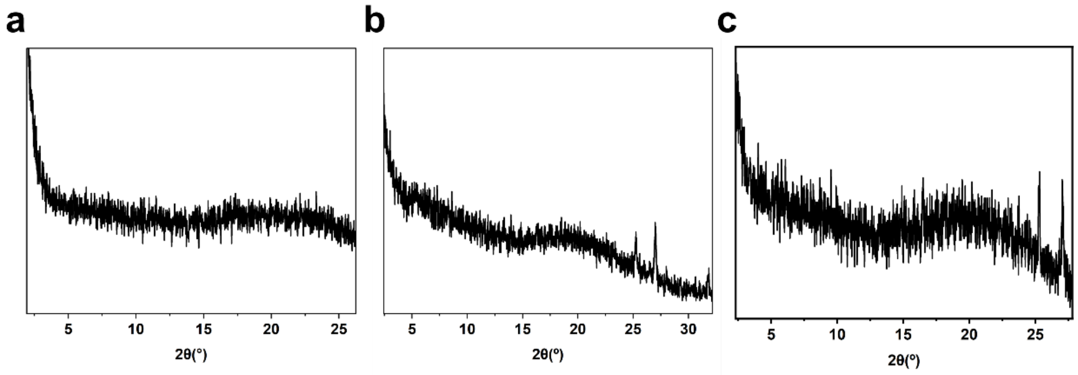

Supplementary Figure 33. a-c, PXRD patterns of directly synthesized **2DPAV-BTT-BP(Kn)**, **2DPAV-BTT-BPY(Kn)** and **2DPAV-BTT-BT(Kn)**, respectively. The synthetic procedure discussed in the section Supplementary Material and Synthetic Procedures.

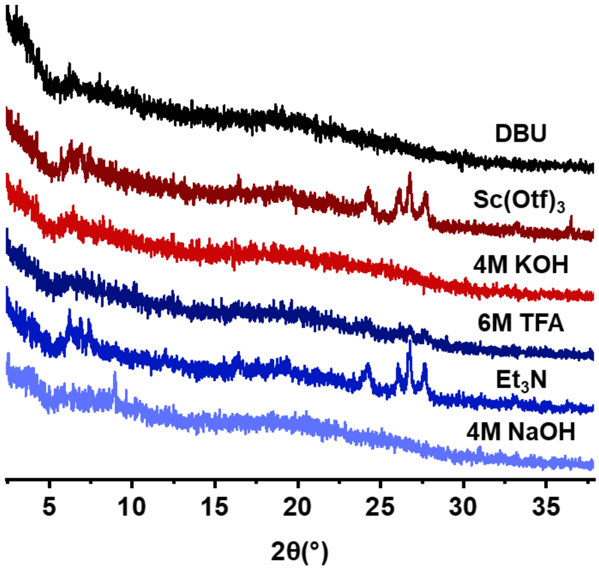

Supplementary Figure 34. PXRD pattern our various **2DPAV-BTT-P(Kn)** samples from various catalysts.

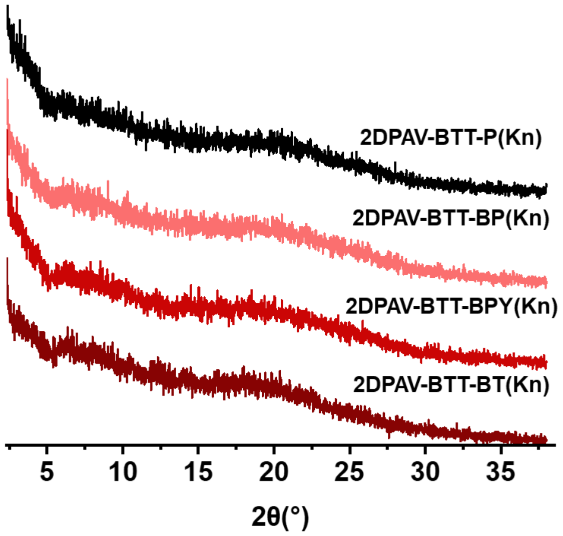

Supplementary Figure 35. PXRD pattern our various **2DPAV-BTT-P(Kn), 2DPAV-BTT-BP(Kn), 2DPAV-BTT-BPY(Kn),** and **2DPAV-BTT-BT(Kn).**

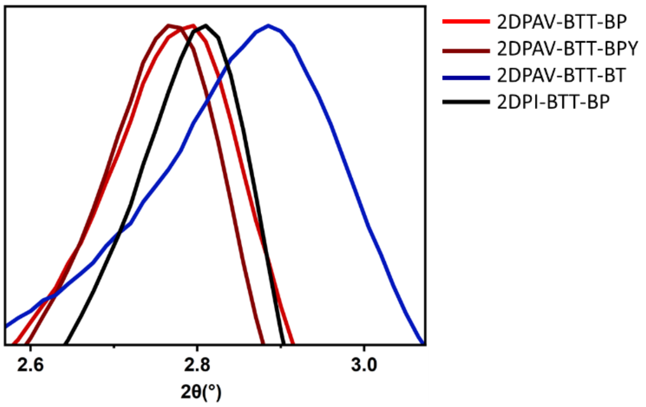

Supplementary Figure 36. PXRD patterns highlighting the (100) peak for **2DPI-BTT-BP** (black line), **2DPAV-BTT-BP** (red line), **2DPAV-BTT-BPY** (dark red line) and **2DPAV-BTT-BT** (blue line).

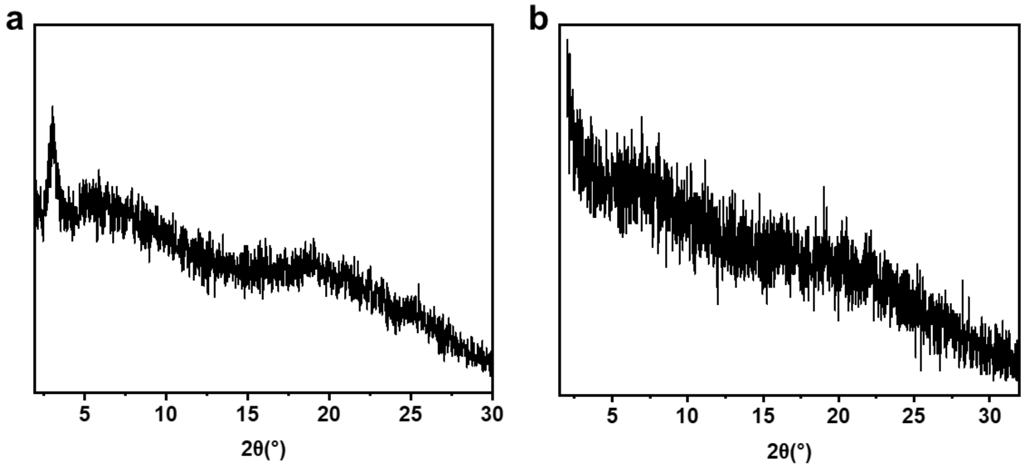

Supplementary Figure 37. a, PXRD pattern of **2DPAV-BTT-P(LM)** synthesized from pristine **2DPI-BTT-BP**. **b**, PXRD pattern of **2DPAV-BTT-BP(LM)** synthesized from pristine **2DPI-BTT-P**.

2DPAV-BTT-BP(LM) synthesized from **2DPI-BTT-P** is completely amorphous, while **2DPAV-BTT-P(LM)** synthesized from **2DPI-BTT-BP** exhibits partial crystallinity. This discrepancy can be attributed to the influence of pore size and lattice compatibility on the reaction dynamics. For **2DPAV-BTT-BP(LM)**, the precursor **2DPI-BTT-P** possesses a smaller pore size compared to the target structure, making it challenging for the long biphenyl monomer to effectively penetrate. This likely results in a vertical, random reaction within the pores, leading to an amorphous polymer due to the lack of ordered alignment.

In contrast, despite lattice mismatch between the precursor and the product, the large pore size of **2DPI-BTT-BP** allows for easier access of the phenyl monomer to the reaction site, thus resulting in moderate crystallinity for **2DPAV-BTT-P(LM)**. However, the significant dissimilarity in lattice sizes between **2DPI-BTT-BP** and **2DPAV-BTT-P(LM)** prevents the formation of a highly crystalline structure. These insights highlight the critical role of pore size and structural compatibility

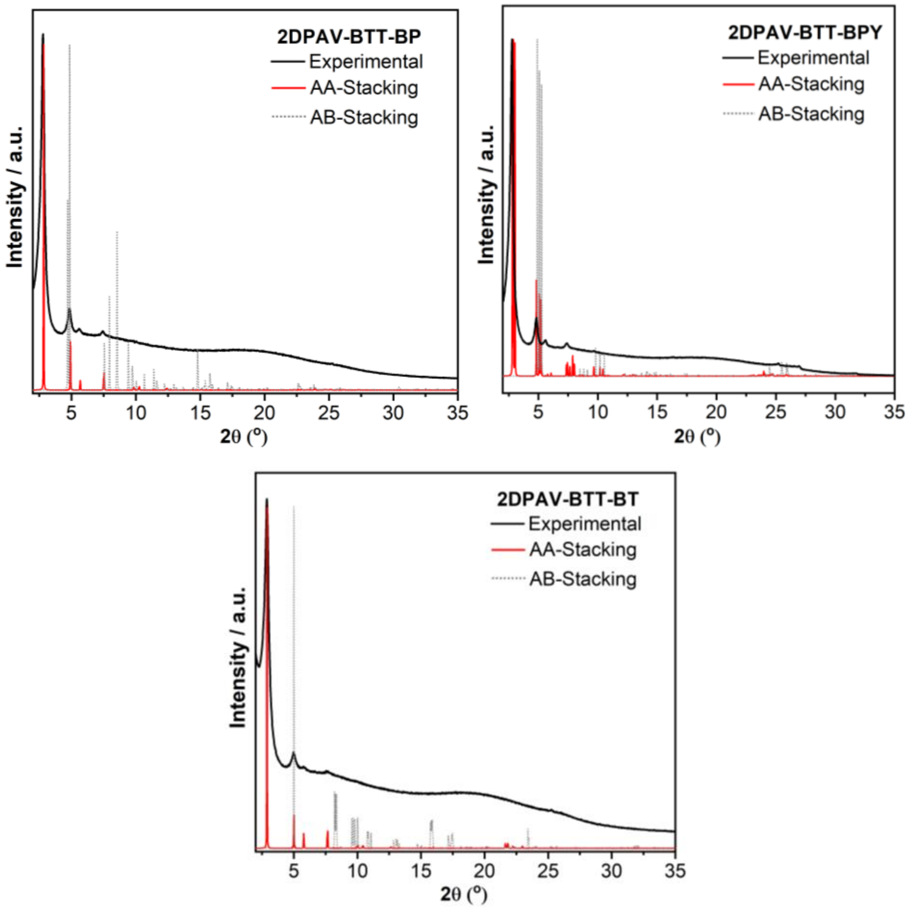

Supplementary Figure 38. Comparison of experimental diffraction data with the simulated patterns for periodic models of **2DPAV-BTT-BP**, **2DPAV-BTT-BPY**, and **2DPAV-BTT-BT**, considering both AB- and AA-stacking configurations.

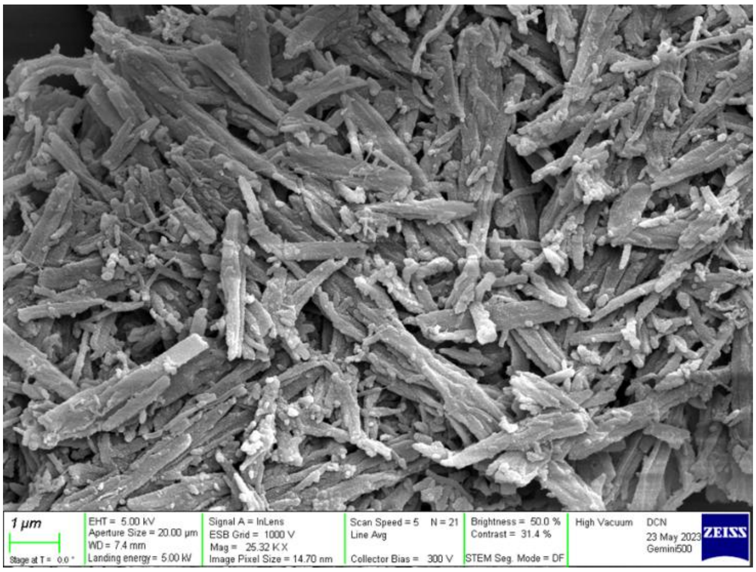

Supplementary Figure 39. SEM image of **2DPAV-BTT-BP.**

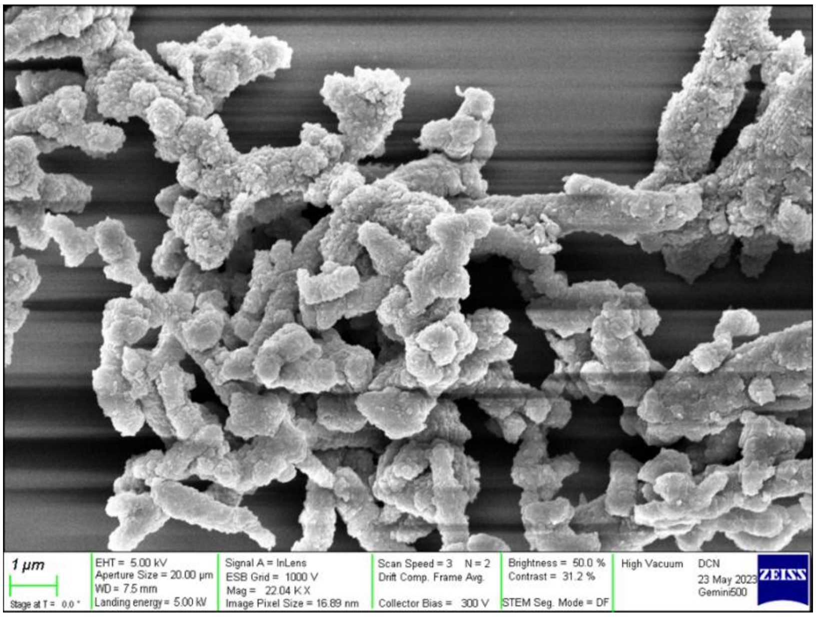

Supplementary Figure 40. SEM image of **2DPAV-BTT-BPY.**

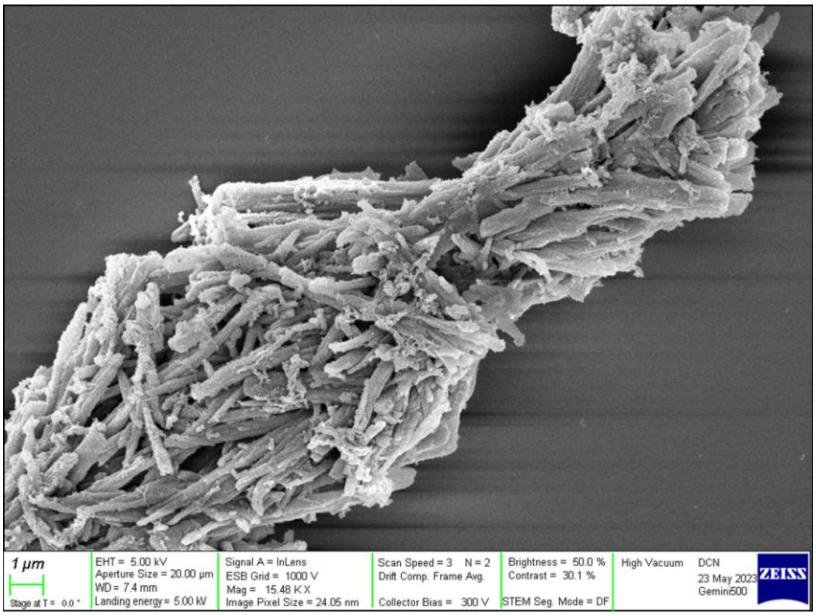

Supplementary Figure 41. SEM image of **2DPAV-BTT-BT**.

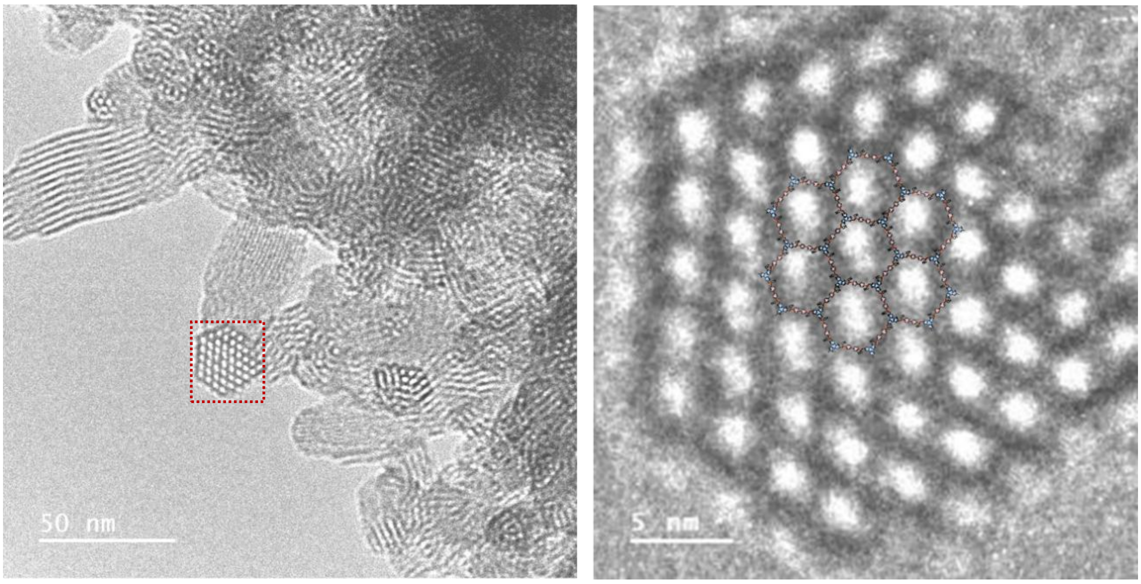

Supplementary Figure 42. TEM image of **2DPAV-BTT-BP**, displaying a uniformly distributed hexagonal lattice with a distance of 3.1 nm.

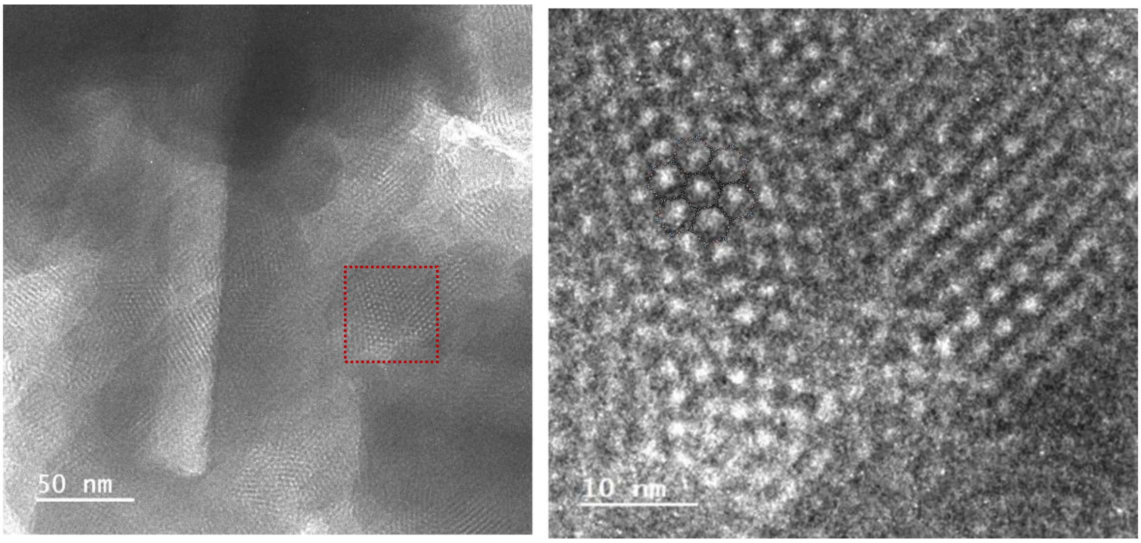

Supplementary Figure 43. TEM image of **2DPAV-BTT-BPY**, showing a uniformly distributed hexagonal lattice a distance of 3.1nm.

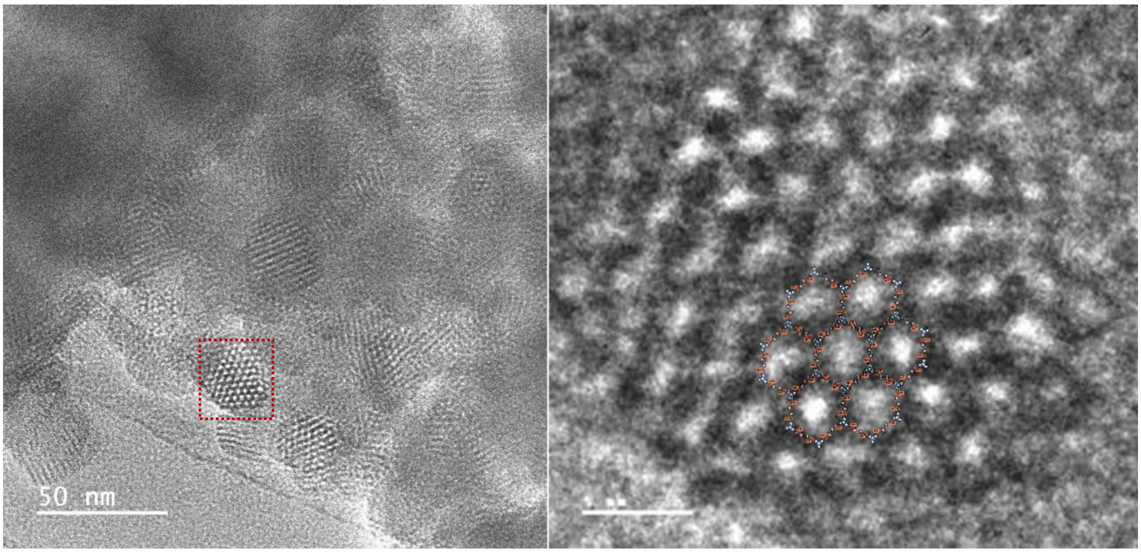

Supplementary Figure 44. TEM image of **2DPAV-BTT-BT**, displaying a uniformly distributed hexagonal lattice with a distance of 2.9 nm.

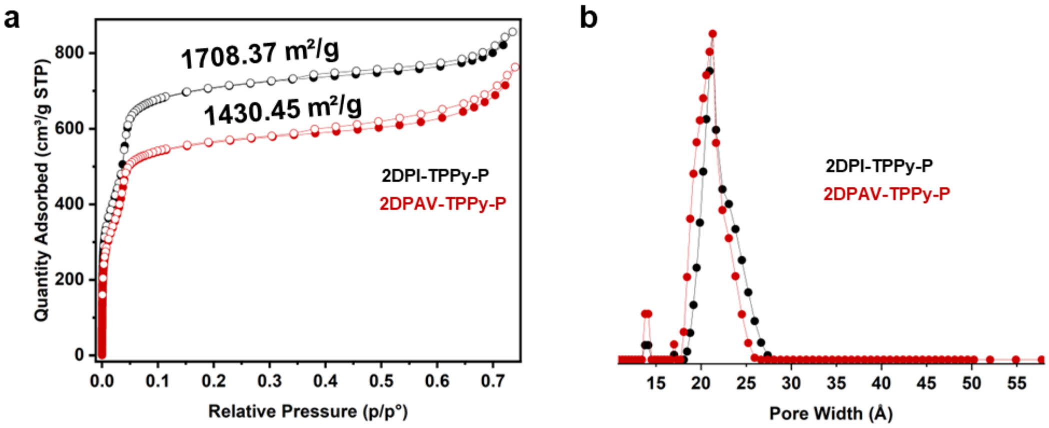

Supplementary Figure 45. a, N_2 adsorption–desorption isotherms of **2DPI-TPPy-P** and **2DPAV-TPPy-P**. **b**, Pore size distributions of **2DPI-TPPy-P** and **2DPAV-TPPy-P**.

The crystallinity of **2DPI-TPPy-P** is retained in the fully transformed **2DPAV-TPPy-P**, which also exhibits a uniform pore size distribution, its noteworthy the surface area for directly synthesized 2DPAV-TPPy-P[31] is 692 m^2 g^{-1}.

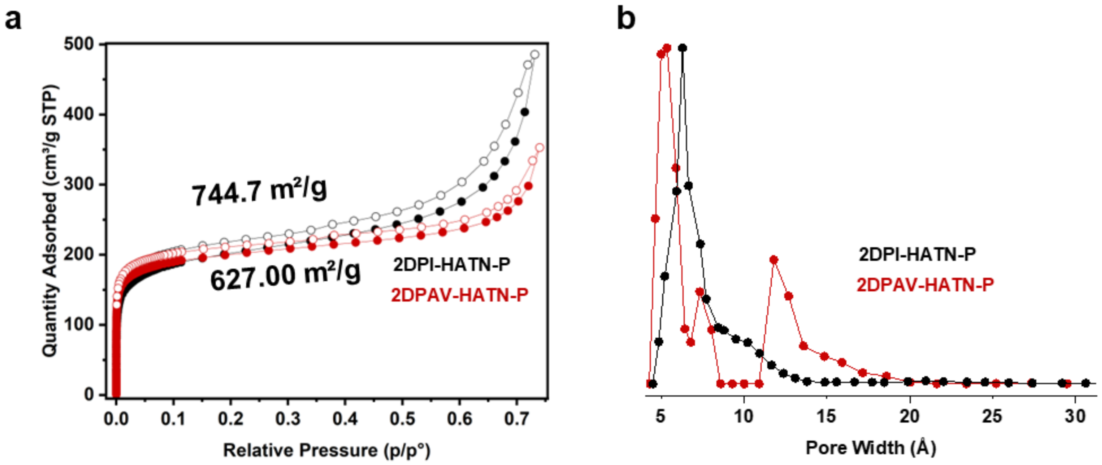

Supplementary Figure 46. **a**, N_2 adsorption–desorption isotherms of **2DPI-HATN-P** and **2DPAV-HATN-P**. **b**, Pore size distributions of **2DPI-HATN-P** and **2DPAV-HATN-P.**

The crystallinity of **2DPI-HATN-P** is preserved in the fully transformed **2DPAV-HATN-P**, which also exhibits a uniform pore size distribution, its noteworthy the surface area for the directly synthesized 2DPAV-HATN-P[28] is 317 m^2 g^{-1}.

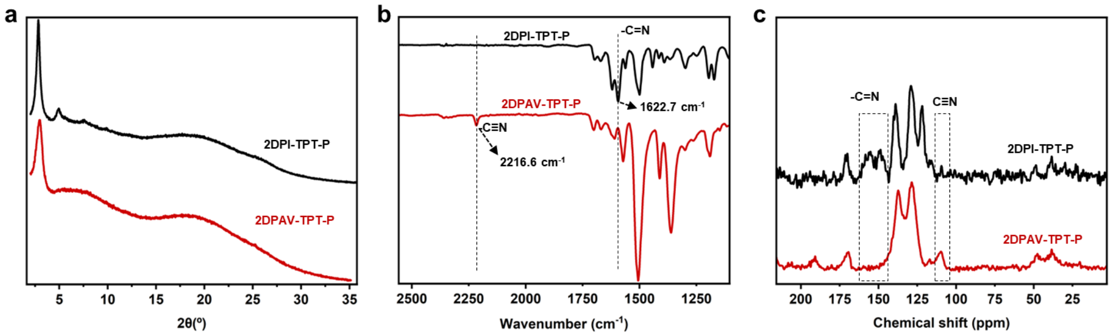

Supplementary Figure 47. **a**, PXRD, **b**, FT-IR and **c**, ^{13}C CP NMR spectrum of pristine **2DPI-TPT-P** (black line) and fully transformed **2DPAV-TPT-P** (red line).

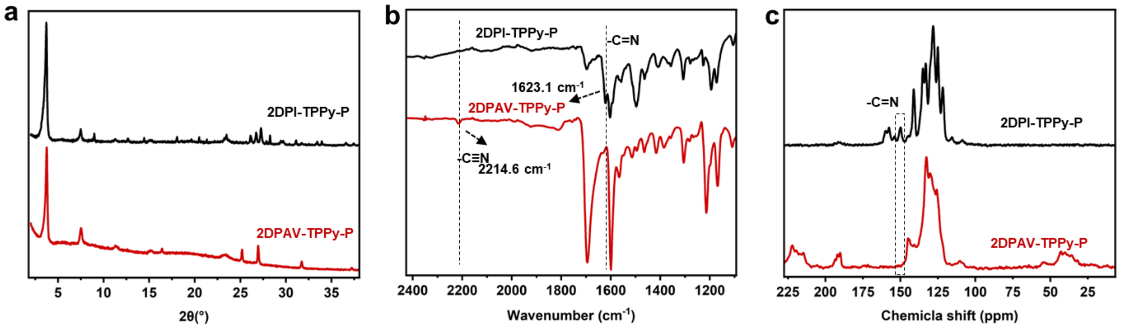

Supplementary Figure 48. **a**, PXRD, **b**, FT-IR and **c**, ^{13}C CP NMR spectrum of pristine **2DPI-TPPy-P** (black line) and fully transformed **2DPAV-TPPy-P** (red line).

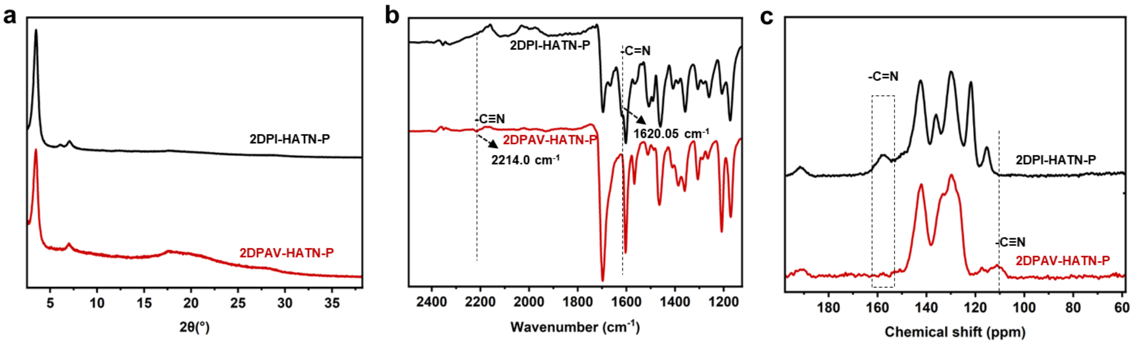

Supplementary Figure 49. **a**, PXRD, **b**, FT-IR and **c**, ^{13}C CP NMR spectrum of pristine **2DPI-HATN-P** (black line) and fully transformed **2DPAV-HATN-P** (red line).

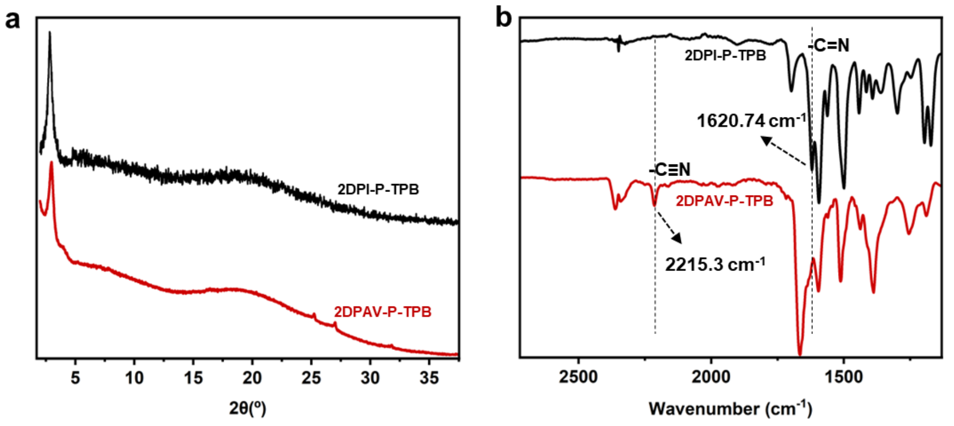

Supplementary Figure 50. **a**, PXRD and **b**, FT-IR spectrum of pristine **2DPI-P-TPB** (black line) and fully transformed **2DPAV-P-TPB** (red line).

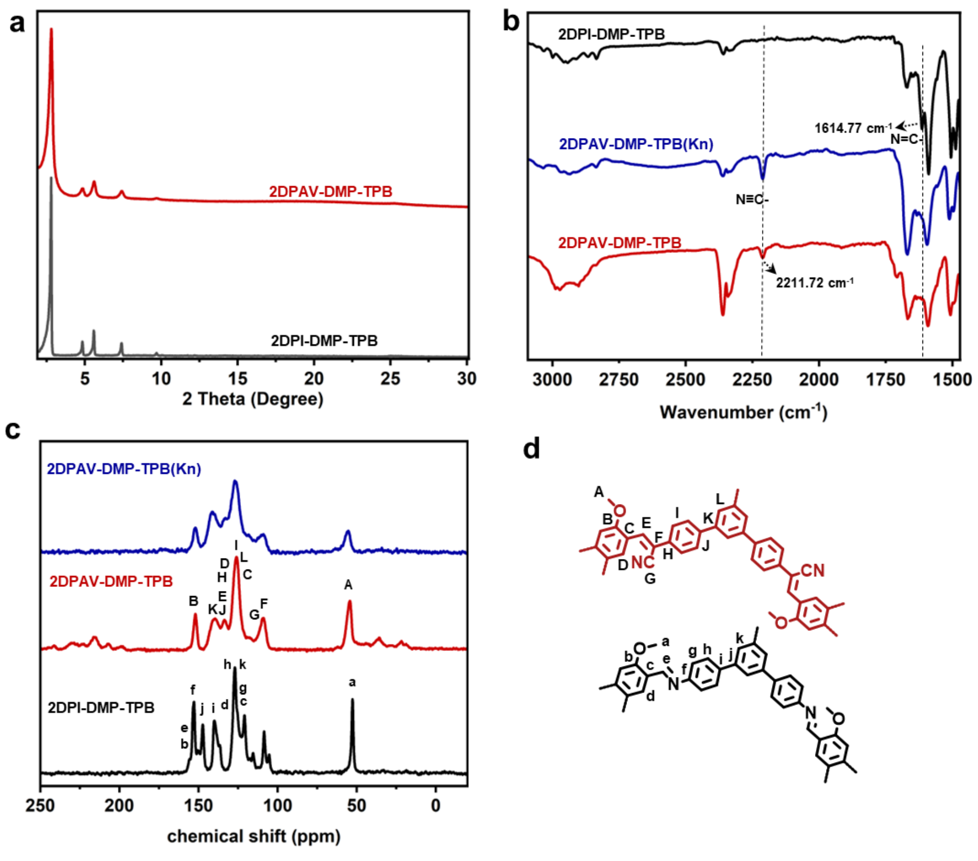

Supplementary Figure 51. **a,** PXRD, **b**, FT-IR and **c**, ^{13}C CP NMR spectrum of pristine **2DPI-DMP-TPB** (black line) and fully transformed **2DPAV-DMP-TPB** (red line). **d**, Molecular fragment of the corresponding materials, with labeled carbons highlighted.

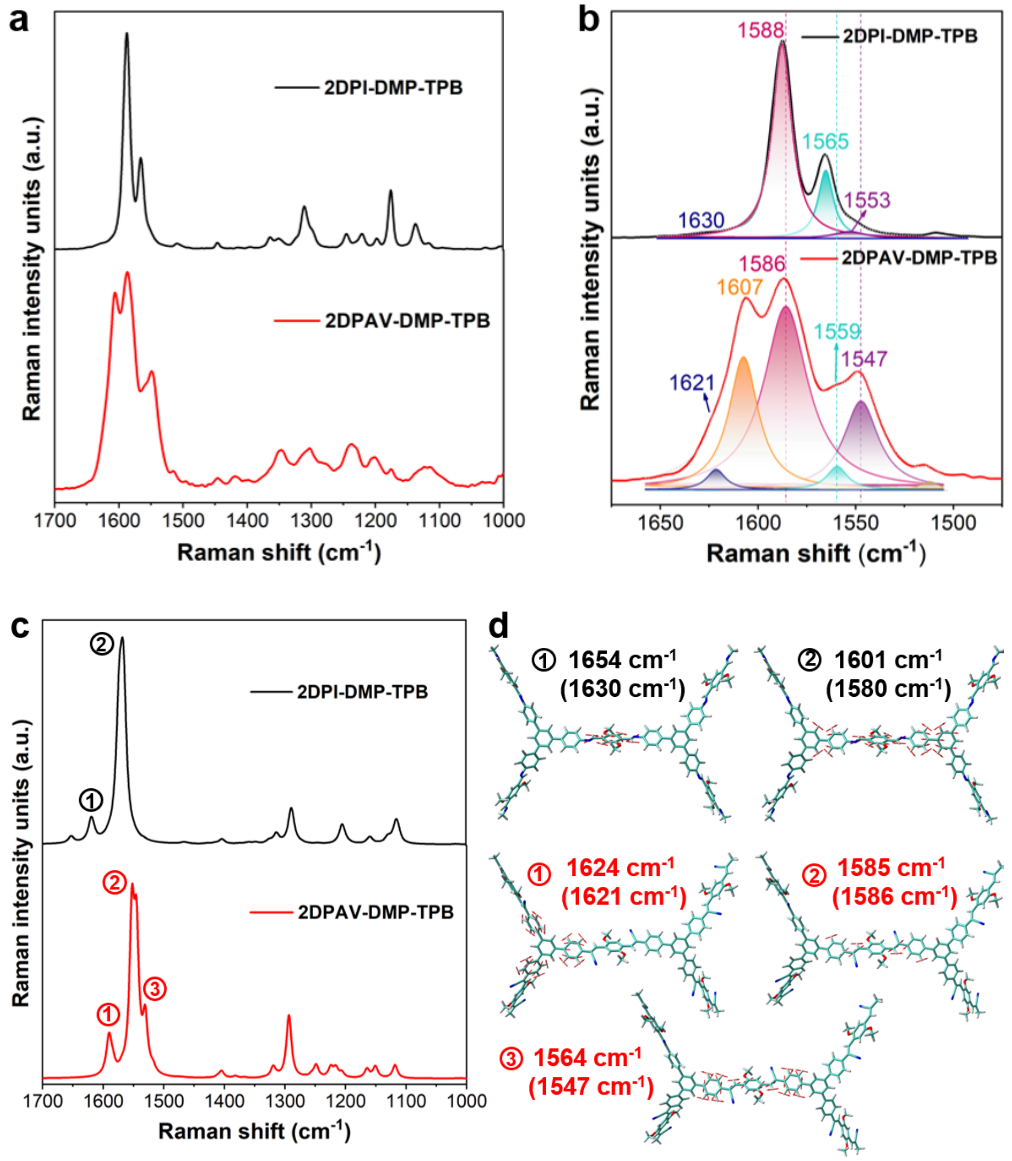

Supplementary Figure 52. **a**, FT-Raman spectrum of **2DPI-DMP-TPB** (black line) and **2DPAV-DMP-TPB** (red line). **b**, Deconvolution of the spectruml region between 1650 and 1500 cm^{-1} by using a Lorentzian fitting. **c**, DFT-simulated Raman spectrum of **2DPI-DMP-TPB** (black line) and **2DPAV-DMP-TPB** (red line). **d**, Vibrational eigenvectors associated to the most outstanding Raman features of Vibrational eigenvectors associated with the most outstanding Raman features of **2DPI-DMP-TPB** (black numbers) and **2DPAV-DMP-TPB** (red numbers). The experimental and theoretical (in parentheses) wavenumbers are also shown.

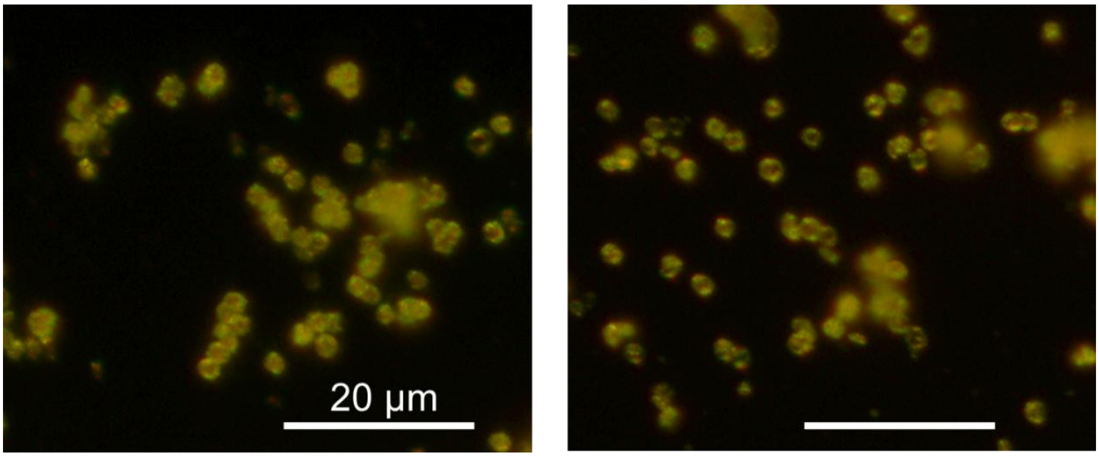

Supplementary Figure 53. single-crystalline images of **2DPI-DMP-TPB**.

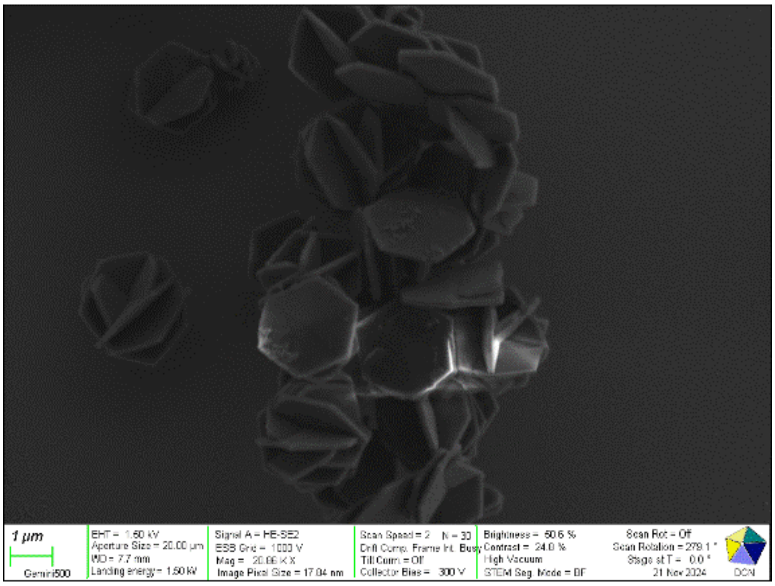

Supplementary Figure 54. SEM image of pristine **2DPI-DMP-TPB**.

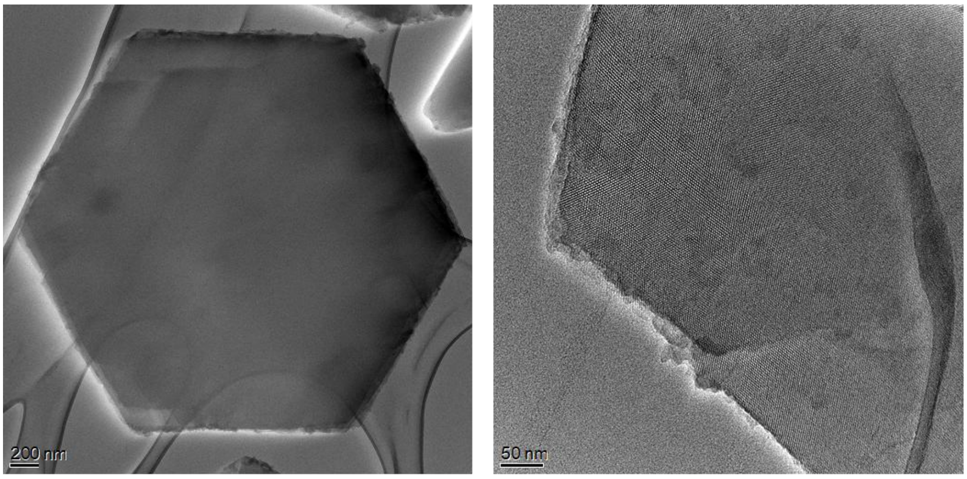

Supplementary Figure 55. TEM image of pristine **2DPI-DMP-TPB**.

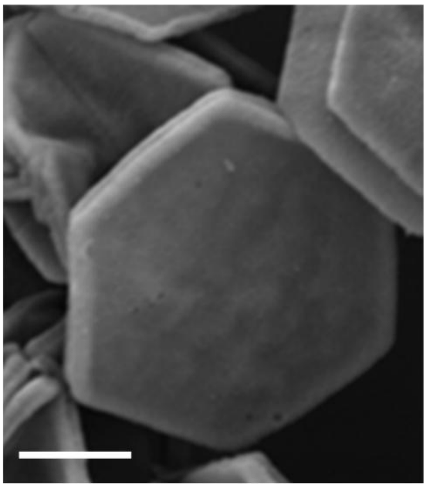

Supplementary Figure 56. SEM image of the **2DPAV-DMP-TPB**. Scale bar = 1 μm.

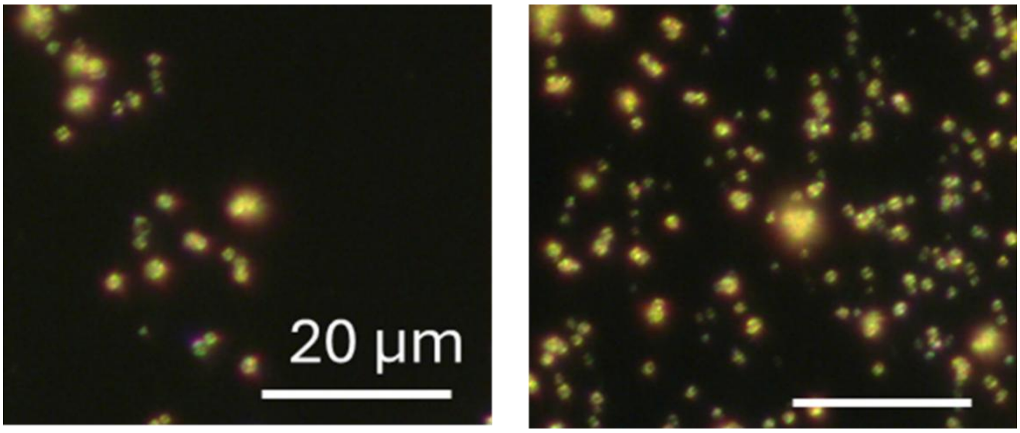

Supplementary Figure 57. single-crystalline images of **2DPAV-DMP-TPB**.

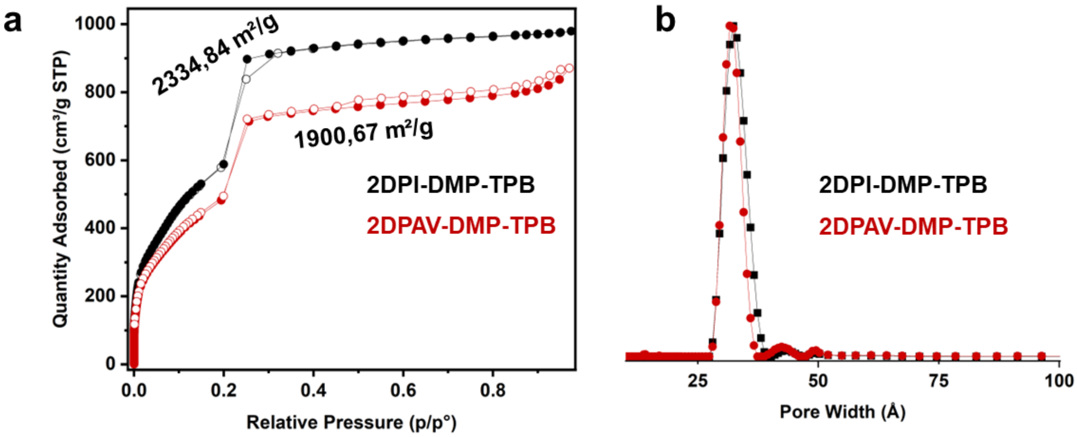

Supplementary Figure 58. a, N_2 adsorption–desorption isotherms of **2DPI-DMP-TPB** and **2DPAV-DMP-TPB**. **b**, Pore size distributions of **2DPI-DMP-TPB** and **2DPAV-DMP-TPB.**

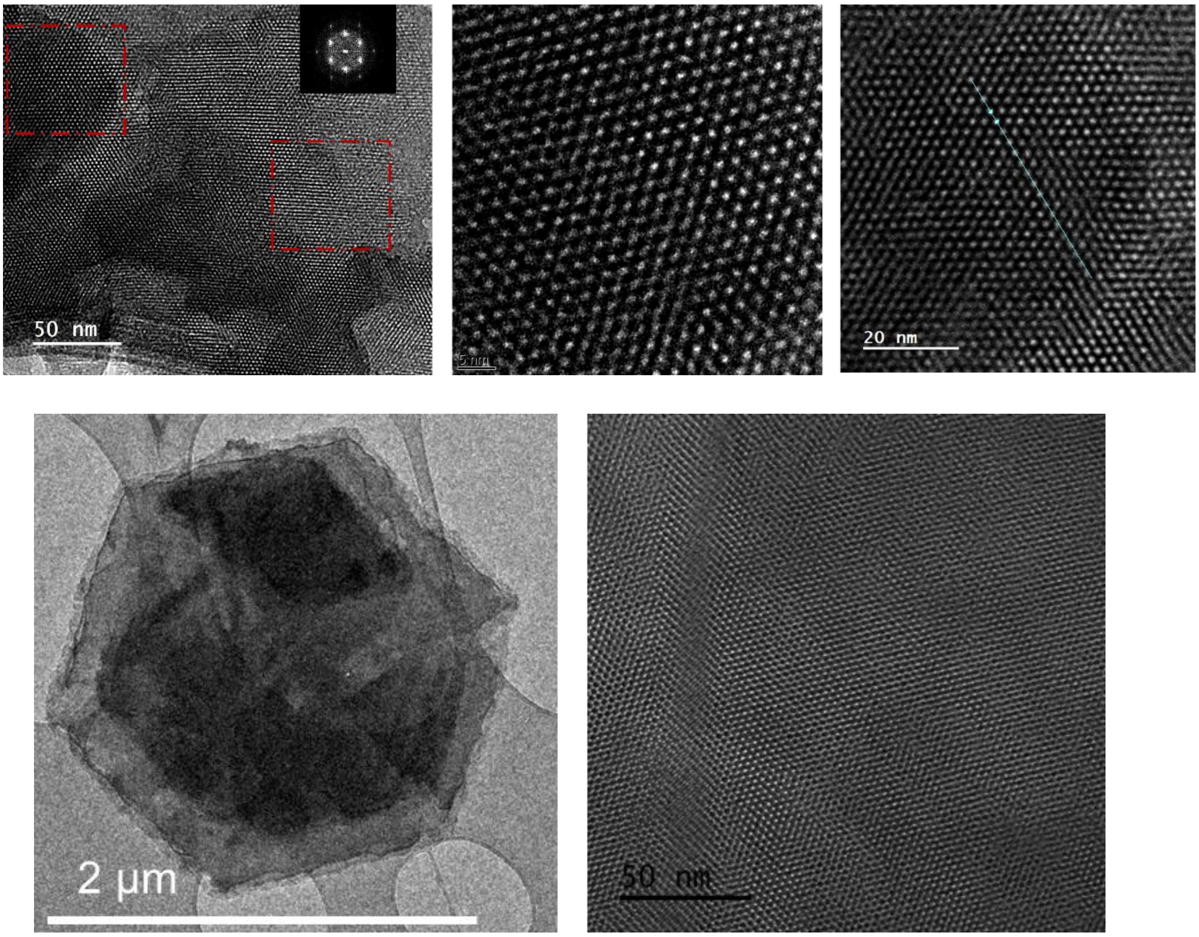

Supplementary Figure 59. TEM images of **2DPAV-DMP-TPB.**

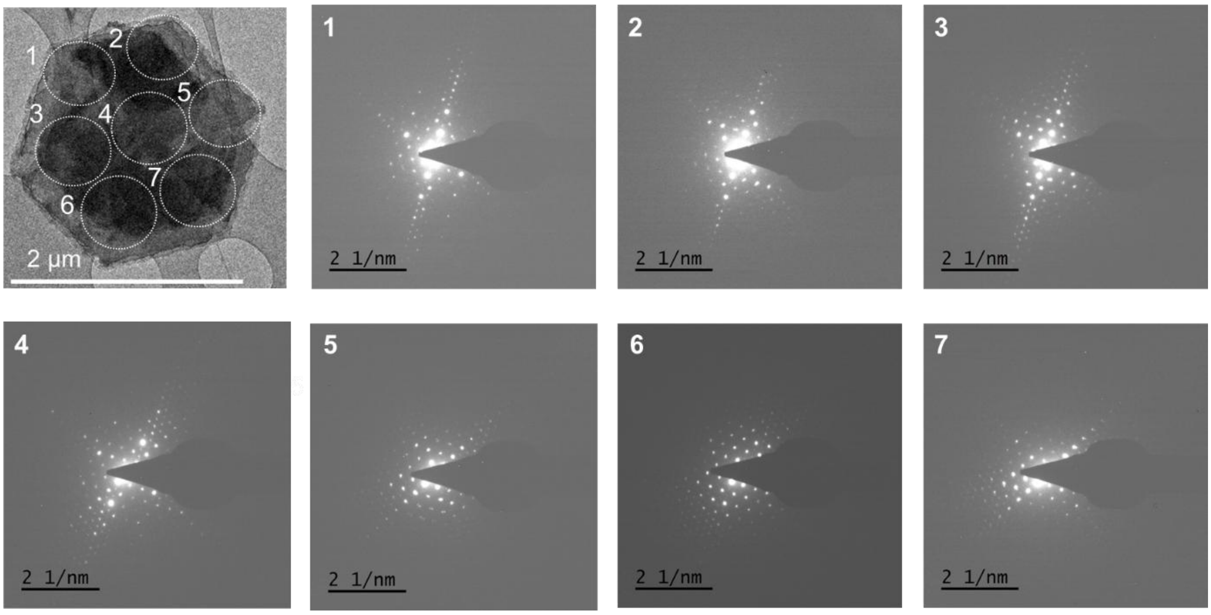

Supplementary Figure 60. SAED patterns of the selected areas(1-7) marked with white circles for **2DPAV-DMP-TPB.**

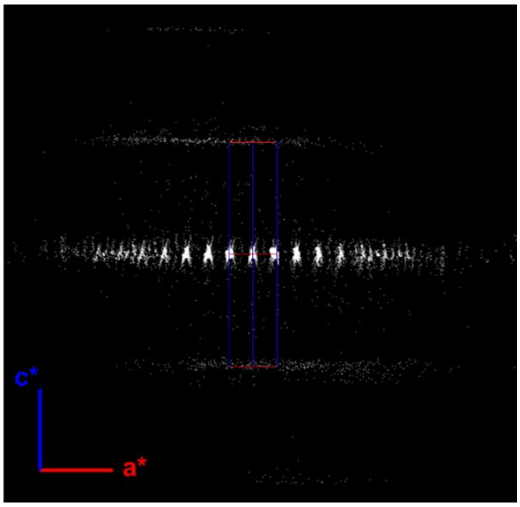

Supplementary Figure 61. 3D reciprocal lattices for **2DPAV-DMP-TPB** along *c** axis**.**

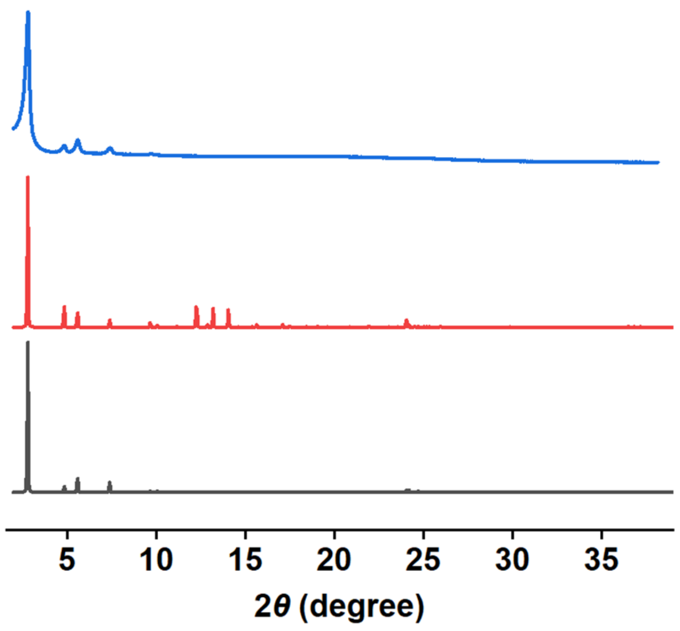

Supplementary Figure 62. Comparison of experimental diffraction data with the simulated patterns for periodic models of **2DPAV-DMP-TPB**, considering both AB- and AA-stacking configurations.

The high crystallinity of **2DPAV-DMP-TPB** was revealed by PXRD analysis. As shown in Supplementary Fig. 62, the pattern exhibited several intense diffraction peaks, which could be indexed to a hexagonal unit cell ($a = b = 36.48$ Å, $c = 3.53$ Å, $\beta = 120°$). The parameter c was evaluated by the broaden peak at $2\theta \approx 25.2°$, which suggested an eclipsed AA-stacking model with Honeycomb (**hcb**) topology. To further determine the crystal structure, continues rotation electron diffraction (cRED) data were collected at a low-dose mode at 99 K, and a hexagonal unit cell ($a = b = 36.55$ Å, c = 7.13 Å, $\beta = 120°$) was reconstructed by REDp program. Notably, the interlayer distance observed from cRED was twice that expected for an eclipsed AA-stacking model, indicating a doubling of periodicity along the c-axis. Although the AB-stacking mode was also considered, it was inconsistent with both the experimental PXRD pattern and the pore size distribution. Based on the geometric characteristics of the monomers, we proposed that the stacking was not purely eclipsed AA mode due to the steric hindrance of methoxy and cyano functional groups. Instead, the adjacent layers likely adopt a flipped form of AA-stacking, denoted as AA'-stacking (Fig. 5d). This model preserved the geometry of AA-stacking while accounting for the doubled periodicity observed in the cRED. Moreover, energy calculations by universal force field (UFF) method confirmed that the proposed AA'-stacking mode was energetically favorable, with an energy reduction of 264.34 kJ/mol compared to the eclipsed AA-stacking model. Finally, a more accurate structure model ($a = 36.505(4)$ Å, c = 7.094(8) Å, $\alpha = \beta = 90°$, $\gamma = 120°$, Space group $P6/mcc$) was obtained by the Rietveld refinement using the program Topas V5 with low residuals ($R_{wp} = 3.66\%$, $R_p = 2.47\%$).

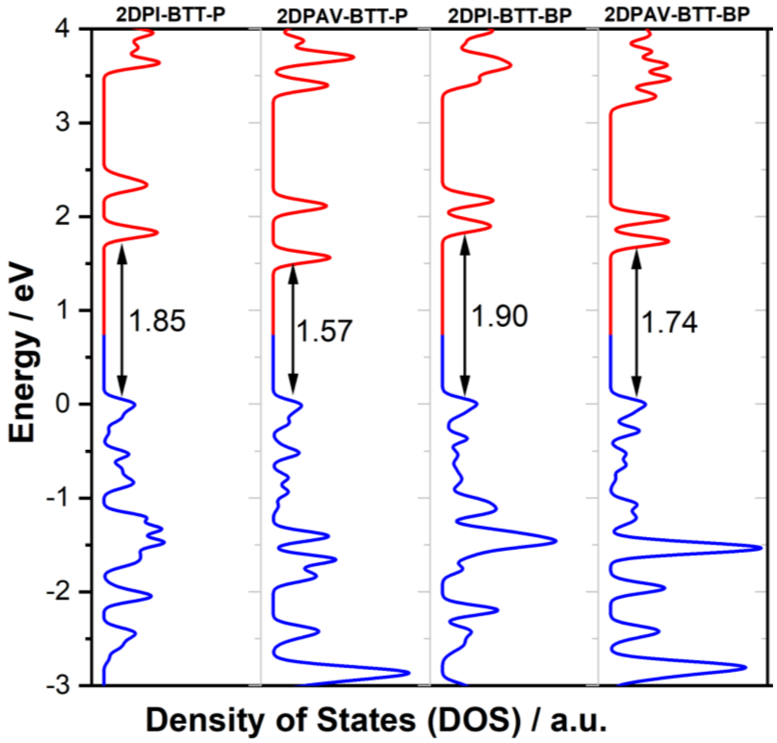

Supplementary Figure 63. DFT-PBE computed total density of electronic states profiles (in arbitrary units) as a function of the energy (referred to the Fermi energy) for BTT based 2D polymers under study.

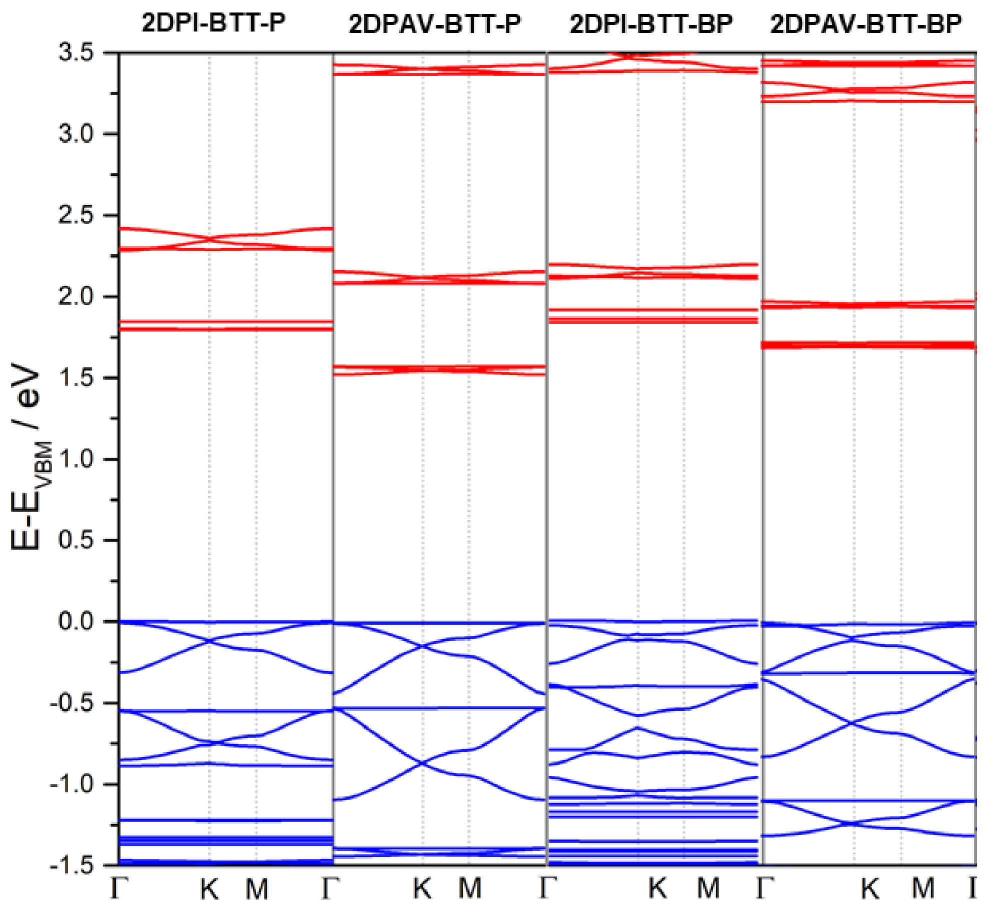

Supplementary Figure 64. DFT-PBE-calculated electronic band structures for BTT-based 2D polymers under study. The valence and conduction bands are marked in blue and red, respectively. The zero energy is set at the valence band maximum, E_{VBM}, while the x-axis labels denote a path through the 3D space of k-vectors. Points of high symmetry in the Brillouin zone are labeled as Γ(0, 0, 0), K(1/3, 2/3, 0), and M(0, 1/2, 0), all in reciprocal space crystal coordinates.

a

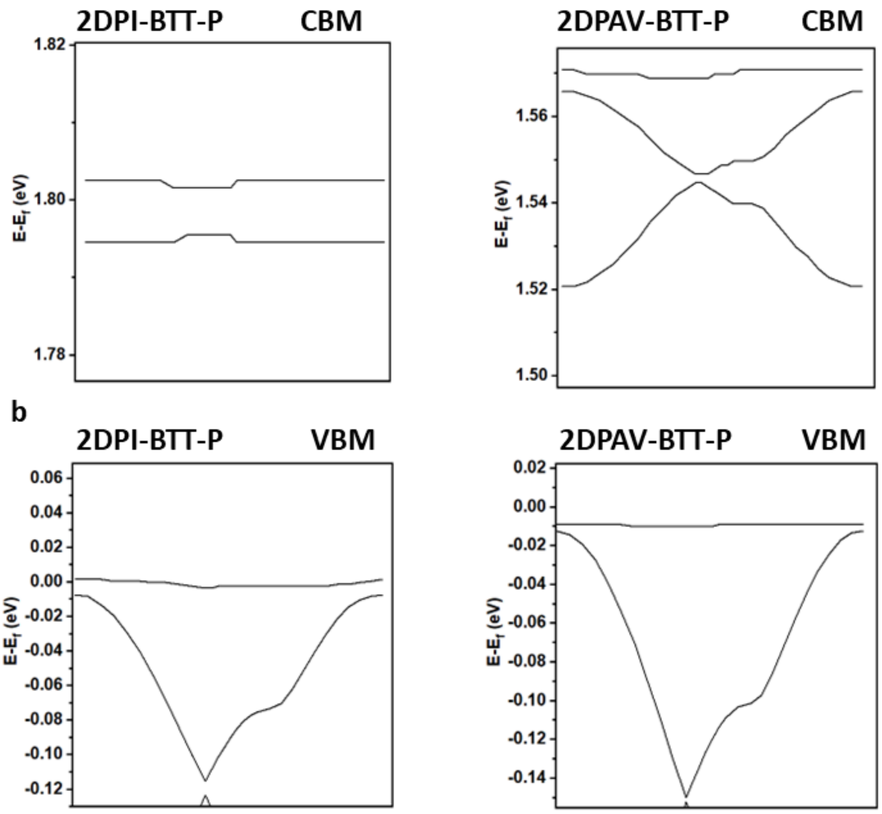

c

Property	2DPI-BTT-P	2DPAV-BTT-P
VBM Dispersion (eV)	0.005	0.001
CBM Dispersion (eV)	0.0009 (flat)	0.025

Supplementary Figure 65. a,b, Zoom-in conduction band minimum (CBM) and valence band maximum (VBM) of **2DPI-BTT-P** and **2DPAV-BTT-P** respectively. **c,** Table showing VBM Dispersion and CBM Dispersion of **2DPI-BTT-P** and **2DPAV-BTT-P** respectively.

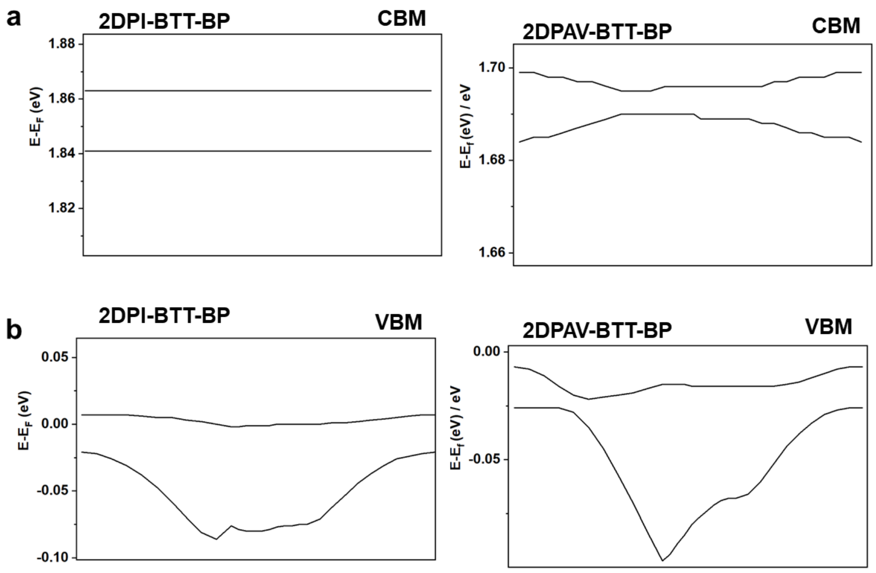

c

Property	2DPI-BTT-BP	2DPAV-BTT-BP
VBM Dispersion (eV)	0.005	0.015
CBM Dispersion (eV)	Flat	0.006

Supplementary Figure 66. a,b, Zoom-in conduction band minimum (CBM) and valence band maximum (VBM) of **2DPI-BTT-BP** and **2DPAV-BTT-BP** respectively. **c,** Table showing VBM Dispersion and CBM Dispersion of **2DPI-BTT-BP** and **2DPAV-BTT-BP** respectively.

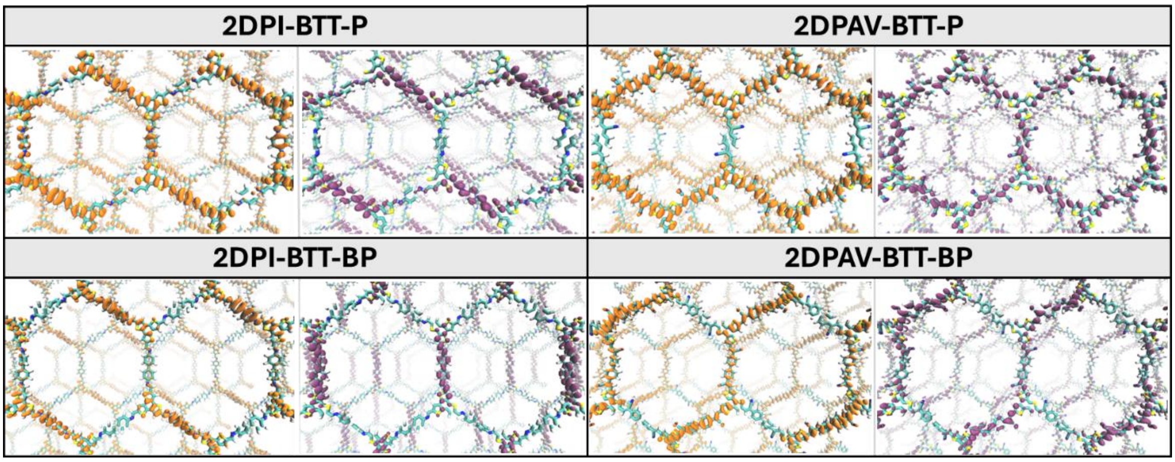

Supplementary Figure 67. DFT-PBE computed topologies of the valence (orange) and conduction (purple) bands for **2DPI-BTT-P**, **2DPAV-BTT-P**, **2DPI-BTT-BP**, and **2DPAV-BTT-BP** under study.

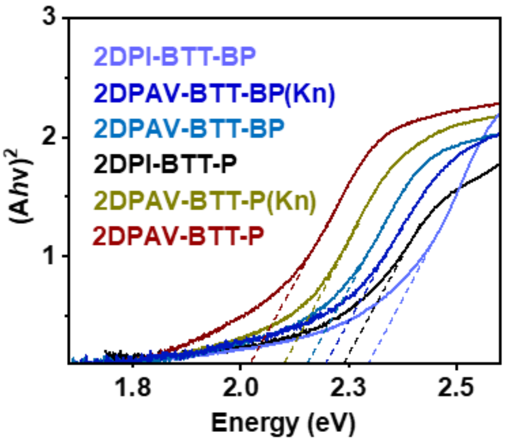

Supplementary Figure 68. Tau-plot of various BTT-based 2D polymers.

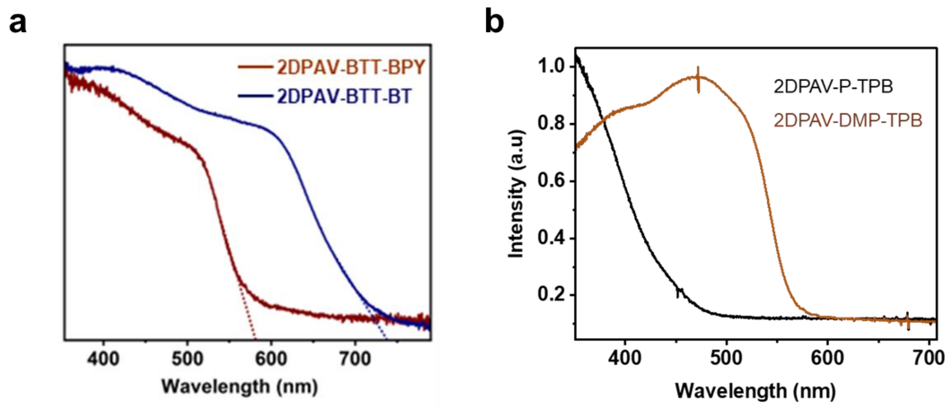

Supplementary Figure 69. UV-visible absorption spectrum of **a,** 2DPAV-BTT-BPY (brown line) and 2DPAV-BTT-BT (blue line). **b, 2DPAV-P-TPB** and **2DPAV-DMP-TPB**.

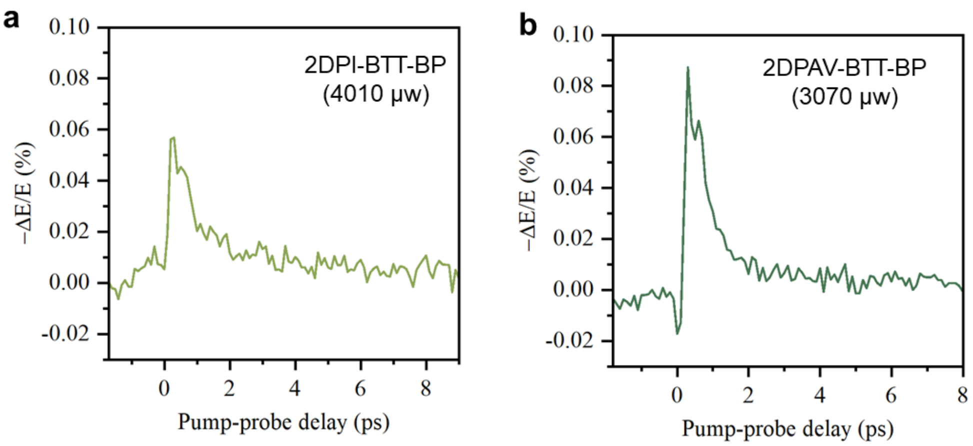

Supplementary Figure 70. Time-resolved THz photoconductivity of 2D polymers films. **a**, Time-dependent THz photoconductivity dynamics of **2DPI-BTT-BP** following 3.1 eV photoexcitation. **b**, Pump-fluence dependent THz photoconductivity dynamics of **2DPAV-BTT-BP** following 3.1 eV photoexcitation.

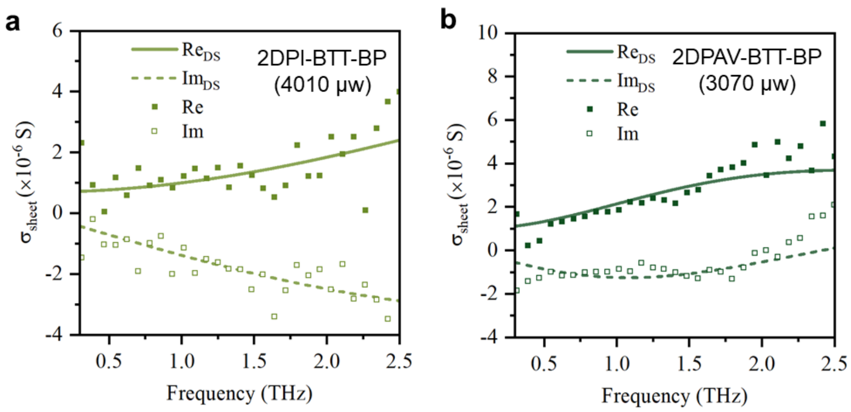

Supplementary Figure 71. Frequency-resolved THz photoconductivity spectrum of 2D polymers. **a**, Frequency-resolved complex THz photoconductivity at different delay times (1 ps) for **2DPI-BTT-BP**. The light green and Dark green solid lines represent the Drude-Smith fits describing the real and imaginary components of the complex THz photoconductivity, respectively. The photoconductivity spectrum indicates a scattering time of 17 ± 4.3 fs and a parameter c of -0.95 ± 0.00, considering $m^* = 1m_e$, gives rise to a carrier mobility of 1.49 $cm^2V^{-1}s^{-1}$. **b**, Frequency-resolved complex THz photoconductivity at different delay times (1 ps) for **2DPAV-BTT-BP**. The light green and Dark green solid lines represent the Drude-Smith fits describing the real and imaginary components of the complex THz photoconductivity, respectively. The photoconductivity spectrum indicates a scattering time of 56.4 ± 4.8 fs and a parameter c of -0.87 ± 0.03, considering $m^* = 1m_e$, gives rise to a carrier mobility of 12.89 $cm^2V^{-1}s^{-1}$.

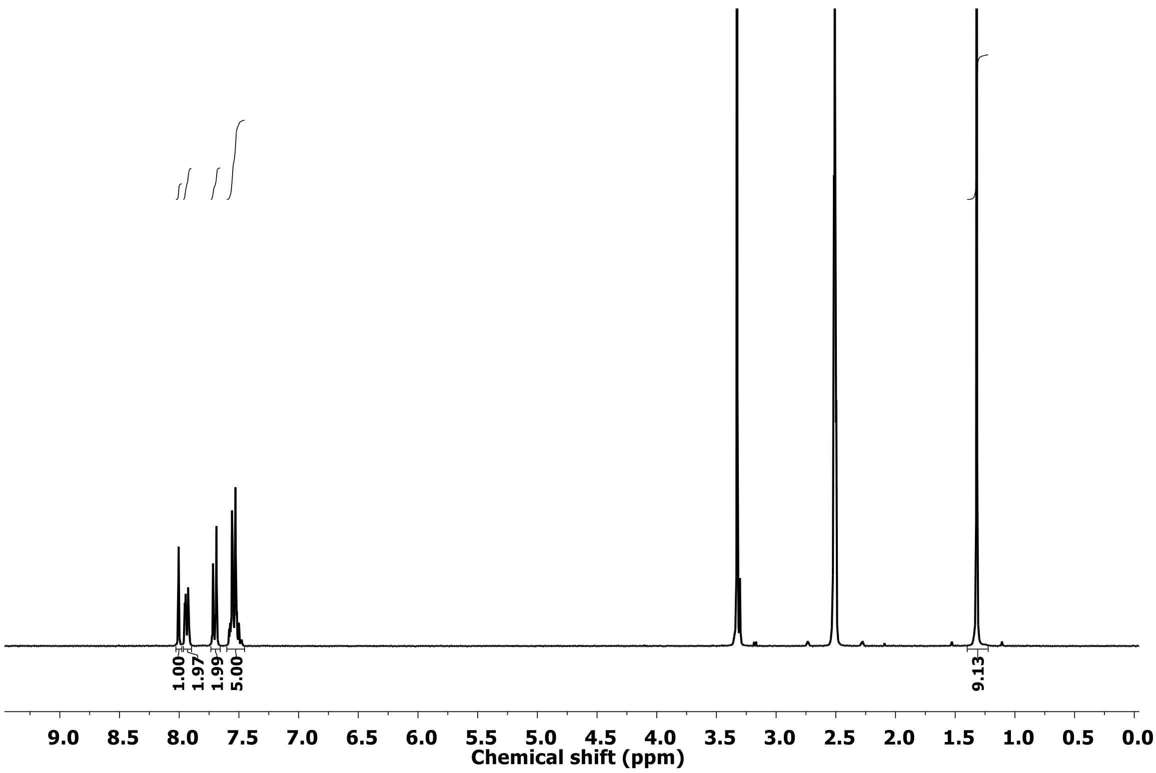

Supplementary Figure 72. ^{1}H NMR spectrum (300 MHz) of **3** in DMSO-d_6 solvent.

Section D. Supplementary Tables

Supplementary Table 1. Optimization study for the Mannich-elimination reaction between compounds **1** and **2** to yield compound **3**. All reactions were conducted over a fixed reaction time of 8 hours under the specified conditions.

**Entry**	**Solvent**	**Reagent (H_2O)**	**Catalyst**	**Temperature (°C)**	**Yield (%)**
1	DMAc	-	2eq-Cs_2CO_3	120	**98**
2	DMAc	-	2eq-KOH	120	**98**
3	DMAc	0.1 mL	2eq-Cs_2CO_3	120	**98**
4	DMAc	0.1 mL	2eq-KOH	120	**86**
5	DMAc	0.1 mL	2eq-CsOH	120	**94**
6	DMAc	0.1 mL	2eq-NaOH	120	**75**
7	DMAc	0.1 mL	2eq-K_2CO_3	120	**33**
8	DMAc	0.1 mL	2eq-Na_2CO_3	120	**10**
9	DMAc	0.1 mL	2eq-pTSA	120	**-**
10	DMAc	0.1 mL	2eq-AcOH	120	**-**
11	DMAc	0.1 mL	2eq-TFA	120	**-**
12	DMAc	0.1 mL	2eq-CsOAc	120	**30**
13	DMAc	0.1 mL	2eq-NaOAc	120	**9**
14	DMAc	0.1mL	2eq-KOAc	120	**18**
15	DMAc	0.1 mL	DBU	120	**57**
16	MeCN	0.1 mL	2eq-Cs_2CO_3	120	**36**
17	DMF	0.1 mL	2eq-Cs_2CO_3	120	**18**
18	BuOH	0.1 mL	2eq-Cs_2CO_3	120	**60**
19	DMI	0.1 mL	2eq-Cs_2CO_3	120	**91**
20	NMP	0.1 mL	2eq-Cs_2CO_3	120	**58**
21	1,4-Dioxane	0.1 mL	2eq-Cs_2CO_3	120	**01**
22	*o*-DCB	0.1mL	2eq-Cs_2CO_3	120	**-**
24	mesitylene	0.1mL	2eq-Cs_2CO_3	120	**-**

Supplementary Table 2. Fractional atomic coordinates and the unit cell of **2DPAV-DMP-TPB** generated from the Rietveld refinement.

**2DPAV-DMP-TPB**		Space Group: *P6/mcc*<br>$a = b = 36.505(4)$ Å, $c = 7.094(8)$ Å,<br>$\alpha = \beta = 90°$, $\gamma = 120°$			
Name	Atom type	x	y	z	occ
C1	C	0.4734(6)	0.4565(6)	0.5	1
C2	C	0.4557(6)	0.4826(6)	0.5	1
C3	C	0.4830(6)	0.5264(6)	0.5	1
C4	C	0.4640(6)	0.5530(7)	0.5	1
C5	C	0.4817(6)	0.5950(7)	0.5	1
C6	C	0.4524(6)	0.6114(7)	0.5	1
C7	C	0.4669(6)	0.6549(8)	0.5	1
C8	C	0.4379(5)	0.6691(8)	0.5	1
C9	C	0.3944(5)	0.6398(8)	0.5	1
C10	C	0.3801(5)	0.5966(7)	0.5	1
C11	C	0.4087(5)	0.5826(7)	0.5	1
C12	C	0.3633(4)	0.6537(8)	0.5	1
C13	C	0.3203(4)	0.6239(8)	0.5	1
O14	O	0.4116(5)	0.4663(6)	0.5	1
C15	C	0.3862(5)	0.4213(5)	0.5	1
C16	C	0.5270(6)	0.6234(8)	0.5	1
N17	N	0.5632(7)	0.6462(8)	0.5	1

Section D. Supplementary References

1. Madsen, J. & Susi, T. The abTEM code: transmission electron microscopy from first principles. *Open Research Europe* **1** (2021).
2. Gutzler, R. & Perepichka, D. F. π-Electron Conjugation in Two Dimensions. *J. Am. Chem. Soc.* **135**, 16585–16594 (2013).
3. Baryshnikov, G. V., Minaev, B. F., Karaush, N. N. & Minaeva, V. A. The art of the possible: computational design of the 1D and 2D materials based on the tetraoxa[8]circulene monomer. *RSC Adv.* **4**, 25843–25851 (2014).
4. Li, L. *et al.* Rational Design of Porous Conjugated Polymers and Roles of Residual Palladium for Photocatalytic Hydrogen Production. *J. Am. Chem. Soc.* **138**, 7681–7686 (2016).
5. Albacete, P. *et al.* Layer-Stacking-Driven Fluorescence in a Two-Dimensional Imine-Linked Covalent Organic Framework. *J. Am. Chem. Soc.* **140**, 12922–12929 (2018).
6. Thomas, S. *et al.* Design and synthesis of two-dimensional covalent organic frameworks with four-arm cores: prediction of remarkable ambipolar charge-transport properties. *Mater. Horiz.* **6**, 1868–1876 (2019).
7. Echeverri, M. *et al.* Effect of the Linkage Position on the Conjugation Length of Truxene-Based Porous Polymers: Implications for Their Sensing Performance of Nitroaromatics. *Chem. Mater.* **31**, 6971–6978 (2019).
8. Royuela, S. *et al.* Oxygen reduction using a metal-free naphthalene diimide-based covalent organic framework electrocatalyst. *Chem. Commun.* **56**, 1267–1270 (2020).
9. Gámez-Valenzuela, S., Echeverri, M., Gómez-Lor, B., Martínez, J. I. & Delgado, M. C. R. In silico design of 2D polymers containing truxene-based platforms: insights into their structural and electronic properties. *J. Mater. Chem. C* **8**, 15416–15425 (2020).
10. Lee, C., Yang, W. & Parr, R. G. Development of the Colle-Salvetti correlation-energy formula into a functional of the electron density. *Phys. Rev. B* **37**, 785–789 (1988).
11. Becke, A. D. Density-functional thermochemistry. III. The role of exact exchange. *J. Chem. Phys.* **98**, 5648–5652 (1993).
12. Perdew, J. P., Ernzerhof, M. & Burke, K. Rationale for mixing exact exchange with density functional approximations. *J. Chem. Phys.* **105**, 9982–9985 (1996).

13. Hehre, W. J., Ditchfield, R. & Pople, J. A. Self—Consistent Molecular Orbital Methods. XII. Further Extensions of Gaussian—Type Basis Sets for Use in Molecular Orbital Studies of Organic Molecules. *J. Chem. Phys.* **56**, 2257–2261 (1972).
14. Gaussian.com. https://gaussian.com/citation/.
15. Chemcraft - Graphical program for visualization of quantum chemistry computations. https://www.chemcraftprog.com/.
16. Giannozzi, P. *et al.* QUANTUM ESPRESSO: a modular and open-source software project for quantum simulations of materials. *J. Phys. Condens. Matter* **21**, 395502 (2009).
17. Perdew, J. P., Burke, K. & Ernzerhof, M. Generalized Gradient Approximation Made Simple. *Phys. Rev. Lett.* **77**, 3865–3868 (1996).
18. Mounet, N. & Marzari, N. First-principles determination of the structural, vibrational and thermodynamic properties of diamond, graphite, and derivatives. *Phys. Rev. B* **71**, 205214 (2005).
19. Rappe, A. M., Rabe, K. M., Kaxiras, E. & Joannopoulos, J. D. Optimized pseudopotentials. *Phys. Rev. B* **41**, 1227–1230 (1990).
20. Grimme, S. Semiempirical GGA-type density functional constructed with a long-range dispersion correction. *J. Comput. Chem.* **27**, 1787–1799 (2006).
21. Pack, J. D. & Monkhorst, H. J. 'Special points for Brillouin-zone integrations'---a reply. *Phys. Rev. B* **16**, 1748–1749 (1977).
22. Parrinello, M. & Rahman, A. Polymorphic transitions in single crystals: A new molecular dynamics method. *J. Appl. Phys.* **52**, 7182–7190 (1981).
23. Wentzcovitch, R. M. Invariant molecular-dynamics approach to structural phase transitions. *Phys. Rev. B* **44**, 2358–2361 (1991).
24. Parrinello, M. & Rahman, A. Crystal Structure and Pair Potentials: A Molecular-Dynamics Study. *Phys. Rev. Lett.* **45**, 1196–1199 (1980).
25. Humphrey, W., Dalke, A. & Schulten, K. VMD: Visual molecular dynamics. *J. Mol. Graph.* **14**, 33–38 (1996).
26. Yang, M. *et al.* Multibranched Octupolar Module Embedded Covalent Organic Frameworks Enable Efficient Two-Photon Fluorescence. *Adv. Funct. Mater.* **30**, 2000516 (2020).
27. Li, X. *et al.* Regioselective One-Step Cyclization and Aromatization towards Directly Amino-Functionalized Covalent Organic Framework with Stable Benzodiimidazole Linkage. *Small* **19**, 2303775 (2023).

28. Xu, S. *et al.* A Nitrogen-Rich 2D sp2-Carbon-Linked Conjugated Polymer Framework as a High-Performance Cathode for Lithium-Ion Batteries. *Angew. Chem. Int. Ed.* **131**, 859–863 (2019).
29. Jin, E. *et al.* Topology-Templated Synthesis of Crystalline Porous Covalent Organic Frameworks. *Angew. Chem. Int. Ed.* **59**, 12162–12169 (2020).
30. Xu, Q., Tao, S., Jiang, Q. & Jiang, D. Designing Covalent Organic Frameworks with a Tailored Ionic Interface for Ion Transport across One-Dimensional Channels. *Angew. Chem. Int. Ed.* **59**, 4557–4563 (2020).
31. Jin, E. *et al.* Two-dimensional sp2 carbon–conjugated covalent organic frameworks. *Science* **357**, 673–676 (2017).
